# Exploring the Molecular Mechanism of the Drug-Treated Breast Cancer Based on Gene Expression Microarray

**DOI:** 10.3390/biom9070282

**Published:** 2019-07-15

**Authors:** Ali Mohamed Alshabi, Basavaraj Vastrad, Ibrahim Ahmed Shaikh, Chanabasayya Vastrad

**Affiliations:** 1 Department of Clinical Pharmacy, College of Pharmacy, Najran University, Najran 66237, Saudi Arabia; 2 Department of Pharmaceutics, SET’S College of Pharmacy, Dharwad, Karnataka 580002, India; 3 Department of Pharmacology, College of Pharmacy, Najran University, Najran 66237, Saudi Arabia; 4 Biostatistics and Bioinformatics, Chanabasava Nilaya, Bharthinagar, Dharwad, Karnataka 580001, India

**Keywords:** pathway enrichment analysis, protein-protein interaction network, microRNA

## Abstract

Breast cancer (BRCA) remains the leading cause of cancer morbidity and mortality worldwide. In the present study, we identified novel biomarkers expressed during estradiol and tamoxifen treatment of BRCA. The microarray dataset of E-MTAB-4975 from Array Express database was downloaded, and the differential expressed genes (DEGs) between estradiol-treated BRCA sample and tamoxifen-treated BRCA sample were identified by limma package. The pathway and gene ontology (GO) enrichment analysis, construction of protein-protein interaction (PPI) network, module analysis, construction of target genes—miRNA interaction network and target genes-transcription factor (TF) interaction network were performed using bioinformatics tools. The expression, prognostic values, and mutation of hub genes were validated by SurvExpress database, cBioPortal, and human protein atlas (HPA) database. A total of 856 genes (421 up-regulated genes and 435 down-regulated genes) were identified in T47D (overexpressing Split Ends (SPEN) + estradiol) samples compared to T47D (overexpressing Split Ends (SPEN) + tamoxifen) samples. Pathway and GO enrichment analysis revealed that the DEGs were mainly enriched in response to lysine degradation II (pipecolate pathway), cholesterol biosynthesis pathway, cell cycle pathway, and response to cytokine pathway. DEGs (*MCM2*, *TCF4*, *OLR1*, *HSPA5*, *MAP1LC3B*, *SQSTM1*, *NEU1*, *HIST1H1B*, *RAD51*, *RFC3*, *MCM10*, *ISG15*, *TNFRSF10B*, *GBP2*, *IGFBP5*, *SOD2*, *DHF* and *MT1H*), which were significantly up- and down-regulated in estradiol and tamoxifen-treated BRCA samples, were selected as hub genes according to the results of protein-protein interaction (PPI) network, module analysis, target genes—miRNA interaction network and target genes-TF interaction network analysis. The SurvExpress database, cBioPortal, and Human Protein Atlas (HPA) database further confirmed that patients with higher expression levels of these hub genes experienced a shorter overall survival. A comprehensive bioinformatics analysis was performed, and potential therapeutic applications of estradiol and tamoxifen were predicted in BRCA samples. The data may unravel the future molecular mechanisms of BRCA.

## 1. Introduction 

Breast cancer (BRCA) is the most common type of gynecological cancer in women [1]. BRCA accounts for 2,088,849 (11.6%)of new cancer cases [2] and 626,679 (6.6%) deaths in women worldwide, as per 2018 cancer statistics [3].Surgical resection is an effective treatment to advance patient survival time [4], but it is only suitable for a small percentage of all cases [5].A number of other therapies, including radiotherapy [6], chemotherapy [7], hormone therapy [8], and immunotherapy [9], have been developed for BRCA treatment; however, there is limited information regarding the long-term survival rate, and the mortality rate of BRCA patients remains high [10]. Therefore, examinations into new treatment strategies for patients with BRCA are needed.

Gene therapy and small molecule drugs are new strategies for cancer treatment, which have gained increasing consideration over the past few decades [11]. Currently, a number of studies have been conducted to know the underlying molecular mechanisms and find treatment targets for BRCA [12]. Specific genes associated with the DNA damage response, including BRCA1, are mutated during the development of BRCA [13]. Wang et al. [14], Gong et al. [15], Serra et al. [16], Ghayad et al. [17], and Lemée et al. [18] had verified, through microarray and RT-PCR technology, the role of VEGFR-2, AKT, HER, and MAPK as well as activated the replication and genomic instability in BRCA. The Cyclin D1 gene is overexpressed in BRCA and may act as a therapeutic target [19]. Previous studies have mainly concentrated on a certain gene or pathway; therefore, it is necessary to search the underlying molecular mechanisms and therapeutic targets for BRCA using other methods.

In the current study, the expression-profiling data E-MTAB-4975 was downloaded, and the differential expressed genes (DEGs) were analyzed between T47D (overexpressing Split Ends (SPEN)+ estradiol) samples and T47D (overexpressing Split Ends (SPEN) + tamoxifen) samples. The functions of DEGs were analyzed using pathway and gene ontology (GO) enrichment analysis. Furthermore, protein-protein interactions (PPIs) of DEGs were investigated and the topological properties of hub genes calculated as well as modules were extracted in the PPI network. In addition, target gene –miRNA interaction network and target gene- transcription factor (TF) interaction network were constructed. Therefore, the current study analyzed their expression data using a series of bioinformatics methods to diagnose important associated and novel biomarkers, that will allow the identification of the underlying mechanisms associated with BRCA.

## 2. Materials and Methods

### 2.1. Agilent Microarray Data

The microarray dataset E-MTAB-4975 was downloaded from the ArrayExpress [20] and analyzed using the Agilent 028004 SurePrint G3 Human GE 8x60K Microarray (Agilent Technologies, Inc., Santa Clara, CA, USA) platform. A total of 18 samples were present in this dataset, including 3 T47D (wild type genotype + estradiol), 3 T47D (wild type genotype + none), 3 T47D (wild type genotype + tamoxifen), 3 T47D (overexpressing Split Ends (SPEN) + estradiol), 3 T47D (overexpressing Split Ends (SPEN) +none), and 3 T47D (overexpressing Split Ends (SPEN) + tamoxifen) samples.

### 2.2. Data Preprocessing

Raw probe-level data was downloaded, and expression profile data preprocessing was performed based on the limma package (version 3.34.9); in R Bioconductor version 3.4.4 [20]. The matrix data of dataset achieved log2 conversion and normalization applying limma package of R/Bioconductor software [21]. 

### 2.3. Identification of DEGs

Following data preprocessing, DEGs between T47D (overexpressing Split Ends (SPEN) + estradiol) samples and T47D (overexpressing Split Ends (SPEN) + tamoxifen) samples were analyzed using Bayes methods based limma package, and raw *p*-values were revised using the Benjamini and Hochberg method [22]. The cut-off criteria for defining DEGs are *p* < 0.05, |logFC| > 1.19 (up-regulated genes), and |logFC| > −1.35 (down-regulated genes).

### 2.4. Pathway Enrichment Analyses of DEGs

The ToppGene provides functional classification and annotation analyses of associated genes [23] which integrates different pathway databases, such as BioCyc [24], Kyoto Encyclopedia of Genes and Genomes (KEGG) [25], Pathway Interaction Database (PID) [26], Reactome [27], GenMAPP [28], MSigDB C2 BioCarta (v6.0) [29], PantherDB [30], Pathway Ontology [31], and Small Molecule Pathway Database (SMPDB) [32] pathways. The significant pathways enriched with up-regulated and down-regulated DEGs were selected with a criterion of *p* < 0.05.

### 2.5. Gene Ontology (GO) Enrichment Analysis

Gene Ontology (GO) is a widely used method for consolidation of biology that compiles structured, defined, and regulated glossary for large scale gene annotation [33]. The ToppGene [23] provides a comprehensive set of functional annotation tools to identify GO terms, such as biological processes (BP), cellular component (CC), and molecular function (MF).To understand the biological functions of the DEGs, the present study used ToppGene to identify GO categories. The significant GO terms enriched with up-regulated and down-regulated DEGs were selected with a criterion of *p* < 0.05.

### 2.6. PPI Network Construction and Module Analysis

The online tool HIPPIE (Human Integrated Protein-Protein Interaction rEference) [34] integrates different PPI databases, such as IntAct [35], BioGRID [36], HPRD [37], MINT [38], BIND [39], MIPS [40], and DIP [41], which were applied to construct a PPI network and visualized using the Cytoscape software version 3.7.0 [42]. The importance of a protein in the PPI network was determined by its topological properties, such as degree (number of the proteins it connected) [43], betweenness centrality(measures the ability of a protein to monitor communication between other proteins) [44], stress centrality (number of nodes in the shortest path between two other nodes) [45], closeness centrality(inverse of the average length of the shortest paths to/from all the other nodes in the graph) [46], and cluster coefficient(measures the density of edges in the network neighborhood of a node) [47]. A node represents gene, and an edge represents a number of interactions between genes.

Module analysis was performed using the JAVA plugin PEWCC1 in Cytoscape with the threshold of *p* < 0.001 to obtain sub-networks (modules) [48]. For each hub, genes in modules were identified.

### 2.7. Construction of Target Genes-miRNA Regulatory Network

The different miRNA database, such as TarBase [49] and miRTarBase database [50], are publicly available comprehensive resource containing the predicted and the experimentally validated target gene–miRNA interaction pairs. Subsequently, the hub genes, which interact with a maximum number of miRNA, were selected. The target gene-miRNA was generated from NetworkAnalyst [51] and visualized using the Cytoscape version 3.7.0 software [42].

### 2.8. Construction of Target Genes-TF Regulatory Network

The TFs database, named ChEA database [52], provides data on eukaryotic transcription factors, consensus binding sequences (positional weight matrices), experimentally proven binding sites, and regulated genes. Target genes-TF regulatory network was generated from NetworkAnalyst [51] and visualized using the Cytoscape version 3.7.0 software [42]. Subsequently, the hub genes interacting with the maximum number of TFs were selected.

### 2.9. Survival Analysis of Hub Genes

The hub genes were identified as the intersecting genes of The Cancer Genome Atlas (TCGA) and DEGs. The hub genes were then analyzed on web tool SurvExpress, a portal for facilitating tumor subgroup gene expression and survival analyses [53]. BRCA samples were divided into two groups: (1) high expression and (2) low expression. The survival curves of samples with high gene expression and low gene expression were compared by Kaplan-Meier survival plot, the log-rank *p*-value, and hazard ratio (HR, 95% confidence intervals). *p* < 0.05 is considered statistically significant.

### 2.10. Validation of Hub Genes

The mRNA expression of the DEGs was analyzed in 2 low-risk and 1 high-risk groups with the assistance of SurvExpress [53], which is an online tool to deliver customizable functionalities based on The Cancer Genome Atlas, and the translational levels of the hub genes were validated using the Human ProteinAtlas (HPA) database [54].

### 2.11. Mutation Analysis of Hub Genes

**Figure 1:**
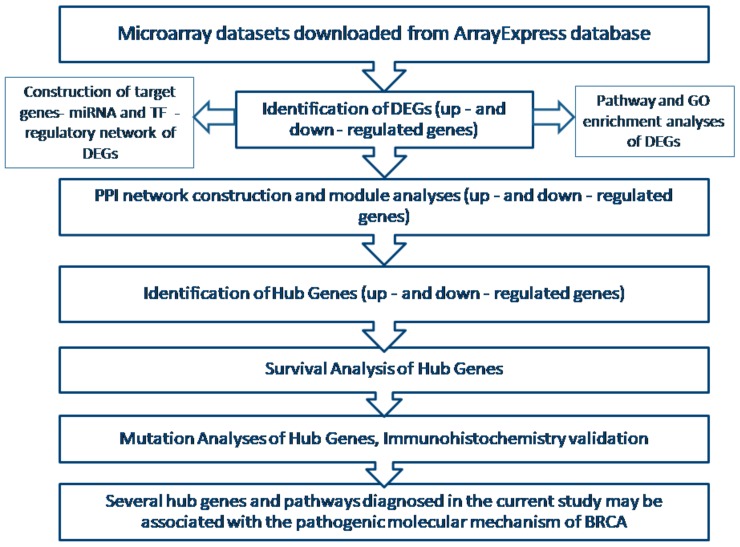
The workflow representing the methodology and the major outcome of the study. BRCA—breast cancer, GO—gene ontology, miRNA—MicroRNA, TF—transcription factor, DEGs—differential expressed genes.

The cBio Cancer Genomics Portal [55] is a web tool, which provides mutation analysis, visualization, and downloads of cancer genomics datasets of various cancers. Complex cancer genomics profiles are accessible from the cBioPortal tool, thus enabling us to compare the genetic modifications of the selected ten hub genes in BRCA. The flowchart of the methodology is depicted below (Figure 1).

## 3. Results 

### 3.1. Data Preprocessing

**Figure 2:**
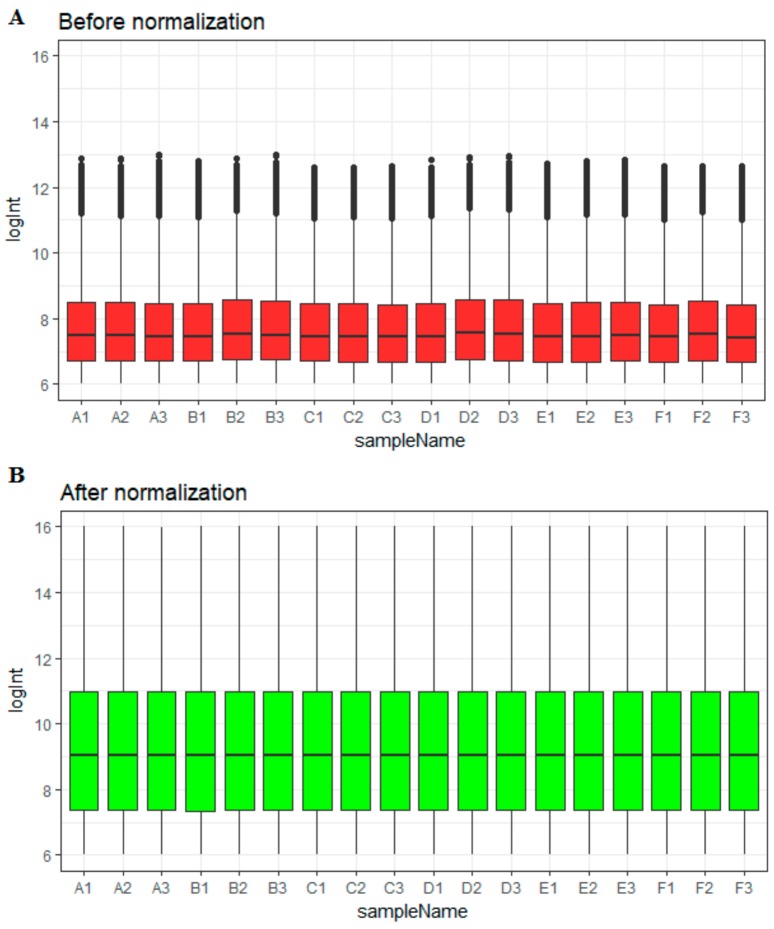
Box plots of the gene expression data before (**A**) and after (**B**) normalization. The horizontal axis represents the sample symbol, and the vertical axis represents the gene expression values. The black line in the box plot represents the median value of gene expression. (A1, A2, A3 = T47D (wild type genotype + estradiol); B1, B2, B3 = T47D (wild type genotype + none); C1, C2, C3 = T47D (wild type genotype + tamoxifen); D1, D2, D3 = T47D (overexpressing Split Ends (SPEN) + estradiol); E1, E2, E3 = T47D (overexpressing Split Ends (SPEN) + none); F1, F2, F3 = T47D (overexpressing Split Ends (SPEN) + tamoxifen)).

Before normalization, the medians of gene expression in each sample were greatly definite (Figure 2A). However, the medians became consistent and were at an identical level following normalization (Figure 2B), suggesting that the normalization process is valid, and the normalized data may be used for additional analysis. Based on their BRCA status, samples were divided into six groups: T47D (wild type genotype + estradiol) (*n* = 3), T47D (wild type genotype + none) (*n* = 3), T47D (wild type genotype + tamoxifen) (*n* = 3), T47D (overexpressing Split Ends (SPEN) + estradiol) (*n* = 3), T47D (overexpressing Split Ends (SPEN) + none) (*n* = 3), and T47D (overexpressing Split Ends (SPEN) + tamoxifen) (*n* = 3).

### 3.2. Identification of DEGs

**Figure 3:**
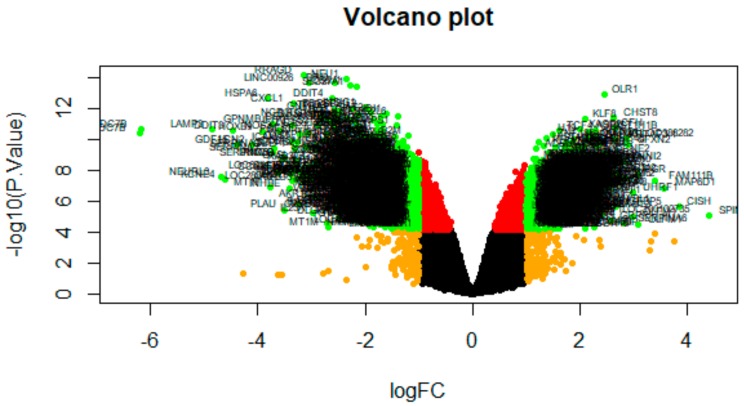
Volcano plot of differentially expressed genes. Genes with a significant change of more than two-fold were selected.

**Figure 4:**
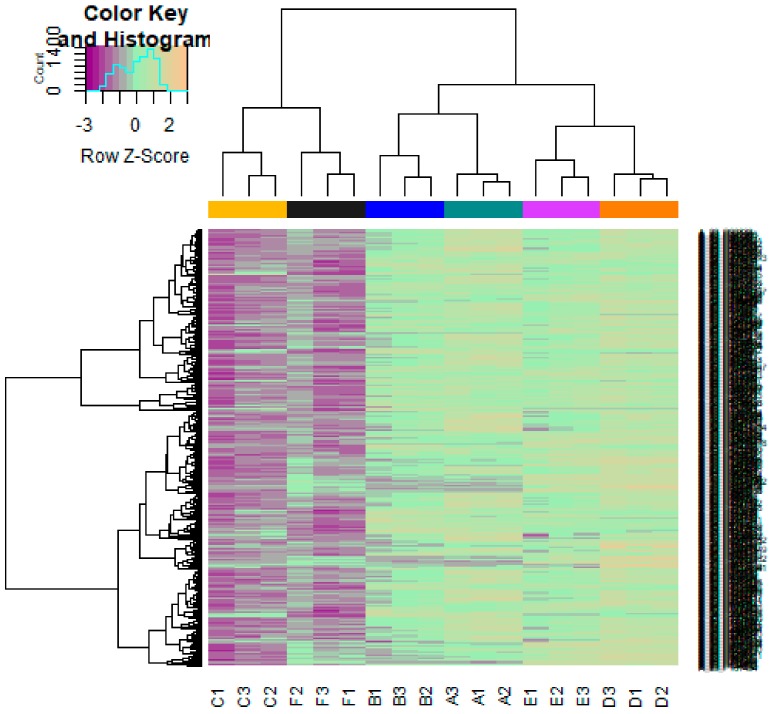
Heat map of up-regulated differentially expressed genes. The legend on the top left indicates log fold change of genes. (A1, A2, A3 = T47D (wild type genotype + estradiol); B1, B2, B3 = T47D (wild type genotype + none); C1, C2, C3 = T47D (wild type genotype + tamoxifen); D1, D2, D3 = T47D (overexpressing Split Ends (SPEN) + estradiol); E1, E2, E3 = T47D (overexpressing Split Ends (SPEN) + none); F1, F2, F3 = T47D (overexpressing Split Ends (SPEN) + tamoxifen)).

**Figure 5:**
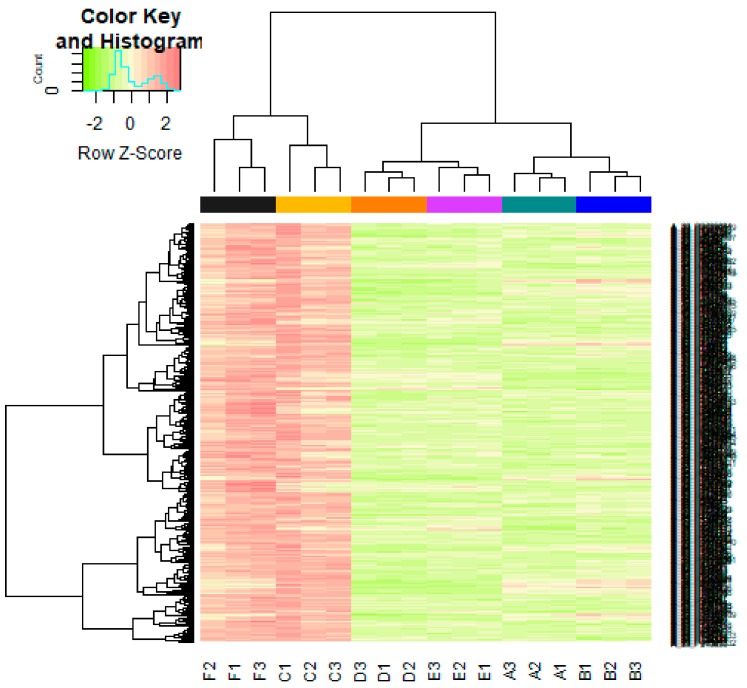
Heat map of down-regulated differentially expressed genes. The legend on the top left indicates log fold change of genes. (A1, A2, A3 = T47D (wild type genotype + estradiol); B1, B2, B3 = T47D (wild type genotype + none); C1, C2, C3 = T47D (wild type genotype + tamoxifen); D1, D2, D3 = T47D (overexpressing Split Ends (SPEN) + estradiol); E1, E2, E3 = T47D (overexpressing Split Ends (SPEN) + none); F1, F2, F3 = T47D (overexpressing Split Ends (SPEN) + tamoxifen)).

The DEGs of E-MTAB-4975 were analyzed using the limma package following preprocessing and removing batch effects. Using *p* < 0.05 and |logFC| > 1.19 as the cutoff criteria for up-regulated genes, *p* < 0.05 and |logFC)| > −1.35 for down-regulated genes, total of 856 genes (421 up-regulated genes and 435 down-regulated genes) were identified in T47D (overexpressing Split Ends (SPEN) + estradiol) samples compared to T47D (overexpressing Split Ends (SPEN) + tamoxifen) samples (Table S1). The DEGs (up- and down-regulated) are shown in the volcano plot (Figure 3). The DEGs (up- and down-regulated), according to the value of |logFC|, are visualized on a heatmap (Figure 4 and Figure 5). A total of 145 housekeeping genes were identified in this dataset. 

### 3.3. Pathway Enrichment Analysis

The DEGs (up- and down-regulated) were uploaded to the online web tool ToppGene having different pathway databases, such as BioCyc [24], KEGG [25], PID [26], Reactome [27], GenMAPP [28], MSigDB C2BioCarta [29], PantherDB [30], Pathway Ontology [31], and SMPDB [32]. Tables S2 and S3 contain the most significantly enriched pathways for up- and down-regulated genes from different pathway databases. The up-regulated genes were enriched in lysine degradation II (pipecolate pathway), pyrimidine deoxyribonucleosides salvage, DNA replication, cell cycle, E2F transcription factor network, BARD1 signaling events, mitotic, pyrimidine metabolism, carbon pool by folate, CDK regulation of DNA replication, expression of cyclins regulating progression through the cell cycle by activating cyclin-dependent kinases, tetrahydrofolate biosynthesis, mismatch repair pathway, nucleotide excision repair, and GABA-transaminase deficiency, listed in Table S2. The down-regulated genes were enriched in superpathway of cholesterol biosynthesis, cholesterol biosynthesis II (via 24,25-dihydrolanosterol), mineral absorption, squalene 2,3-epoxide => cholesterol, ATF-2 transcription factor network, direct p53 effectors, response to metal ions, sterol biosynthesis, gamma-hexachlorocyclohexane degradation, genes encoding secreted soluble factors, actions of nitric oxide in the heart, apoptosis signaling pathway, cholesterol biosynthetic, steroid biosynthetic, and risedronate pathway, listed in Table S3.

### 3.4. Gene Ontology (GO) Enrichment Analysis

The DEGs were uploaded to the online web tool ToppGene to pinpoint overrepresented GO categories. GO analysis results showed that up-regulated genes were significantly enriched in all GO terms, which include: cell cycle, DNA replication, chromosome, chromosomal part, chromosome, centromeric region, and DNA helicase activity (Table S4), while down-regulated genes were significantly enriched in all GO terms, which include: response to cytokine, cellular response to cytokine, endoplasmic reticulum, nuclear outer membrane-endoplasmic reticulum membrane network, cytokine activity, and cytokine receptor binding (Table S5).

### 3.5. PPI Network Construction and Topology Analysis

**Figure 6:**
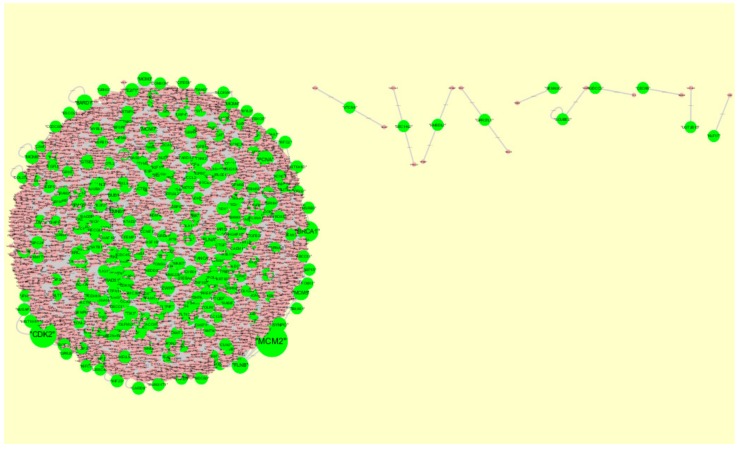
Protein-protein interaction network of differentially expressed genes (DEGs). Green nodes denote up-regulated genes.

**Figure 7:**
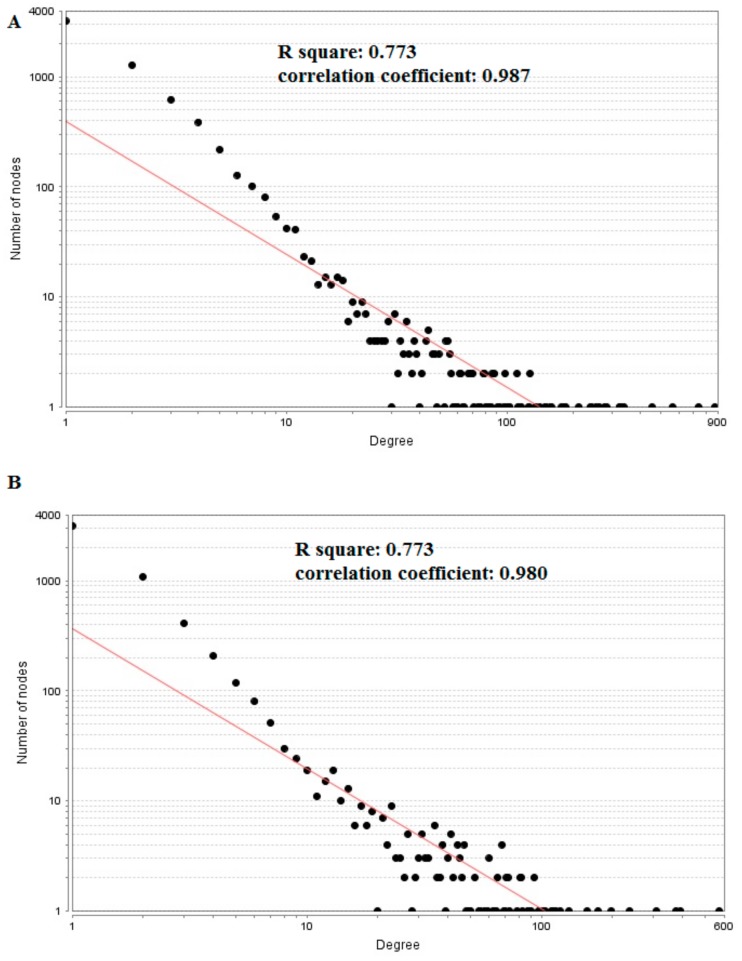
Node degree distribution. (**A**) Up-regulated genes; (**B**) Down-regulated genes.

**Figure 8:**
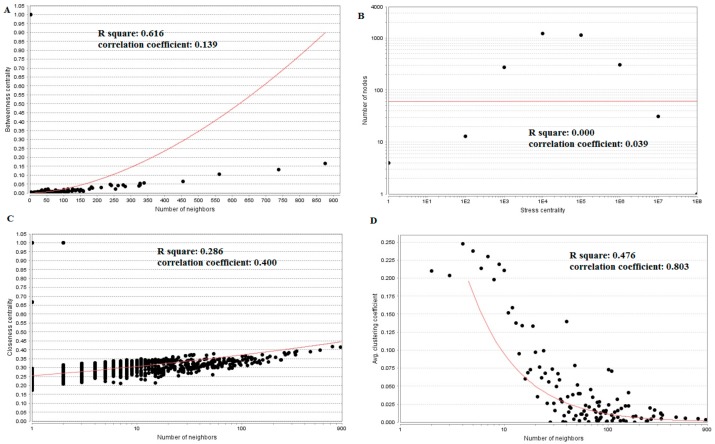
Regression diagrams for up-regulated genes (**A**) Betweenness centrality; (**B**) Stress centrality; (**C**) Closeness centrality; (**D**) Clustering coefficient.

The PPI network (up-regulated) had 6479 nodes and 15,710 interactions (Figure 6). Hub genes with high node degree, such as *MCM2* (degree = 875), *CDK2* (degree = 737), *BRCA1* (degree = 561), *HIST1H3F* (degree = 454), and *HIST1H3B* (degree = 454), are listed in Table S6. R square = 0.773 and correlation coefficient = 0.987 for node degree (Figure 7A). Hub genes with high betweenness centrality, such as *TCF4* (betweenness = 0.04852), *ASPM* (betweenness = 0.012646), *CHST8* (betweenness = 0.005764), *KCNB1* (betweenness = 0.004046), and *CFH* (betweenness = 0.003746), are listed in Table S6. R square = 0.616 and correlation coefficient = 0.139 for betweenness centrality (Figure 8A). Hub genes with high stress, such as *MCM2* (stress = 127048732), *BRCA1* (stress = 87536324), *CDK2* (stress = 84519756), *HIST1H3F* (stress = 47668690), and *HIST1H3B* (stress = 47668690), are listed in Table S6. R square = 0.000 and correlation coefficient = 0.039 for stress (Figure 8B). Hub genes with high closeness centrality, such as *MCM2* (closeness = 0.414382), *FLNA* (closeness = 0.392197), *BRCA1* (closeness = 0.398937), *HIST1H3B* (closeness = 0.388514), and *HIST1H3F* (closeness = 0.388514), are listed in Table S6. R square = 0.286 and correlation coefficient = 0.400 for closeness (Figure 8C). Hub genes with low clustering coefficient, such as *OLR1* (clustering coefficient = 0), *CHST8* (clustering coefficient = 0), *KLF8* (clustering coefficient = 0), *CFH* (clustering coefficient = 0), and *XAGE2* (clustering coefficient = 0), are listed in Table S6. R square = 0.476 and correlation coefficient = 0.803 for clustering coefficient (Figure 8D).

**Figure 9:**
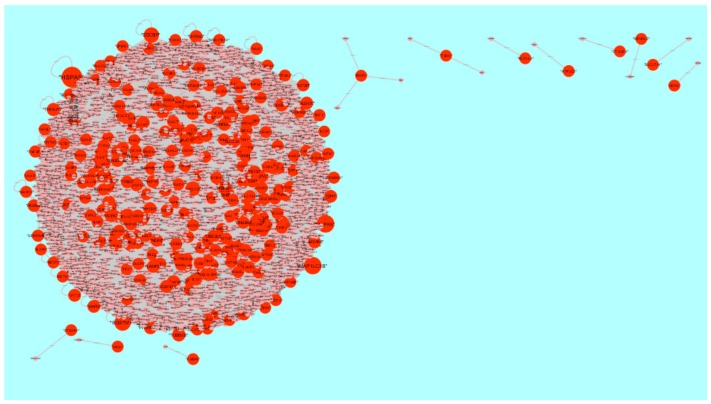
Protein-protein interaction network of differentially expressed genes (DEGs). Orange nodes denote down-regulated genes.

**Figure 10:**
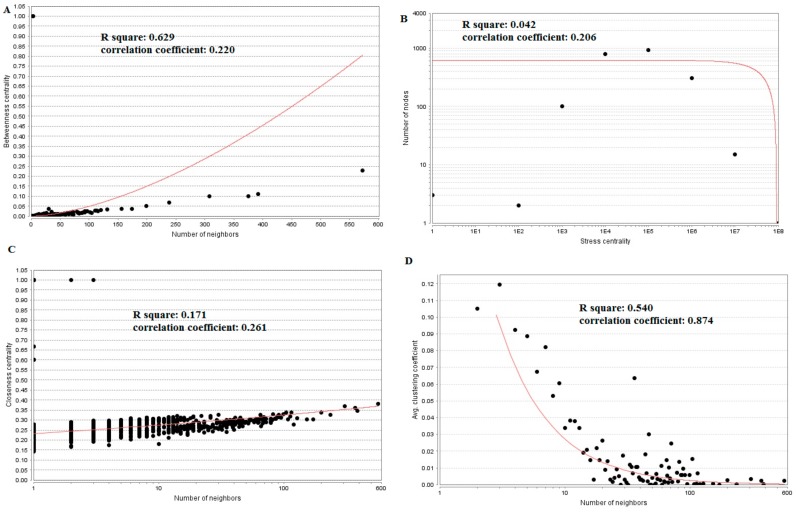
Regression diagrams for down-regulated genes (**A**) Betweenness centrality; (**B**) Stress centrality; (**C**) Closeness centrality; (**D**) Clustering coefficient.

The PPI network (down-regulated) had 5441 nodes and 9866 interactions (Figure 9). Hub genes with high node degree, such as *HSPA5* (degree = 572), *MAPK6* (degree = 392), *MAP1LC3B* (degree = 375), *SQSTM1* (degree = 308), and *SDCBP* (degree = 238), are listed in Table S6. R square = 0.773 and correlation coefficient = 0.980 for node degree (Figure 7B). Hub genes with high betweenness centrality, such as *MAP1LC3B* (betweenness = 1.01E-01), *SQSTM1* (betweenness = 9.98E-02), *SDCBP* (betweenness = 6.95E-02), *ISG15* (betweenness = 5.19E-02), and *ICAM1* (betweenness = 3.59E-02), are listed in Table S6. R square = 0.629 and correlation coefficient = 0.220 for betweenness centrality (Figure 10A). Hub genes with high stress genes, such as *HSPA5* (stress = 144471588), *MAPK6* (stress = 73838870), *MAP1LC3B* (stress = 51072854), *SQSTM1* (stress = 39034828), and *ICAM1* (stress = 38584000), are listed in Table S6. R square = 0.042 and correlation coefficient = 0.206 for stress (Figure 10B). Hub genes with high closeness centrality, such as *SQSTM1* (closeness = 0.369146), *MAP1LC3B* (closeness = 0.361868), *MAPK6* (closeness = 0.345146), *NDRG1* (closeness = 0.338184), and *ISG15* (closeness = 0.336858), are listed in Table S6. R square = 0.171 and correlation coefficient = 0.261 for closeness (Figure 10C). Hub genes with low clustering coefficient, such as *NEU1* (clustering coefficient = 0), *BRI3* (clustering coefficient = 0), *SLC27A1* (clustering coefficient = 0), *DDIT4* (clustering coefficient = 0), and *CXCL1* (clustering coefficient = 0), are listed in Table S6. R square = 0.540 and correlation coefficient = 0.874 for clustering coefficient (Figure 10D).

### 3.6. Module Analysis

**Figure 11:**
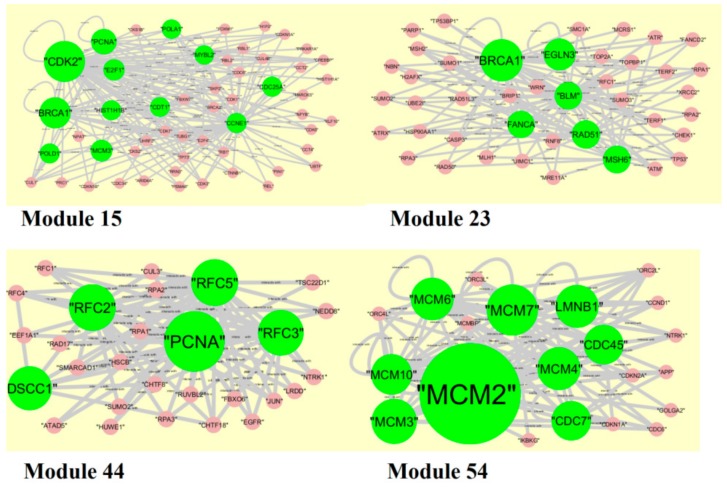
Modules in protein-protein interaction (PPI) network. The green nodes denote the up-regulated genes.

The PPI network (up-regulated genes) had 1835 modules. Module 15, module 23, module 44, and module 54 were highly significant (Figure 11). Module 15 had 53 nodes and 148 edges. The hub genes, such as *HIST1H1B* (degree = 74), *E2F1* (degree = 135), *POLD1* (degree = 79), *CDT1* (degree = 70), *MCM3* (degree = 157), *MYBL2* (degree = 54), *CDK2* (degree = 737), *CCNE1* (degree = 84), *CDC25A* (degree = 88), *PCNA* (degree = 326), *BRCA1* (degree = 561), and *POLA1* (degree = 54), were involved in module 15. Module 23 had 42 nodes and 119 edges. The hub genes, such as *RAD51* (degree = 126), *BLM* (degree = 93), *MSH6* (degree = 102), *BARD1* (degree = 277), *BRCA1* (degree = 561), and *PCNA* (degree = 326), were involved in module 23. Module 44 had 28 nodes and 88 edges. The hub genes, such as *RFC3* (degree = 48), *RFC5* (degree = 71), *RFC2* (degree = 68), *DSCC1* (degree = 20), and *PCNA* (degree = 326), were involved in module 44. Module 54 had 21 nodes and 95 edges. The hub genes, such as *MCM10* (degree = 61), *CDC7* (degree = 36), *MCM4* (degree = 111), *MCM2* (degree = 875), *MCM3* (degree = 157), *MCM6* (degree = 103), and *LMNB1* (degree = 111), were involved in module 54.

**Figure 12:**
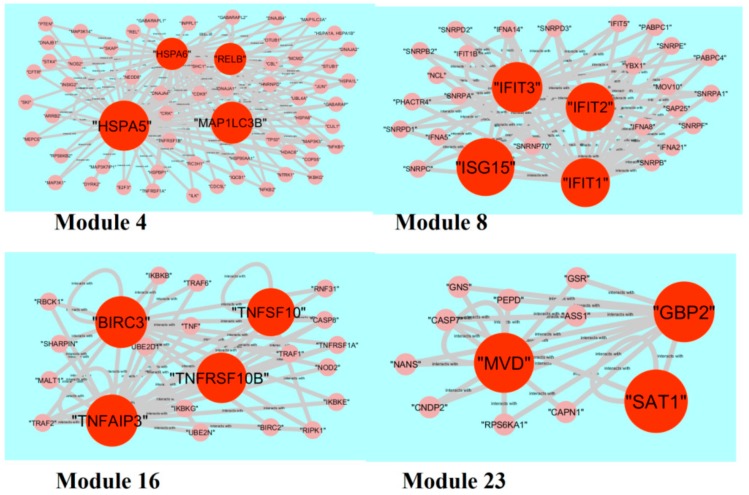
Modules in protein-protein interaction (PPI) network. The orange nodes denote the down-regulated genes.

The PPI network (down-regulated genes) had 1003 modules. Module 4, module 8, module 16, and module 23 were highly significant (Figure 12). Module 4 had 61 nodes and 140 edges. The hub genes, such as *HSPA5* (degree = 572), *HSPA6* (degree = 104), *MAP1LC3B* (degree = 375), and *RELB* (degree = 102), were involved in module 4. Module 8 had 28 nodes and 75 edges. The hub proteins, such as *ISG15* (degree = 199), *IFIT1* (degree = 47), *IFIT2* (degree = 44), and *IFIT3* (degree = 73), were involved in module 8. Module 16 had 22 nodes and 50 edges. The hub proteins, such as *TNFRSF10B* (degree = 65), *TNFSF10* (degree = 23), *TNFAIP3* (degree = 93), and *BIRC3* (degree = 88), were involved in module 16. Module 23 had 12 nodes and 23 edges. The hub proteins, such as *GBP2* (degree = 45), *SAT1* (degree = 82), and *MVD* (degree = 35), were involved in module 23.

### 3.7. Construction of the Target Genes-miRNAInteraction Network

**Figure 13:**
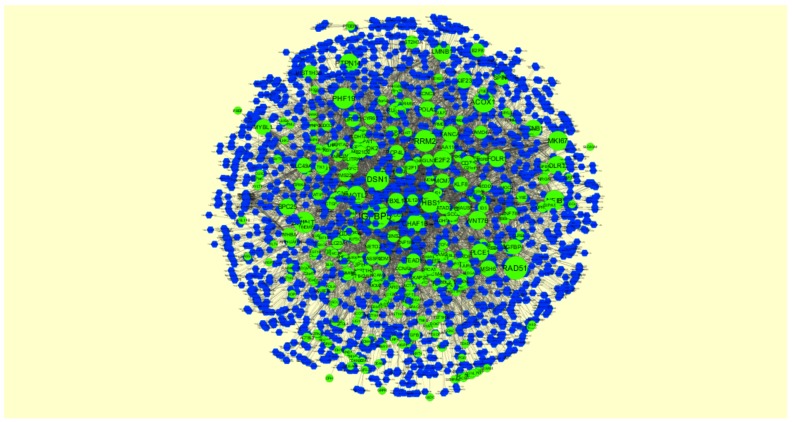
The network of up-regulated differential expressed genes (DEGs) and their related miRNAs. The green circle nodes are the up-regulated DEGs, and blue diamond nodes are the miRNAs.

**Figure 14:**
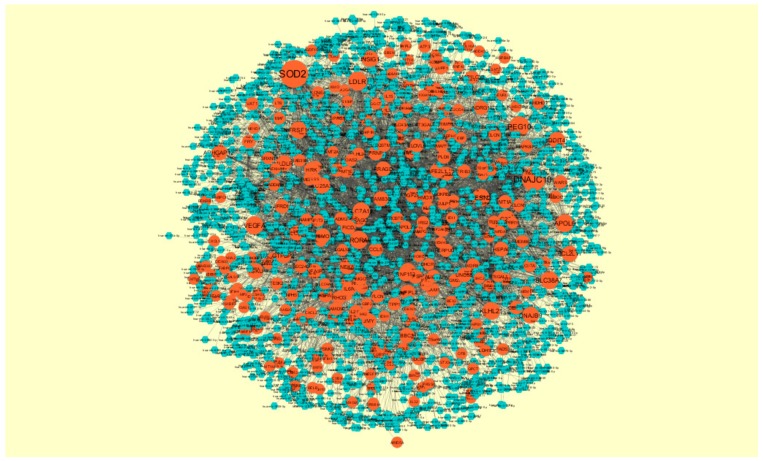
The network of down-regulated differential expressed genes (DEGs) and their related miRNAs. The orange-red circle nodes are the down-regulated DEGs, and blue diamond nodes are the miRNAs.

Target genes-miRNA interaction network (up-regulated) is shown in Figure 13. Hub genes such as *IGFBP5* interacts with 143 miRNAs, *RAD51* interacts with 113 miRNAs, *DSN1* interacts with 111 miRNAs, *RRM2* interacts with102 miRNAs, and *ZWINT* interacts with 98 miRNAs (Table S7). Target genes-miRNA interaction network (down-regulated) is shown in Figure 14. Hub genes such as *SOD2* interacts with 257 miRNAs, *DNAJC10* interacts with 195 miRNAs, *PEG10* interacts with 139 miRNAs, *LDLR* interacts with 123 miRNAs, and *RORA* interacts with 110 miRNAs (Table S7).

### 3.8. Construction of the Target Genes-TF Interaction Network

**Figure 15:**
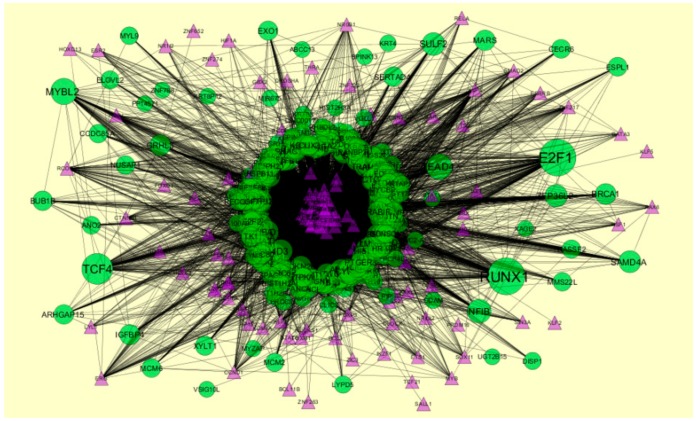
The network of up-regulated differential expressed genes (DEGs) and their related transcription factors (TFs). (Lavender triangles—TFs, and green circles—target up-regulated genes).

**Figure 16:**
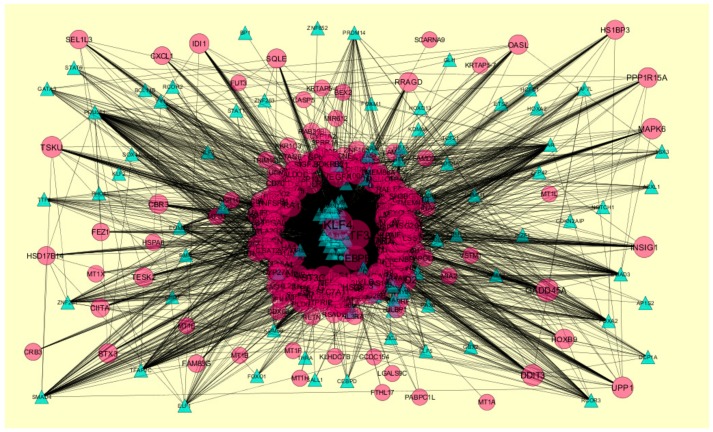
The network of down-regulated differential expressed genes (DEGs) and their related transcription factors (TFs). (Blue triangles—TFs, and pink circles—target down-regulated genes).

Target genes-TF interaction network (up-regulated) is shown in Figure 15. Hub genes such as *DHF* interacts with 178 TFs, *TBXAS1* interacts with 177 TFs, *MCEE* interacts with 154 TFs, *ETNK2* interacts with 144 TFs, and *CENPM* interacts with 137 TFs (Table S8). Target genes-TF interaction network (down-regulated) is shown in Figure 16. Hub genes such as *MT1H* interacts with 172 TFs, KRTAP5-4 interacts with 163 TFs, *RETN* interacts with 143 TFs, *HSD17B14* interacts with 132 TFs, and *SEPHS2* interacts with 124 TFs (Table S8).

### 3.9. Survival Analysis of Hub Genes

**Figure 17:**
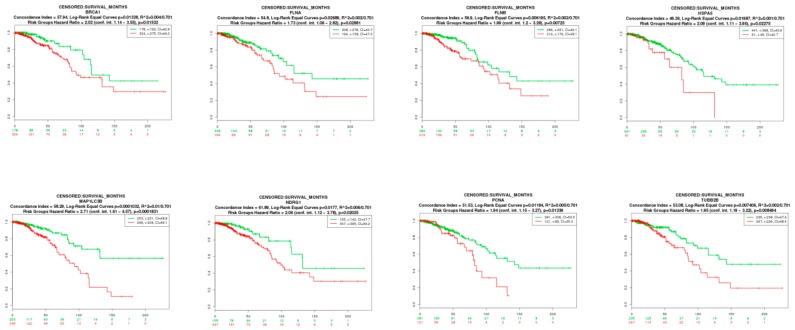
Kaplan-Meier survival curves using The Cancer Genome Atlas (TCGA) data validate the prognostic value of genes having favorable overall survival in BRCA (Green—low expression; Red—high expression).

**Figure 18:**
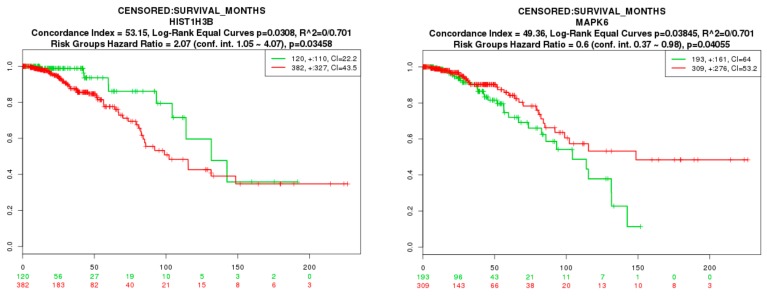
Kaplan-Meier survival curves using The Cancer Genome Atlas (TCGA) data validate the prognostic value of genes having worse overall survival in BRCA (Green—low expression; Red—high expression).

To evaluate if the identified prognostic markers are valuable in predicting patient survival, we focused on the hub genes (up- and down-regulated genes). We utilized SurvExpress [54], an online tool developed for conveniently exploring survival correlations with gene expression data from 502 cancer studies performed by The Cancer Genome Atlas (TCGA). Genes, such as *BRCA1*, *FLNA*, *FLNB*, *HSPA5*, *MAP1LC3B*, *NDRG1*, *PCNA*, and *TUBB2B*, which are overexpressed in BRCA, showed a positive correlation with patient survival. Patients with higher expression of these genes had favorable overall survival (*p*-value < 0.05) (Figure 17). Genes, such as *HIST1H3B* and *MAPK6*, which are overexpressed in BRCA, showed a negative correlation with patient survival. Patients with higher expression of these genes had worse overall survival (*p*-value < 0.05) (Figure 18).

### 3.10. Validation of Hub Genes

**Figure 19:**
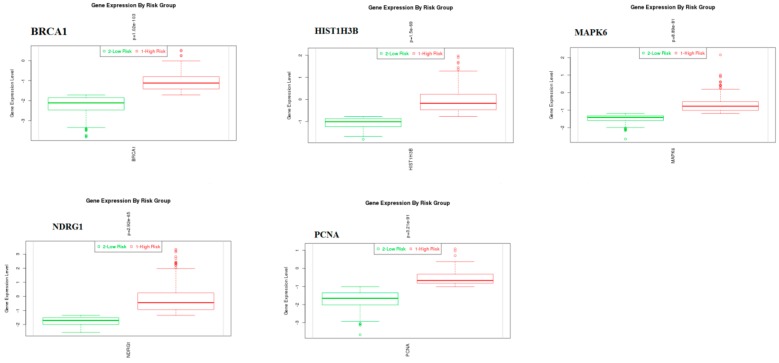
Box plots of hub genes (*BRCA1*, *HIST1H3B*, *MAPK6*, *NDRG1*, and *PCNA*). Red—high-risk; Green—low-risk.

**Figure 20:**
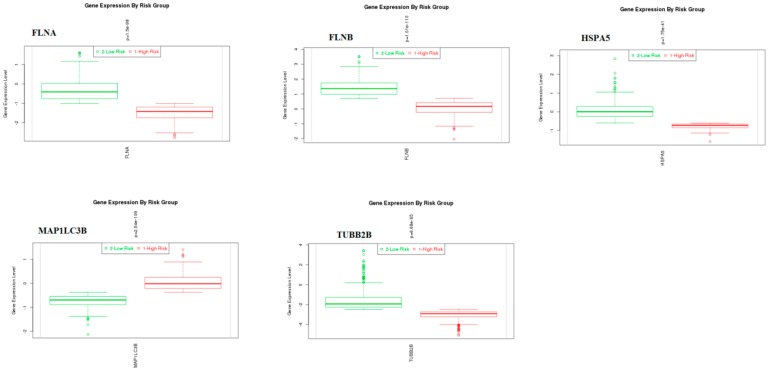
Box plots of hub genes (*FLNA*, *FLNB*, *HSPA5*, *MAP1LC3B*, and *TUBB2B*). Red—high-risk; Green—low-risk.

**Figure 21:**
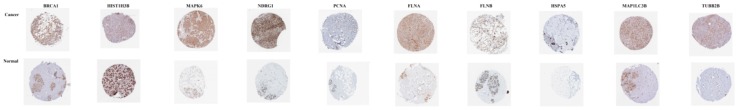
Validation of the hub genes using the Human Protein Atlas (HPA) database.

The expression level of hub genes was assessed in 2 low-risk and1 high-risk groups. The data showed that the hub gene expression of *BRCA1*, *HIST1H3B*, *MAPK6*, *NDRG1*, and *PCNA* were increased (Figure 19), while that of *FLNA*, *FLNB*, *HSPA5*, *MAP1LC3B*, and *TUBB2B* were reduced (Figure 20) in the high-risk group compared with those in the low-risk group. The outcome of the validation of the hub genes on a translational level through the HPA database are displayed in Figure 21.

### 3.11. Mutation Analysis of Hub Genes

**Figure 22:**
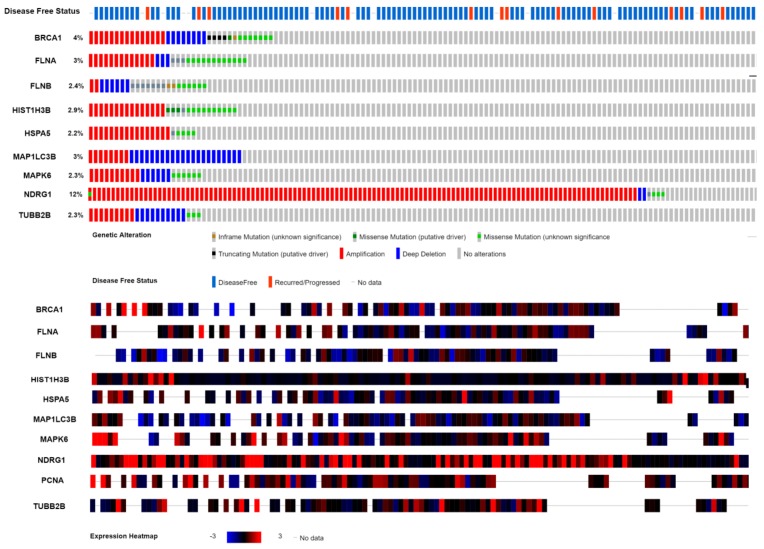
A visual summary, which displays genetic alteration of the ten hub genes in The Cancer Genome Atlas-Breast cancer (TCGA-BRCA) patients.

The mutation analysis results made the ten hub genes we screened out reliable. As for genetic mutation, ten hub genes were altered in 98.6% of 1093 patients. Figure 22 depicts the alteration information of the ten hub genes. *BRCA1*, *FLNA*, *FLNB*, *HIST1H3B*, *HSPA5*, *MAP1LC3B*, *MAPK6*, *NDRG1*, *PCNA*, and *TUBB2B* were altered most often (4%, 3%, 2.4%, 2.9%, 2.2%, 3%, 2.3%, 12%, 1.1%, and 2.3%, respectively), and these include inframe mutation, missense mutation, truncating mutation, amplification, and deep deletion.

## 4. Discussion

Breast cancer is one of the most common cancer to affect women. BRCA is a heterogeneous disease presenting distinct subtypes (Triple Negative, Luminal A, Luminal B, human epidermal growth factor receptor (HER2+)). Increased estradiol level is associated with breast cancer development through regulation of the progesterone receptor [56,57]. Estradiol antagonist tamoxifen has been the first line treatment for all stages of estrogen-receptor-positive BRCA [55]. In most cases, somatic mutations in breast cells acquired during a person’s lifetime lead to breast cancer [58]. BRCA occurs due to the accumulation of different genetic mutations, thus, a high level of molecular heterogeneity in BRCA demands thorough investigation of the molecular markers and signaling pathways associated with pathogenesis of BRCA; this may be of benefit for the examination of targeted molecular therapy to assist early diagnosis and prognosis, and may also afford a molecular basis for treatment. In the current study, the integrated analysis was performed on the gene expression profiles in estradiol- and tamoxifen-treated BRCA cell lines. Using the microarray platforms, we identified 856 DEGs (421 up-regulated and 435 down-regulated). BRCA arises from the accumulation of different gene modifications, and it is important to characterize the genetic changes during the advancement of BRCA [59]. Methylation inactivation of tumor suppressor *KCNB1* is responsible for the development of gliomas [60], but this gene may be identified with the development of BRCA. *COL12A1* is diagnosed with the pathogenesis of gastric cancer [61], but this gene may be associated with the pathogenesis of BRCA. *DIAPH3* is important for metastasis of hepatocellular carcinoma cells through stimulation of the beta-catenin/TCF signaling pathway [62], but this gene may be linked with metastasis of BRCA. *SFXN2* is important for the invasion of oral squamous cell carcinoma [63], but this gene may be responsible for the invasion of BRCA cells. *GLDC* is involved in the pathogenesis of non-small cell lung cancer cell proliferation through pyrimidine metabolism [64], but this gene may be linked with changes in amino acid and nucleic acid metabolism in BRCA. *DDIT4* is liable for the proliferation of gastric cancer cell through activation of p53 and MAPK pathways [65], but this gene may be associated with the proliferation of BRCA cells. Loss of genes, such as *INSIG1* and *ACSS2*, is responsible for the advancement of gastric cancer [66,67], but inactivation of these genes may be linked with the development of BRCA. *IFIT3* is responsible for inflammatory stimulus in pancreatic cancer [68], but this gene may be associated with inflammation in BRCA. Methylation inactivation of tumor suppressor genes, such as *FLCN* [69] and *DDIT3* [70], is important for the development of many cancer, such as renal cancer and gastric cancer, but inactivation of these genes may be responsible for the advancement of BRCA. *PRSS8* is liable for the development of ovarian cancer [71], but this gene may identify with the pathogenesis of BRCA. Genes, such as *KLF8* [72], *TCF4* [73], *H19* [74], *NEU1* [75], *CXCL1* [76], *TRIB3* [77], *FTL* [78], and *UBE2L6* [79], are responsible for the pathogenesis of BRCA.

In pathway enrichment analysis, lysine degradation II (pipecolate pathway), DNA replication, E2F transcription factor network, cell cycle, pyrimidine metabolism, CDK regulation of DNA replication, mismatch repair pathway, and pyrimidine metabolism are the most significant pathways for up-regulated genes. *CRYM* is responsible for the development of prostate cancer [80], but this gene may be identified with the pathogenesis of BRCA. Single nucleotide polymorphisms (SNP) in genes, such as *ALDH7A1* [81], *POLA2* [82], *LIG1* [83], and *ERCC6L* [84], are important for the development of various cancers, such as esophageal squamous cell carcinoma, lung cancer, and oral cancer, but these polymorphic genes may be linked with pathogenesis of BRCA. Genes, such as *MCM3* [85], *MCM5* [86], *MCM7* [87], and *DSN1* [88], are associated with the pathogenesis of various cancers, such as salivary gland cancer, cervical cancer, and hepatocellular carcinoma, through regulation of cell cycle, but these genes may be involved in progression of BRCA. Mutations in genes, such as *POLD1* [89], *CDKN2C* [90], and *HIST1H3B* [91], are involved in the pathogenesis of various cancers, such as colorectal cancer, melanoma, and gliomas, but a mutation in these genes may be important for the progression of BRCA. Genes, such as *POLE2* [92], *RFC5* [93], *MYBL2* [94], *SPC25* [95], *KIF23* [96], *NCAPG* [97], *CENPU* [98], and *ESCO2* [99], are linked with the proliferation of various cancer cells, such as lung cancer, cervical cancer, hepatocellular carcinoma, bladder cancer, and gastric cancer, but these genes may be responsible for the proliferation of BRCA cells. Genes, such as *ORC6* [100] and *GTSE1* [101], are linked with drug resistance in various cancers, such as colon cancer and gastric cancer, but these genes may be liable for drug resistance in BRCA. Genes, such as *SPC24* [102] and *PKMYT1* [103], are linked with the invasion of hepatocellular carcinoma cells, but these genes may be important for the invasion of BRCA cells. *AJUBA* is diagnosed with the growth of colorectal cancer through apoptosis inhibition [104], but this gene may be responsible for the advancement of BRCA, through inhibition of apoptosis. Genes, such as *MCM2* [105], *PCNA* [106], *RFC3* [107], *RRM2* [108], *TYMS* [109], *BRCA1* [110], *DHFR* [111], *RBBP8* [112], *E2F1* [113], *CCNA2* [114], *CCNE1* [115], *TK1* [116], *CCNE2* [117], *CDC25A* [118], *CDK2* [119], *HJURP* [120], *CDC7* [121], *NDC80* [122], *PSMC3IP* [123], *GINS2* [124], *ESPL1* [125], *BARD1* [126], *BLM* [127], *BUB1B* [128], *CDT1* [129], *RAD51* [130], *KIF20A* [131], *EXO1* [132], *AURKB* [133], *MCM10* [134], *CDCA5* [135], *LMNB1* [136], and *MSH6* [137], are responsible for the pathogenesis of BRCA. *MCM6*, *POLA1*, *RFC2*, *MND1*, *HIST2H3A*, *SYCE2*, *CDC45*, *HAUS8*, *HIST1H2BF*, *FBXL18*, *HIST1H4D*, *CENPM*, *GINS3*, *RMI2*, *GINS4*, *NDC1*, *KNTC1*, *GINS1*, *FBXO5*, *HIST1H3F*, *ZWINT*, and *CTPS1* are identified as novel molecular markers for the pathogenesis of BRCA in these pathways. While superpathway of cholesterol biosynthesis, mineral absorption, ATF-2 transcription factor network, cholesterol biosynthesis, sterol biosynthesis, genes encoding secreted soluble factors, and steroid biosynthesis are the most significant pathways for down-regulated genes. Genes, such as *HMGCS1* and *HMGCR*, are associated with the proliferation of prostate cancer cell [138], but these genes may be responsible for the proliferation of BRCA cells. Genes, such as *HMOX1* [139] and *VEGFB* [140], are important for the invasion of various cancer cells, such as bladder cancer and colorectal cancer, but these genes may be associated with the invasion of BRCA cells. Methylation inactivation in tumor suppressors genes, such as *MT1M* [141], *MT1H* [142], *MT1X* [143], and *HRK* [144], is responsible for the development of various cancers, such as prostate cancer, liver cancer, colorectal cancer, and gastric cancer, but loss of these genes may be linked with the pathogenesis of BRCA. *FGF13* is responsible for chemoresistance in cervical cancer [145], but this gene may be associated with drug resistance in BRCA. *CCL20* is linked with the development of gastric cancer [146], but this gene may be liable for the progression of BRCA. SNP in tumor suppressor genes, such as *TNFSF10* [147] and *TNFSF9* [148], are identified with the pathogenesis of various cancers, such as ovarian cancer and hepatocellular carcinoma, but these polymorphic genes may be answerable for the development of BRCA. Genes, such as *SQLE* [149], *EBP* [150], *STEAP1* [151], *MT1E* [152], *MT1F* [153], *MT2A* [154], *PLAU* [155], *ATF3* [156], *GADD45A* [157], *PPARGC1A* [158], *S100A14* [159], *S100P* [160], *CCL5* [161], *VEGFA* [162], *GDF15* [163], *IL-15* [164], *CXCL2* [165], *CXCL3* [166], *LTB* [167], and *S100A3* [168], are responsible for the pathogenesis of BRCA. *MSMO1*, *FDPS*, *IDI1*, *MVD*, *CYP51A1*, *DHCR7*, *LSS*, *BRCA*, *MT1A*, *MT1B*, *IL23A*, and *INHBE* are identified as novel molecular markers for the pathogenesis of BRCA in these pathways.

In GO enrichment analysis, cell cycle, chromosome, and DNA helicase activity are the most significant GO terms for up-regulated genes. Genes, such as *CHAF1B* [169], *INHBA* [170], *TGFB2* [171], *SKA3* [172], and *HELLS* [173], are responsible for the invasion of various cancer cells, such as hepatocellular carcinoma, gastric cancer cells, renal cell carcinoma, prostate cancer, head and neck cancer, but these genes may be involved in the invasion of BRCA cells. *TRIP13* is responsible for the development of chemoresistance in head and neck cancer [174], but this gene may be linked with the drug resistance in BRCA. *DSCC1* is associated with the development of colorectal cancer through inhibition of apoptosis [175], but this gene may be responsible for the inhibition of apoptosis in BRCA. SNP in tumor suppressor RAD54L is important for the development of pancreatic cancer [176], but this polymorphic gene may be identified with the development of BRCA. Genes, such as *CDCA3* [177], *KIF15* [178], and *TCF19* [179], are linked with the proliferation of various cancer cells, such as oral cancer, pancreatic cancer, and hepatocellular carcinoma cells, but these genes may be responsible for the proliferation of BRCA cells. Genes, such as *BOP1* [180], *KIF11* [181], and *MMS22L* [182], are associated with the progression of various cancers, such as colorectal cancer, gastric cancer, lung, and esophageal cancer, but these genes may be linked with the development of BRCA. Genes, such as *PDGFB* [183], *ANLN* [184], *RECQL4* [185], *MKI67* [186], *FGFR2* [187], *FLNA* [188], *DTL* [189], *ID3* [190], *XRCC3* [191], *SIPA1* [192], *SPAG5* [193], *EGFL6* [194], *UHRF1* [195], *CTGF* [196], *STARD13* [197], *RASSF2* [198], *PBK* [199], *NEDD9* [200], *KIFC1* [201], *E2F8* [202], *THBS1* [203], *FANCI* [204], *NUSAP1* [205], *SATB1* [206], *ASF1B* [207], and *KDM4B* [208], are responsible for the pathogenesis of BRCA. *FANCA*, *AUNIP*, *ASPM*, *DCLRE1B*, *PCLAF*, *CIT*, *HIST1H2AI*, *HIST1H2AL*, *ANKRD2*, *TONSL*, *WDHD1*, and *HIST1H1D* are identified as novel molecular markers for the pathogenesis of BRCA in these GO categories. 

While the response to cytokine, endoplasmic reticulum, and cytokine activity are the most significant GO terms for down-regulated genes. Genes, such as *SERPINA3* [209], *BIRC3* [210], and *CREBRF* [211], are liable for the proliferation of various cancer cells, such as endometrial cancer and gastric cancer, but these genes may be linked with the proliferation of BRCA cells. Genes, such as *IFIT2* [212], *CCR10* [213], and *TMEM97* [214], are important for the invasion of various cancer cells, such as oral cancer, melanoma, and glioma, but these genes may be responsible for the invasion of BRCA cells. Decreased expression of genes, such as *TNFRSF10B* [210], *MIA2* [215], and *BBC3* [216], are answerable for the progression of various cancers, such as lung cancer, hepatocellular carcinoma, head, and neck cancer, but low expression of these genes may be responsible for the development of BRCA. Genes, such as *OAS2* [217], *ULBP1* [218], *HERPUD1* [219], and *CASP4* [220], are diagnosed with the growth of various cancers, such as oral cancer, cervical cancer, and gliomas, but these genes may identify with the development of BRCA. SNP in genes, such as *PPP1R15A* [221], *PLA2G4C* [222], *CMTM8* [223], and *IFNL3* [224], are responsible for the growth of various cancers, such as colorectal cancer, osteosarcoma, and hepatocellular carcinoma, but SNP in these genes may be associated with the pathogenesis of BRCA. Mutation in *UVRAG* [225] and *RNF43* [226] is liable for the advancement of various cancers, such as gastric cancer, colorectal, and endometrial cancers, but variation in these genes may be linked with the advancement of BRCA. Methylation inactivation of tumor suppressor *ST6GAL1* [227] is responsible for the pathogenesis of bladder cancer, but the loss of this gene may be associated with the development of BRCA. Genes, such as *CEBPB* [228], *ACP5* [229], *KLF4* [230], *ACSL1* [231], *IRF9* [232], *HSPA5* [233], *ICAM1* [234], *IFITM1* [235], *IFIT1* [236], *ISG15* [237], *LAMP3* [238], *IL6R* [239], *GBP2* [240], *IRF1* [241], *CEACAM1* [242], *CD70* [243], *RORA* [244], *TNFAIP3* [245], *CIITA* [246], *SLC7A11* [247], *F7* [248], *ELOVL6* [249], *EIF2AK3* [250], *SDCBP* [251], *HYOU1* [252], *PRNP* [253], *CYP1A2* [254], *SQSTM1* [255], *NUCB2* [256], *IL32* [257], *IL15* [258], and *NAMPT* [259], are responsible for the pathogenesis of BRCA. Elevated levels of *SQSTM1* have been demonstrated in oncogenesis and resistance to cancer chemotherapy. *SQSTM1* regulates autophagy and apoptosis and acts as a signaling hub, which regulates cell viability in response to cytotoxic stress, thus playing a vital role in cancer. *SQSTM1* is a key component and player in VANGL2–JNK signaling pathway and this signaling pathway is associated with the proliferation of breast cancer cells [260]. Pleiotropic cytokine TNFα is associated with tumor cell growth, invasion, and metastasis. TNFα plays a vital role in the progression of triple negative breast cancer (TNBC) via up-regulation of *TNFAIP3*. Pleiotropic DNA damage response protein, such as BRCA1, operates in both checkpoint activation and DNA repair. In our study, survival analysis revealed that high expression of *BRCA1* was linked with breast cancer.

*PDE2A*, *IFI30*, *VLDLR*, *RSAD2*, *IL21R*, *OASL*, *IL3RA*, *MX1*, *CYBA*, *ISG20*, *SLC27A1*, *OAS1*, *RELB*, *CLGN*, *DNAJB9*, *DNAJC10*, *SDR16C5*, *APOL2*, *COL16A1*, *ERO1B*, *STARD4*, *ERO1A*, *RDH16*, *PLPP3*, *CERS1*, *SLC36A1*, *INSIG1*, *BDKRB1*, *LPIN1*, *SEC24D*, *NFE2L1*, *GPAT3*, *HS1BP3*, *FADS3*, *SLC33A1*, *RELB*, *VSTM1*, and *IFNL2* are identified as novel molecular markers for the pathogenesis of BRCA in these GO category.

In PPI network, hub genes (up-regulated), such as *MCM2*, *CDK2*, *HIST1H3F*, *HIST1H3B*, *TCF4*, *ASPM*, *CHST8*, *KCNB1*, *CFH*, *FLNA*, and *BRCA1*, are identified with high node degree, high betweenness, high stress, and high closeness. *CFH* is important for the development of lung cancer [261], but this gene may be associated with the progression of BRCA. Hub genes, such as *OLR1*, *CHST8*, *KLF8*, *CFH*, and *XAGE2*, are identified with the lowest clustering coefficient. *XAGE2* is identified as a novel molecular marker for the pathogenesis of BRCA. While hub genes (down-regulated), such as *HSPA5*, *MAPK6*, *MAP1LC3B*, *SQSTM1*, *SDCBP*, *ISG15*, *ICAM1*, and *NDRG1*, are identified with high node degree, high betweenness, high stress, and high closeness. Genes, such as *MAP1LC3B* [262], *MAPK6* [263], and *NDRG1* [264], are responsible for the development of BRCA. Hub genes, such as *NEU1*, *BRI3*, *SLC27A1*, *DDIT4*, and *CXCL1*, are identified with the lowest clustering coefficient. *DDIT4* and *BRI3* are identified as novel molecular markers for the pathogenesis of BRCA.

In module analysis, hub genes(up-regulated), such as *HIST1H1B*, *E2F1*, *POLD1*, *CDT1*, *MCM3*, *MYBL2*, *CDK2*, *CCNE1*, *CDC25A*, *PCNA*, *RAD51*, *BLM*, *MSH6*, *BARD1*, *BRCA1*, *POLA1*, *RFC3*, *RFC5*, *RFC2*, *DSCC1*, *MCM10*, *CDC7*, *MCM4*, *MCM2*, *MCM6*, and *LMNB1*, are identified in all four modules. *HIST1H1B* is identified as a novel molecular marker for the pathogenesis of BRCA. *MCM4* is associated with the pathogenesis of BRCA [265]. Meanwhile, hub genes (down-regulated), such as *HSPA5*, *HSPA6*, *MAP1LC3B*, *RELB*, *ISG15*, *IFIT1*, *IFIT2* and *IFIT3*, *TNFRSF10B*, *TNFSF10*, *TNFAIP3*, *BIRC3*, *GBP2*, *SAT1*, and *MVD*, are identified in all four modules. *HSPA6*, *MAFF*, *MAFG*, and *SAT1* are identified as novel molecular markers for the pathogenesis of BRCA.

In target genes-miRNA network, target genes (up-regulated), such as *IGFBP5*, *RAD51*, *DSN1*, *RRM2*, and *ZWINT*, are identified with a high degree. Expression of *IGFBP5* is responsible for the development of BRCA [266]. Meanwhile, target genes (down-regulated), such as *SOD2*, *DNAJC10*, *PEG10*, *LDLR*, and *RORA*, are identified with a high degree. Genes, such as *SOD2* [267], and *PEG10* [268], are associated with the pathogenesis of BRCA. *LDLR* is responsible for the advancement of prostate cancer cells [269], but this gene may be associated with the development of BRCA.

In target genes-TF network (up-regulated), target genes, such as *DHFR*, *TBXAS1*, *MCEE*, *ETNK2*, and *CENPM*, are identified with a high degree. *TBXAS1* is responsible for the development of BRCA [270]. *MCEE* and *ETNK2* are identified as novel molecular markers for the pathogenesis of BRCA. Meanwhile, target genes (down-regulated), such as *MT1H*, *KRTAP5-4*, *RETN*, *HSD17B14*, and *SEPHS2*, are identified with a high degree. *KRTAP5-4* and *HSD17B14* are identified as novel molecular markers for the pathogenesis of BRCA.

Survival analysis revealed that genes, such as *BRCA1*, *FLNA*, *FLNB*, *HSPA5*, *MAP1LC3B*, *NDRG1*, *PCNA*, and *TUBB2B*, are predicting longer survival of BRCA, while genes, such as *HIST1H3B* and *MAPK6*, are predicting shorter survival of BRCA. High expression of genes, such as *BRCA1*, *HIST1H3B*, *MAPK6*, *NDRG1*, and *PCNA*, is linked with BRCA; while low expression of genes, such as *FLNA*, *FLNB*, *HSPA5*, *MAP1LC3B*, and *TUBB2B*, are linked with BRCA. 

## 5. Conclusions

In this study, key genes were identified for the first time in estradiol and tamoxifen drug-treated BRCA by integrated bioinformatics analysis. By analyzing the pathway and GO enrichment analysis, we found that DEGs were mainly enriched in the lysine degradation II (pipecolate pathway), cholesterol biosynthesis, cell cycle, and response to cytokine, which provide a theoretical basis for studying the biological processes of BRCA. We successfully constructed a PPI network, miRNA-target gene regulatory network, and TF-target gene regulatory network of DEGs in BRCA and screened several key genes encoding proteins in the networks that are associated in the process of BRCA in the form of molecular populations. These findings promote our understanding of the molecular pathogenesis of BRCA during estradiol and tamoxifen drug treatment and may provide an enhanced perceptive of the molecular mechanisms that underlie breast cancer. However, further molecular biological experiments are required to confirm the action of the diagnosed genes that are linked with BRCA during estradiol and tamoxifen drug treatment.

## Data Availability

The datasets supporting the conclusions of this article are available in the Array Express (https://www.ebi.ac.uk/arrayexpress/) repository. [(E-MTAB-4975) (https://www.ebi.ac.uk/arrayexpress/experiments/E-MTAB-4975/)].

## Supplementary Materials

The following are available online at https://www.mdpi.com/2218-273X/9/7/282/s1, Table S1: The statistical metrics for key differentially expressed genes (DEGs), Table S2: The enriched pathway terms of the up-regulated differentially expressed genes, Table S3: The enriched pathway terms of the down-regulated differentially expressed genes, Table S4: The enriched GO terms of the up-regulated differentially expressed genes, Table S5: The enriched GO terms of the down-regulated differentially expressed genes, Table S6: Topology table for up- and down-regulated genes, Table S7: miRNA-target gene interaction table, Table S8: TF-target gene interaction table.
