# Supplementary material for: Exploring the Molecular Mechanism of the Drug-Treated Breast Cancer Based on Gene Expression Microarray
Source: Biomolecules. 2019 Jul 15;9(7):282. doi: 10.3390/biom9070282 (PMC6681318; doi:10.3390/biom9070282)
Supplement: Supplementary file 1 [file biomolecules-09-00282-s001.pdf]

Table S1. The statistical metrics for key differentially expressed genes (DEGs)

Agilent Id	Gene Symbol	logFC	pValue	FDR	tvalue	Regulation	Gene Name
A_24_P124624	OLR1	2.458429	1.19E-13	7.25E-10	24.04241	Up	oxidized low density lipoprotein receptor 1
A_23_P90273	CHST8	2.622464	3.85E-12	6.96E-09	19.05867	Up	carbohydrate sulfotransferase 8
A_23_P217528	KLF8	2.109007	4.85E-12	7.64E-09	18.76234	Up	Kruppel like factor 8
A_23_P114740	CFH	2.651636	1.85E-11	1.79E-08	17.13652	Up	complement factor H
A_23_P34031	XAGE2	2.000935	2.04E-11	1.81E-08	17.02457	Up	X antigen family member 2
A_23_P27332	TCF4	1.613097	2.32E-11	1.87E-08	16.87275	Up	transcription factor 4
A_23_P250385	HIST1H1B	2.298658	2.47E-11	1.87E-08	16.80362	Up	histone cluster 1 H1 family member b
A_33_P3288159	ASPM	2.162032	2.79E-11	2.01E-08	16.66292	Up	abnormal spindle microtubule assembly
A_24_P52697	H19	1.499364	4.09E-11	2.76E-08	16.23387	Up	H19, imprinted maternally expressed transcript (non-protein coding)
A_24_P31627	KCNB1	2.289689	6.65E-11	3.97E-08	15.70253	Up	potassium voltage-gated channel subfamily B member 1
A_23_P214168	COL12A1	2.155835	7.59E-11	4.15E-08	15.56005	Up	collagen type XII alpha 1 chain
A_33_P3271341	LOC388282	2.859496	7.61E-11	4.15E-08	15.55704	Up	uncharacterized LOC388282
A_32_P150891	DIAPH3	2.2068	7.83E-11	4.22E-08	15.5268	Up	diaphanous related formin 3
A_23_P11025	ZNF185	1.385721	8.74E-11	4.59E-08	15.41041	Up	zinc finger protein 185 with LIM domain
A_23_P96872	HSPB11	1.887166	8.94E-11	4.64E-08	15.38599	Up	heat shock protein family B (small) member 11
A_23_P107454	KRTAP3-1	2.659331	9.73E-11	4.99E-08	15.29696	Up	keratin associated protein 3-1
A_23_P30799	HIST1H3F	1.237698	1.46E-10	6.76E-08	14.87414	Up	histone cluster 1 H3 family member f
A_33_P3423874	MYZAP	1.841152	1.67E-10	7.54E-08	14.74008	Up	myocardial zonulaadherens protein
A_23_P57379	CDC45	2.380377	1.81E-10	8.04E-08	14.65398	Up	cell division cycle 45
A_23_P1102	ACTA1	1.733325	1.88E-10	8.16E-08	14.61718	Up	actin, alpha 1, skeletal muscle
A_24_P175612	SFXN2	2.898448	2.19E-10	8.87E-08	14.46427	Up	sideroflexin 2
A_24_P510377	KLF17	1.338253	2.43E-10	9.69E-08	14.35816	Up	Kruppel like factor 17
A_23_P123596	GLDC	2.316634	2.44E-10	9.69E-08	14.35701	Up	glycine decarboxylase
A_24_P142118	THBS1	1.757893	2.56E-10	9.98E-08	14.3091	Up	thrombospondin 1
A_23_P52017	ASPM	2.066532	2.76E-10	1.05E-07	14.23266	Up	abnormal spindle microtubule assembly
A_23_P84118	CDH18	2.005136	3.08E-10	1.11E-07	14.12388	Up	cadherin 18
A_23_P162719	DIAPH3	1.966629	3.11E-10	1.11E-07	14.11464	Up	diaphanous related formin 3
A_23_P57667	PLXNA1	1.540542	3.16E-10	1.12E-07	14.0991	Up	plexin A1
A_24_P921366	CALD1	1.72352	3.28E-10	1.14E-07	14.06438	Up	caldesmon 1
A_23_P2674	KRT4	1.888885	4.41E-10	1.36E-07	13.77569	Up	keratin 4
A_33_P3258117	HELLS	1.788646	5.99E-10	1.77E-07	13.48491	Up	helicase, lymphoid-specific
A_33_P3258612	PCNA	2.038069	6.52E-10	1.8E-07	13.40468	Up	proliferating cell nuclear antigen
A_23_P201319	DISP1	1.435533	6.74E-10	1.83E-07	13.37379	Up	dispatched RND transporter family member 1
A_33_P3247022	CCNE2	2.453096	7.5E-10	1.97E-07	13.27493	Up	cyclin E2
A_33_P3324206	HR	1.741985	7.59E-10	1.98E-07	13.26332	Up	HR, lysine demethylase and nuclear receptor corepressor
A_23_P93258	HIST1H3B	1.6372	9.42E-10	2.34E-07	13.0641	Up	histone cluster 1 H3 family member b
A_23_P154605	SULF2	2.267759	1.48E-09	3.33E-07	12.65466	Up	sulfatase 2
A_23_P101246	VSIG10L	1.543417	1.79E-09	3.81E-07	12.48592	Up	V-set and immunoglobulin domain containing 10 like
A_23_P23303	EXO1	2.319058	1.8E-09	3.81E-07	12.48125	Up	exonuclease 1
A_23_P212696	FSTL1	1.879818	1.84E-09	3.86E-07	12.45954	Up	follistatin like 1
A_23_P149259	TMEM79	1.195014	1.86E-09	3.88E-07	12.45131	Up	transmembrane protein 79
A_33_P3397658	SYNPO	1.524983	2.01E-09	4.12E-07	12.38209	Up	synaptopodin
A_33_P3257678	HIST2H3A	1.978938	2.08E-09	4.24E-07	12.35192	Up	histone cluster 2 H3 family member a
A_23_P63789	ZWINT	1.803883	2.24E-09	4.43E-07	12.28735	Up	ZW10 interacting kinetochore

							protein
A_23_P73982	NDC1	1.307454	2.25E-09	4.43E-07	12.28592	Up	NDC1 transmembranenucleoporin
A_23_P28886	PCNA	1.937437	2.44E-09	4.71E-07	12.21542	Up	proliferating cell nuclear antigen
A_33_P3229122	HIST1H2BF	1.53749	2.58E-09	4.9E-07	12.16609	Up	histone cluster 1 H2B family member f
A_23_P98898	CDK2	1.302806	2.61E-09	4.94E-07	12.15452	Up	cyclin dependent kinase 2
A_23_P254733	CENPU	1.816732	2.75E-09	5.13E-07	12.10978	Up	centromere protein U
A_23_P24784	TNNI2	2.797186	2.77E-09	5.15E-07	12.10274	Up	troponin I2, fast skeletal type
A_23_P389391	SLC12A4	1.47584	2.85E-09	5.25E-07	12.08008	Up	solute carrier family 12 member 4
A_33_P3379454	UHRF1	2.33689	2.93E-09	5.37E-07	12.05605	Up	ubiquitin like with PHD and ring finger domains 1
A_33_P3217238	ATAD2	1.972263	2.96E-09	5.41E-07	12.04596	Up	ATPase family, AAA domain containing 2
A_33_P3311755	KIF23	1.339783	3.15E-09	5.62E-07	11.99499	Up	kinesin family member 23
A_33_P3242124	HAUS8	1.225191	3.21E-09	5.64E-07	11.97745	Up	HAUS augmin like complex subunit 8
A_23_P88069	LHFP	2.366687	3.39E-09	5.84E-07	11.93125	Up	lipoma HMGIC fusion partner
A_23_P50096	TYMS	2.137102	3.5E-09	5.98E-07	11.90431	Up	thymidylatesynthetase
A_33_P3867534	MCM10	2.441829	3.58E-09	6.07E-07	11.88448	Up	minichromosome maintenance 10 replication initiation factor
A_23_P500433	CARD9	1.439458	3.66E-09	6.16E-07	11.86531	Up	caspase recruitment domain family member 9
A_32_P141238	ANO2	1.472287	3.76E-09	6.3E-07	11.84341	Up	anoctamin 2
A_19_P00323082	MIR675	1.231863	3.89E-09	6.43E-07	11.81377	Up	microRNA 675
A_23_P46429	CYR61	2.388957	3.9E-09	6.43E-07	11.81286	Up	cysteine rich angiogenic inducer 61
A_33_P3360216	HIST1H2AI	1.218319	3.98E-09	6.51E-07	11.79545	Up	histone cluster 1 H2A family member i
A_23_P395374	HIST1H4D	1.591705	4.03E-09	6.56E-07	11.78488	Up	histone cluster 1 H4 family member d
A_24_P397107	CDC25A	2.080784	4.22E-09	6.7E-07	11.74581	Up	cell division cycle 25A
A_23_P25873	WDHD1	1.38472	4.35E-09	6.78E-07	11.72074	Up	WD repeat and HMG-box DNA binding protein 1
A_33_P3374205	MKI67	1.210694	4.42E-09	6.83E-07	11.70721	Up	marker of proliferation Ki-67
A_23_P14072	KRT8	1.548472	4.58E-09	6.97E-07	11.67627	Up	keratin 8
A_24_P323598	ESCO2	1.715828	4.59E-09	6.97E-07	11.6752	Up	establishment of sister chromatid cohesion N-acetyltransferase 2
A_23_P88731	RAD51	1.901747	4.78E-09	7.19E-07	11.64066	Up	RAD51 recombinase
A_23_P93823	RFC2	1.346879	4.95E-09	7.32E-07	11.61281	Up	replication factor C subunit 2
A_33_P3303430	KRT8P15	1.518395	5.19E-09	7.62E-07	11.57217	Up	keratin 8 pseudogene 15
A_23_P353717	RMI2	1.992916	5.27E-09	7.71E-07	11.55996	Up	RecQ mediated genome instability 2
A_23_P143190	MYBL2	2.169627	5.79E-09	8.17E-07	11.48259	Up	MYB proto-oncogene like 2
A_23_P118246	GINS2	2.501207	5.85E-09	8.22E-07	11.47288	Up	GINS complex subunit 2
A_24_P402438	TGFB2	1.942749	5.99E-09	8.35E-07	11.45435	Up	transforming growth factor beta 2
A_32_P203528	SYCE2	1.376002	6.12E-09	8.5E-07	11.437	Up	synaptonemal complex central element protein 2
A_33_P3231953	COL12A1	1.23764	6.55E-09	8.96E-07	11.38036	Up	collagen type XII alpha 1 chain
A_23_P518	VTCN1	2.041907	6.58E-09	8.96E-07	11.37638	Up	V-set domain containing T-cell activation inhibitor 1
A_23_P159237	GPR20	1.58868	6.61E-09	8.96E-07	11.3737	Up	G protein-coupled receptor 20
A_33_P3363425	FRMD3	1.562183	7.66E-09	1.01E-06	11.2533	Up	FERM domain containing 3
A_23_P166686	AMOTL2	1.40529	7.78E-09	1.03E-06	11.23995	Up	angiomotin like 2
A_33_P3217819	CCNE2	1.766915	7.79E-09	1.03E-06	11.23889	Up	cyclin E2
A_23_P201655	MYCBP	1.914982	8.14E-09	1.06E-06	11.20337	Up	MYC binding protein
A_23_P50108	NDC80	1.470187	8.16E-09	1.06E-06	11.20161	Up	NDC80, kinetochore complex component
A_23_P102202	MSH6	1.309479	8.55E-09	1.08E-06	11.16414	Up	mutS homolog 6
A_33_P3382399	KRTAP3-1	1.634798	8.56E-09	1.08E-06	11.16285	Up	keratin associated protein 3-1
A_19_P00320541	AC013652.1	2.24957	8.83E-09	1.11E-06	11.13777	Up	NA
A_23_P26557	C16orf59	1.422758	9.08E-09	1.12E-06	11.11528	Up	chromosome 16 open reading frame 59
A_23_P51085	SPC25	2.069464	9.34E-09	1.13E-06	11.09265	Up	SPC25, NDC80 kinetochore complex component

A_23_P138938	PGR	2.52088	9.7E-09	1.16E-06	11.06231	Up	progesterone receptor
A_23_P12816	HELLS	1.926318	9.91E-09	1.17E-06	11.04539	Up	helicase, lymphoid-specific
A_23_P205959	ALDH1A3	1.698803	1.02E-08	1.19E-06	11.02557	Up	aldehyde dehydrogenase 1 family member A3
A_23_P73526	CITED1	1.92837	1.03E-08	1.2E-06	11.01773	Up	Cbp/p300 interacting transactivator with Glu/Asp rich carboxy-terminal domain 1
A_23_P259741	SATB1	1.654762	1.06E-08	1.22E-06	10.98942	Up	SATB homeobox 1
A_23_P7873	MCM3	1.886593	1.18E-08	1.32E-06	10.90884	Up	minichromosome maintenance complex component 3
A_23_P353614	C8orf46	1.667174	1.19E-08	1.33E-06	10.89899	Up	chromosome 8 open reading frame 46
A_23_P165937	DSN1	1.515823	1.2E-08	1.34E-06	10.89116	Up	DSN1 homolog, MIS12 kinetochore complex component
A_24_P870799	GRIK3	1.727525	1.21E-08	1.34E-06	10.88405	Up	glutamate ionotropic receptor kainate type subunit 3
A_32_P95729	FANCI	1.290405	1.26E-08	1.38E-06	10.85348	Up	Fanconianemia complementation group I
A_23_P48835	KIF23	1.362573	1.32E-08	1.42E-06	10.8159	Up	kinesin family member 23
A_23_P207400	BRCA1	1.50827	1.34E-08	1.42E-06	10.80283	Up	BRCA1, DNA repair associated
A_23_P58407	UGT2B15	1.813974	1.38E-08	1.44E-06	10.78247	Up	UDP glucuronosyltransferase family 2 member B15
A_33_P3210363	TMPO-AS1	1.982074	1.4E-08	1.45E-06	10.76865	Up	TMPO antisense RNA 1
A_24_P225616	RRM2	2.705192	1.43E-08	1.47E-06	10.75423	Up	ribonucleotidereductase regulatory subunit M2
A_33_P3278313	MSRB3	1.430904	1.45E-08	1.48E-06	10.7428	Up	methionine sulfoxidereductase B3
A_33_P3415820	THBS1	1.392338	1.49E-08	1.52E-06	10.72289	Up	thrombospondin 1
A_33_P3221980	KRT8P51	1.680465	1.52E-08	1.54E-06	10.70521	Up	keratin 8 pseudogene 51
A_23_P202334	FGFR2	1.250968	1.58E-08	1.58E-06	10.67884	Up	fibroblast growth factor receptor 2
A_23_P206441	FANCA	1.412165	1.63E-08	1.63E-06	10.65186	Up	Fanconianemia complementation group A
A_24_P7750	#N/A	1.676093	1.74E-08	1.68E-06	10.60342	Up	NA
A_24_P303454	TIAM2	1.355437	1.77E-08	1.7E-06	10.59021	Up	T-cell lymphoma invasion and metastasis 2
A_23_P39116	LIG1	1.239615	1.78E-08	1.71E-06	10.58189	Up	DNA ligase 1
A_23_P36187	SYT8	1.736077	1.82E-08	1.73E-06	10.56565	Up	synaptotagmin 8
A_23_P349566	CCDC85A	1.901593	1.84E-08	1.74E-06	10.55857	Up	coiled-coil domain containing 85A
A_32_P49199	PGR	3.082256	1.85E-08	1.75E-06	10.55587	Up	progesterone receptor
A_23_P216307	RUNX1T1	1.399975	1.96E-08	1.82E-06	10.5098	Up	RUNX1 translocation partner 1
A_23_P146946	CST6	2.079093	2E-08	1.85E-06	10.49446	Up	cystatin E/M
A_24_P287941	PSMC3IP	1.381789	2.05E-08	1.88E-06	10.47596	Up	PSMC3 interacting protein
A_33_P3308833	C21orf58	1.27697	2.06E-08	1.88E-06	10.47052	Up	chromosome 21 open reading frame 58
A_23_P106806	PRSS27	1.615036	2.07E-08	1.88E-06	10.46894	Up	protease, serine 27
A_33_P3320272	TMEM79	1.224257	2.16E-08	1.93E-06	10.4356	Up	transmembrane protein 79
A_23_P160537	AUNIP	1.401028	2.36E-08	2.07E-06	10.36843	Up	aurora kinase A and ninein interacting protein
A_24_P175909	MARS	2.372272	2.44E-08	2.11E-06	10.34221	Up	methionyl-tRNAsynthetase
A_33_P3237379	KCNC1	1.960712	2.45E-08	2.11E-06	10.34017	Up	potassium voltage-gated channel subfamily C member 1
A_24_P358131	#N/A	1.216654	2.57E-08	2.2E-06	10.30172	Up	NA
A_23_P209200	CCNE1	1.935678	2.61E-08	2.22E-06	10.2921	Up	cyclin E1
A_23_P362893	TEAD1	1.375645	2.82E-08	2.37E-06	10.23245	Up	TEA domain transcription factor 1
A_23_P163099	POLE2	1.884469	2.88E-08	2.4E-06	10.21779	Up	DNA polymerase epsilon 2, accessory subunit
A_23_P133956	KIFC1	1.432381	2.92E-08	2.43E-06	10.2053	Up	kinesin family member C1
A_23_P408955	E2F2	2.838042	2.93E-08	2.43E-06	10.20398	Up	E2F transcription factor 2
A_33_P3408420	MDGA2	1.207273	2.96E-08	2.45E-06	10.19521	Up	MAM domain containing glycosylphosphatidylinositol anchor 2
A_23_P425502	DONSON	1.250384	2.97E-08	2.46E-06	10.19227	Up	downstream neighbor of SON
A_33_P3230254	NCAPG	1.627985	3E-08	2.47E-06	10.18497	Up	non-SMC condensin I complex subunit G
A_23_P141965	HAUS8	1.253082	3.09E-08	2.53E-06	10.16411	Up	HAUS augmin like complex subunit 8

A_23_P132277	MCM5	2.571932	3.1E-08	2.53E-06	10.16186	Up	minichromosome maintenance complex component 5
A_24_P382187	IGFBP4	1.669598	3.18E-08	2.56E-06	10.14296	Up	insulin like growth factor binding protein 4
A_33_P3351279	GPR37L1	1.537334	3.18E-08	2.56E-06	10.14288	Up	G protein-coupled receptor 37 like 1
A_19_P00316362	#N/A	1.193212	3.18E-08	2.56E-06	10.14206	Up	NA
A_23_P54055	AJUBA	1.391776	3.2E-08	2.57E-06	10.13757	Up	ajuba LIM protein
A_33_P3339212	TRIP13	1.633646	3.26E-08	2.6E-06	10.12243	Up	thyroid hormone receptor interactor 13
A_23_P133755	PREP	1.215218	3.3E-08	2.62E-06	10.11518	Up	prolylendopeptidase
A_33_P3417589	KRT8P44	1.578873	3.31E-08	2.63E-06	10.11187	Up	keratin 8 pseudogene 44
A_19_P00323413	PTPN14	1.674537	3.33E-08	2.64E-06	10.10772	Up	protein tyrosine phosphatase, non-receptor type 14
A_19_P00801752	#N/A	1.259645	3.48E-08	2.74E-06	10.07433	Up	NA
A_33_P3218649	GPM6B	1.734416	3.6E-08	2.82E-06	10.0486	Up	glycoprotein M6B
A_23_P105144	SCUBE2	1.41643	3.74E-08	2.89E-06	10.02103	Up	signal peptide, CUB domain and EGF like domain containing 2
A_33_P3236157	UNG	1.633319	3.75E-08	2.89E-06	10.01831	Up	uracil DNA glycosylase
A_32_P117464	MB21D2	1.623954	3.79E-08	2.91E-06	10.01183	Up	Mab-21 domain containing 2
A_33_P3421913	CADM1	1.407824	3.83E-08	2.93E-06	10.00429	Up	cell adhesion molecule 1
A_23_P117852	PCLAF	1.68639	3.86E-08	2.95E-06	9.997409	Up	PCNA clamp associated factor
A_23_P10385	DTL	2.544425	4.13E-08	3.11E-06	9.94693	Up	denticleless E3 ubiquitin protein ligase homolog
A_23_P136817	KNTC1	1.266284	4.15E-08	3.12E-06	9.944542	Up	kinetochore associated 1
A_23_P14193	RFC3	1.572997	4.19E-08	3.14E-06	9.936876	Up	replication factor C subunit 3
A_33_P3270657	FAM111B	3.405527	4.33E-08	3.22E-06	9.912952	Up	family with sequence similarity 111 member B
A_32_P52018	PHACTR1	1.391961	4.61E-08	3.38E-06	9.866613	Up	phosphatase and actin regulator 1
A_33_P3469658	LOC284570	1.912981	4.8E-08	3.5E-06	9.837625	Up	uncharacterized LOC284570
A_24_P349965	TCF19	2.033663	4.91E-08	3.55E-06	9.819794	Up	transcription factor 19
A_24_P383523	SAMD4A	1.274144	4.95E-08	3.56E-06	9.814347	Up	sterile alpha motif domain containing 4A
A_33_P3384871	FBXO5	1.623674	4.96E-08	3.56E-06	9.812716	Up	F-box protein 5
A_23_P76015	ARHGEF17	1.226005	5.07E-08	3.63E-06	9.796539	Up	Rho guanine nucleotide exchange factor 17
A_23_P152136	GINS3	1.408777	5.3E-08	3.77E-06	9.765157	Up	GINS complex subunit 3
A_24_P169843	#N/A	1.264863	5.39E-08	3.81E-06	9.751775	Up	NA
A_33_P3268487	ABAT	1.982372	5.42E-08	3.81E-06	9.747668	Up	4-aminobutyrate aminotransferase
A_24_P399888	CENPM	1.543883	5.48E-08	3.84E-06	9.740859	Up	centromere protein M
A_24_P69053	TENM3	1.683766	5.49E-08	3.84E-06	9.739322	Up	teneurintransmembrane protein 3
A_23_P89509	SPAG5	1.236428	5.63E-08	3.91E-06	9.720935	Up	sperm associated antigen 5
A_33_P3324814	SATB1-AS1	1.810048	5.92E-08	4.05E-06	9.684681	Up	SATB1 antisense RNA 1
A_24_P96403	RUNX1	1.427232	6.04E-08	4.12E-06	9.670166	Up	runt related transcription factor 1
A_23_P342727	STARD13	1.638256	6.06E-08	4.13E-06	9.667568	Up	StAR related lipid transfer domain containing 13
A_23_P344555	NEDD9	1.944507	6.18E-08	4.19E-06	9.653604	Up	neural precursor cell expressed, developmentally down-regulated 9
A_24_P413126	PMEPA1	1.490607	6.25E-08	4.22E-06	9.64552	Up	prostate transmembrane protein, androgen induced 1
A_23_P50455	POLD1	1.283182	6.33E-08	4.26E-06	9.635262	Up	DNA polymerase delta 1, catalytic subunit
A_23_P148807	CDC7	1.44694	6.35E-08	4.27E-06	9.633244	Up	cell division cycle 7
A_23_P370989	MCM4	1.621511	6.38E-08	4.27E-06	9.630267	Up	minichromosome maintenance complex component 4
A_33_P3340040	GINS4	1.897165	6.6E-08	4.4E-06	9.605355	Up	GINS complex subunit 4
A_33_P3212994	ZWINT	1.720094	6.93E-08	4.58E-06	9.570364	Up	ZW10 interacting kinetochore protein
A_33_P3284951	MCM5	2.347231	7.11E-08	4.67E-06	9.551748	Up	minichromosome maintenance complex component 5
A_23_P3681	NETO2	1.30966	7.21E-08	4.72E-06	9.541986	Up	neuropilin and tolloid like 2
A_23_P211878	FLNB	1.423202	7.58E-08	4.9E-06	9.506114	Up	filamin B
A_23_P64873	DCN	1.584729	7.65E-08	4.94E-06	9.500264	Up	decorin

A_23_P119254	ASF1B	1.801986	8.09E-08	5.21E-06	9.45974	Up	anti-silencing function 1B histone chaperone
A_33_P3214446	RFC3	1.451268	8.31E-08	5.32E-06	9.44073	Up	replication factor C subunit 3
A_33_P3319920	KDM4B	1.752937	8.39E-08	5.34E-06	9.434134	Up	lysine demethylase 4B
A_33_P3807062	HJURP	1.53697	8.42E-08	5.35E-06	9.431311	Up	Holliday junction recognition protein
A_23_P17811	SEC14L2	2.0725	8.46E-08	5.36E-06	9.428588	Up	SEC14 like lipid binding 2
A_23_P2543	CUX2	1.598522	8.48E-08	5.37E-06	9.426303	Up	cut like homeobox 2
A_24_P743907	DNM1L	1.211611	8.73E-08	5.49E-06	9.406132	Up	dynamin 1 like
A_23_P116249	GRIK4	1.828314	8.77E-08	5.5E-06	9.402495	Up	glutamate ionotropic receptor kainate type subunit 4
A_23_P104651	CDCA5	1.602608	9.23E-08	5.72E-06	9.366618	Up	cell division cycle associated 5
A_33_P3397443	PKMYT1	1.927508	9.24E-08	5.72E-06	9.36539	Up	protein kinase, membrane associated tyrosine/threonine 1
A_33_P3267203	LOC100131132	1.533734	9.83E-08	5.98E-06	9.322418	Up	uncharacterized LOC100131132
A_23_P203439	KCNC1	2.366261	9.86E-08	5.98E-06	9.320186	Up	potassium voltage-gated channel subfamily C member 1
A_23_P80902	KIF15	1.500483	1.01E-07	6.11E-06	9.302435	Up	kinesin family member 15
A_32_P1701	POLA1	1.329357	1.02E-07	6.12E-06	9.297919	Up	DNA polymerase alpha 1, catalytic subunit
A_23_P164814	C19orf57	1.506109	1.03E-07	6.17E-06	9.290675	Up	chromosome 19 open reading frame 57
A_33_P3350488	NUSAP1	1.253253	1.08E-07	6.4E-06	9.257845	Up	nucleolar and spindle associated protein 1
A_23_P127460	SIPA1	1.601989	1.08E-07	6.43E-06	9.254122	Up	signal-induced proliferation-associated 1
A_24_P210637	CHST14	1.690571	1.1E-07	6.48E-06	9.245412	Up	carbohydrate sulfotransferase 14
A_23_P80032	E2F1	2.553537	1.14E-07	6.67E-06	9.218726	Up	E2F transcription factor 1
A_23_P332326	ARHGEF19	1.318794	1.16E-07	6.75E-06	9.206897	Up	Rho guanine nucleotide exchange factor 19
A_23_P434809	S100A8	2.439531	1.17E-07	6.8E-06	9.199272	Up	S100 calcium binding protein A8
A_33_P3256920	WNT7B	1.381771	1.18E-07	6.8E-06	9.196732	Up	Wnt family member 7B
A_33_P3846653	KRT19P2	1.345344	1.18E-07	6.83E-06	9.193594	Up	keratin 19 pseudogene 2
A_23_P32707	ESPL1	1.571908	1.19E-07	6.86E-06	9.186845	Up	extra spindle pole bodies like 1, separase
A_23_P144778	CKMT2	1.884808	1.22E-07	6.97E-06	9.171004	Up	creatine kinase, mitochondrial 2
A_33_P3264926	SAMD4A	1.25773	1.23E-07	7.01E-06	9.166107	Up	sterile alpha motif domain containing 4A
A_33_P3280074	GRIK4	2.09421	1.25E-07	7.08E-06	9.154504	Up	glutamate ionotropic receptor kainate type subunit 4
A_23_P84154	ARHGAP15	1.562727	1.28E-07	7.24E-06	9.13585	Up	Rho GTPase activating protein 15
A_23_P32165	LHX2	1.219591	1.29E-07	7.28E-06	9.130612	Up	LIM homeobox 2
A_23_P100344	ORC6	1.267293	1.31E-07	7.36E-06	9.121925	Up	origin recognition complex subunit 6
A_23_P90612	MCM6	2.02271	1.32E-07	7.38E-06	9.117188	Up	minichromosome maintenance complex component 6
A_23_P80839	MAP6D1	3.560903	1.32E-07	7.38E-06	9.116455	Up	MAP6 domain containing 1
A_33_P3240295	KRT18P26	1.257596	1.37E-07	7.59E-06	9.091125	Up	keratin 18 pseudogene 26
A_23_P58228	ODAM	2.001659	1.38E-07	7.64E-06	9.083996	Up	odontogenic, ameloblastasssociated
A_33_P3369844	CD24	1.227449	1.45E-07	7.92E-06	9.053131	Up	CD24 molecule
A_23_P74115	RAD54L	1.67793	1.47E-07	8.03E-06	9.03988	Up	RAD54 like (S. cerevisiae)
A_24_P343095	DHFR	1.286417	1.49E-07	8.11E-06	9.032166	Up	dihydrofolatereductase
A_32_P86739	CASC10	1.61625	1.52E-07	8.24E-06	9.020189	Up	cancer susceptibility 10
A_19_P00806947	PCNAP3	2.010038	1.54E-07	8.27E-06	9.009891	Up	proliferating cell nuclear antigen pseudogene 3
A_33_P3273552	KRT83	1.282981	1.57E-07	8.39E-06	8.997369	Up	keratin 83
A_23_P23048	S100A9	1.813691	1.61E-07	8.58E-06	8.979968	Up	S100 calcium binding protein A9
A_23_P52067	GRHL3	1.765401	1.71E-07	9.02E-06	8.938598	Up	grainyhead like transcription factor 3
A_32_P62997	PBK	1.354573	1.78E-07	9.31E-06	8.911145	Up	PDZ binding kinase
A_23_P67771	BARD1	1.415919	1.79E-07	9.32E-06	8.907389	Up	BRCA1 associated RING domain 1
A_19_P00318418	TBXAS1	1.435727	1.8E-07	9.38E-06	8.901741	Up	thromboxane A synthase 1
A_23_P211428	SMTN	1.917984	1.86E-07	9.58E-06	8.880088	Up	smoothelin
A_23_P363174	HIST1H2AL	1.620374	1.86E-07	9.58E-06	8.879971	Up	histone cluster 1 H2A family

							member I
A_23_P93690	MCM7	1.209571	1.96E-07	9.96E-06	8.844714	Up	minichromosome maintenance complex component 7
A_33_P3290709	EGFL6	1.800637	2.03E-07	1.02E-05	8.822167	Up	EGF like domain multiple 6
A_24_P247454	KRT18P19	1.198426	2.08E-07	1.05E-05	8.803953	Up	keratin 18 pseudogene 19
A_24_P339944	PDGFB	1.754048	2.08E-07	1.05E-05	8.803746	Up	platelet derived growth factor subunit B
A_23_P56654	MCEE	1.253297	2.15E-07	1.07E-05	8.782962	Up	methylmalonyl-CoA epimerase
A_33_P3286754	SEC14L2	1.253641	2.2E-07	1.09E-05	8.766399	Up	SEC14 like lipid binding 2
A_33_P3252730	ZNF788	1.198008	2.21E-07	1.09E-05	8.763292	Up	zinc finger family member 788
A_33_P3381948	WTIP	1.512168	2.3E-07	1.13E-05	8.737514	Up	WT1 interacting protein
A_23_P88630	BLM	1.423384	2.38E-07	1.15E-05	8.71381	Up	Bloom syndrome RecQ like helicase
A_23_P58706	SPINK13	1.969783	2.43E-07	1.17E-05	8.701016	Up	serine peptidase inhibitor, Kazal type 13 (putative)
A_33_P3386723	KRT18P64	1.193788	2.53E-07	1.21E-05	8.674065	Up	keratin 18 pseudogene 64
A_33_P3326210	ESCO2	1.47346	2.56E-07	1.21E-05	8.66509	Up	establishment of sister chromatid cohesion N-acetyltransferase 2
A_23_P208880	UHRF1	2.976698	2.64E-07	1.24E-05	8.644357	Up	ubiquitin like with PHD and ring finger domains 1
A_33_P3323847	RECQL4	1.490364	2.68E-07	1.25E-05	8.634586	Up	RecQ like helicase 4
A_24_P317762	LY6E	1.575428	2.73E-07	1.27E-05	8.621272	Up	lymphocyte antigen 6 family member E
A_33_P3313835	PP14571	2.112958	2.75E-07	1.28E-05	8.618115	Up	uncharacterized LOC100130449
A_23_P70307	SMOC2	1.299088	2.88E-07	1.33E-05	8.585685	Up	SPARC related modular calcium binding 2
A_23_P10232	BANK1	1.273249	3E-07	1.38E-05	8.558498	Up	B-cell scaffold protein with ankyrin repeats 1
A_23_P107421	TK1	2.034346	3.09E-07	1.41E-05	8.540238	Up	thymidine kinase 1
A_23_P58321	CCNA2	1.251382	3.14E-07	1.43E-05	8.528532	Up	cyclin A2
A_32_P103633	MCM2	1.743227	3.15E-07	1.43E-05	8.526335	Up	minichromosome maintenance complex component 2
A_24_P381199	TRIM6	1.634031	3.16E-07	1.44E-05	8.524091	Up	tripartite motif containing 6
A_24_P227091	KIF11	1.430317	3.17E-07	1.44E-05	8.523057	Up	kinesin family member 11
A_23_P103486	CYP2J2	1.233798	3.26E-07	1.47E-05	8.504648	Up	cytochrome P450 family 2 subfamily J member 2
A_24_P2948	RUSC1	1.407122	3.36E-07	1.51E-05	8.483651	Up	RUN and SH3 domain containing 1
A_23_P259344	CECR6	1.287723	3.39E-07	1.52E-05	8.477778	Up	cat eye syndrome chromosome region, candidate 6
A_24_P205589	ACOT7	1.45587	3.52E-07	1.57E-05	8.453764	Up	acyl-CoA thioesterase 7
A_33_P3212860	AMTN	1.770814	3.59E-07	1.59E-05	8.441092	Up	amelotin
A_23_P131935	FERMT1	2.142431	3.85E-07	1.68E-05	8.39494	Up	fermitin family member 1
A_23_P252740	DSCC1	1.376165	4.08E-07	1.76E-05	8.356585	Up	DNA replication and sister chromatid cohesion 1
A_24_P658427	NFIB	1.309672	4.1E-07	1.76E-05	8.353309	Up	nuclear factor I B
A_32_P95914	MMS22L	1.298214	4.11E-07	1.76E-05	8.351909	Up	MMS22 like, DNA repair protein
A_24_P54131	DCLRE1B	1.27363	4.4E-07	1.86E-05	8.307357	Up	DNA cross-link repair 1B
A_23_P122924	INHBA	1.330578	4.46E-07	1.88E-05	8.299262	Up	inhibin beta A subunit
A_33_P3283601	PLAC9P1	1.448628	5E-07	2.06E-05	8.224685	Up	placenta specific 9 pseudogene 1
A_23_P130182	AURKB	1.30725	5.35E-07	2.16E-05	8.181165	Up	aurora kinase B
A_33_P3387831	CENPM	1.290515	5.68E-07	2.26E-05	8.142107	Up	centromere protein M
A_23_P161439	ADIRF	1.323988	6.3E-07	2.45E-05	8.075953	Up	adipogenesis regulatory factor
A_23_P57306	CHAF1B	1.194358	6.32E-07	2.45E-05	8.074432	Up	chromatin assembly factor 1 subunit B
A_33_P3340025	GINS1	2.119947	6.6E-07	2.54E-05	8.046275	Up	GINS complex subunit 1
A_23_P256956	KIF20A	1.230024	6.75E-07	2.59E-05	8.032685	Up	kinesin family member 20A
A_33_P3366120	FLNA	1.776102	7.11E-07	2.7E-05	7.999051	Up	filamin A
A_33_P3265739	PTGER3	1.719373	7.15E-07	2.71E-05	7.996034	Up	prostaglandin E receptor 3
A_23_P6818	SEMA3G	1.591164	7.2E-07	2.72E-05	7.99147	Up	semaphorin 3G
A_24_P260639	HIST1H1D	1.23834	7.27E-07	2.74E-05	7.984851	Up	histone cluster 1 H1 family member d
A_23_P218237	LCAT	1.303124	7.43E-07	2.78E-05	7.971441	Up	lecithin-cholesterol acyltransferase
A_23_P63432	RHBDL2	1.206997	7.53E-07	2.8E-05	7.962763	Up	rhomboid like 2
A_33_P3322400	LOC654780	1.194553	7.54E-07	2.8E-05	7.96203	Up	splicing factor proline/glutamine-rich

A_23_P116264	NRGN	1.520112	7.72E-07	2.85E-05	7.947197	Up	neurogranin
A_24_P931443	GPR68	1.881912	7.75E-07	2.85E-05	7.94439	Up	G protein-coupled receptor 68
A_19_P00321549	SATB1-AS1	1.758396	7.75E-07	2.85E-05	7.94433	Up	SATB1 antisense RNA 1
A_33_P3391316	AL050303.4	2.35552	7.76E-07	2.85E-05	7.944049	Up	NA
A_24_P346855	MKI67	1.466374	8.31E-07	3.02E-05	7.900778	Up	marker of proliferation Ki-67
A_23_P101516	KCNC3	1.322962	8.95E-07	3.21E-05	7.854167	Up	potassium voltage-gated channel subfamily C member 3
A_24_P12413	TRAM2	1.387416	9.4E-07	3.31E-05	7.823138	Up	translocation associated membrane protein 2
A_33_P3330498	ALDH7A1	1.29044	9.49E-07	3.34E-05	7.81697	Up	aldehyde dehydrogenase 7 family member A1
A_24_P354496	WWC2-AS2	1.214931	9.55E-07	3.35E-05	7.813145	Up	WWC2 antisense RNA 2
A_23_P398854	DOK7	2.300764	9.61E-07	3.37E-05	7.809699	Up	docking protein 7
A_23_P95302	RFC5	1.234412	9.77E-07	3.41E-05	7.799273	Up	replication factor C subunit 5
A_23_P47116	RASSF7	1.374718	1.05E-06	3.62E-05	7.754843	Up	Ras association domain family member 7
A_23_P356070	FLT4	1.925195	1.06E-06	3.65E-05	7.745623	Up	fms related tyrosine kinase 4
A_32_P222961	SPIN4	1.335368	1.1E-06	3.75E-05	7.72497	Up	spindlin family member 4
A_23_P21706	CTPS1	1.196289	1.15E-06	3.88E-05	7.696485	Up	CTP synthase 1
A_33_P3346669	PLCE1	1.611847	1.23E-06	4.08E-05	7.654406	Up	phospholipase C epsilon 1
A_33_P3349259	TCAF1	1.50156	1.32E-06	4.32E-05	7.614168	Up	TRPM8 channel associated factor 1
A_23_P19663	CTGF	1.232308	1.33E-06	4.35E-05	7.608171	Up	connective tissue growth factor
A_23_P420551	CIT	1.251006	1.36E-06	4.44E-05	7.593324	Up	citron rho-interacting serine/threonine kinase
A_23_P57588	GTSE1	1.457709	1.38E-06	4.49E-05	7.5841	Up	G2 and S-phase expressed 1
A_23_P201939	PPM1J	1.54581	1.42E-06	4.57E-05	7.569709	Up	protein phosphatase, Mg2+/Mn2+ dependent 1J
A_23_P69738	RASL11B	1.319823	1.47E-06	4.72E-05	7.545961	Up	RAS like family 11 member B
A_33_P3256952	EGLN3	1.338932	1.48E-06	4.74E-05	7.541611	Up	egl-9 family hypoxia inducible factor 3
A_23_P43157	MYBL1	2.513167	1.48E-06	4.74E-05	7.540994	Up	MYB proto-oncogene like 1
A_33_P3301524	XRCC3	1.345025	1.54E-06	4.87E-05	7.518068	Up	X-ray repair cross complementing 3
A_23_P101960	ZFP36L2	1.211004	1.6E-06	5.03E-05	7.49295	Up	ZFP36 ring finger protein like 2
A_23_P99292	RAD51AP1	1.3276	1.61E-06	5.04E-05	7.48977	Up	RAD51 associated protein 1
A_24_P70303	CACNG4	1.190928	1.66E-06	5.14E-05	7.472032	Up	calcium voltage-gated channel auxiliary subunit gamma 4
A_24_P247074	KRT8P37	1.671278	1.67E-06	5.15E-05	7.469519	Up	keratin 8 pseudogene 37
A_23_P403991	TRPM8	1.306607	1.71E-06	5.24E-05	7.455968	Up	transient receptor potential cation channel subfamily M member 8
A_23_P210425	MYL9	1.39683	1.77E-06	5.41E-05	7.432879	Up	myosin light chain 9
A_24_P403561	LRP4	1.472548	1.82E-06	5.51E-05	7.416582	Up	LDL receptor related protein 4
A_24_P787897	XYLT1	1.476984	1.89E-06	5.64E-05	7.395088	Up	xylosyltransferase 1
A_23_P501007	EFEMP1	1.214461	1.89E-06	5.65E-05	7.394535	Up	EGF containing fibulin like extracellular matrix protein 1
A_32_P214665	PCP4L1	1.34285	1.91E-06	5.67E-05	7.388571	Up	Purkinje cell protein 4 like 1
A_23_P137381	ID3	1.300363	1.92E-06	5.72E-05	7.383613	Up	inhibitor of DNA binding 3, HLH protein
A_23_P8702	PIP	1.672604	1.98E-06	5.85E-05	7.365358	Up	prolactin induced protein
A_23_P107981	SULT2B1	1.26063	2.1E-06	6.16E-05	7.330911	Up	sulfotransferase family 2B member 1
A_23_P216355	TONSL	1.730085	2.11E-06	6.19E-05	7.326312	Up	tonsoku like, DNA repair protein
A_23_P329768	GREB1	2.128292	2.12E-06	6.2E-05	7.325404	Up	growth regulation by estrogen in breast cancer 1
A_23_P53176	FOLR1	1.257685	2.14E-06	6.25E-05	7.318527	Up	folate receptor 1
A_33_P3386262	CDT1	1.274442	2.17E-06	6.3E-05	7.309681	Up	chromatin licensing and DNA replication factor 1
A_23_P383009	IGFBP5	2.671087	2.2E-06	6.37E-05	7.302198	Up	insulin like growth factor binding protein 5
A_19_P00323737	PTPN14	1.717658	2.21E-06	6.38E-05	7.299708	Up	protein tyrosine phosphatase, non-receptor type 14
A_23_P21990	SLC23A1	1.285596	2.22E-06	6.4E-05	7.295788	Up	solute carrier family 23 member 1
A_33_P3292540	CDKN2C	1.275652	2.23E-06	6.4E-05	7.294623	Up	cyclin dependent kinase inhibitor 2C

A_23_P144096	CISH	3.864857	2.33E-06	6.63E-05	7.266681	Up	cytokine inducible SH2 containing protein
A_23_P402765	NRAP	1.415414	2.34E-06	6.65E-05	7.26391	Up	nebulin related anchoring protein
A_23_P206018	TPM1	1.220139	2.36E-06	6.67E-05	7.260509	Up	tropomyosin 1 (alpha)
A_23_P356684	ANLN	1.278739	2.38E-06	6.72E-05	7.254369	Up	anillin actin binding protein
A_33_P3294372	VPS9D1-AS1	1.306092	2.41E-06	6.77E-05	7.248319	Up	VPS9D1 antisense RNA 1
A_33_P3265744	PTGER3	1.575019	2.54E-06	7.08E-05	7.216057	Up	prostaglandin E receptor 3
A_23_P394395	JPH2	1.908297	2.56E-06	7.14E-05	7.210289	Up	junctophilin 2
A_23_P253123	VGLL1	2.118988	2.68E-06	7.4E-05	7.18483	Up	vestigial like family member 1
A_23_P413641	PREX1	2.025524	2.73E-06	7.5E-05	7.173834	Up	phosphatidylinositol-3,4,5-trisphosphate dependent Rac exchange factor 1
A_33_P3278362	ANKRD2	1.709802	2.76E-06	7.57E-05	7.166348	Up	ankyrin repeat domain 2
A_33_P3351554	ETNK2	1.491329	2.77E-06	7.58E-05	7.165379	Up	ethanolamine kinase 2
A_23_P94795	TEAD4	1.479463	2.95E-06	7.98E-05	7.12802	Up	TEA domain transcription factor 4
A_23_P132760	TRH	1.241853	2.96E-06	8E-05	7.124363	Up	thyrotropin releasing hormone
A_23_P161615	POLA2	1.553471	2.98E-06	8.02E-05	7.121807	Up	DNA polymerase alpha 2, accessory subunit
A_33_P3392187	CCDC85A	1.79453	3.06E-06	8.2E-05	7.105197	Up	coiled-coil domain containing 85A
A_24_P11315	OLFML3	2.397827	3.17E-06	8.44E-05	7.083713	Up	olfactomedin like 3
A_23_P318904	SERTAD4	1.230602	3.18E-06	8.45E-05	7.082651	Up	SERTA domain containing 4
A_23_P24457	LRRC4C	1.31262	3.25E-06	8.59E-05	7.069531	Up	leucine rich repeat containing 4C
A_33_P3249414	TRIM58	1.420226	3.34E-06	8.8E-05	7.053392	Up	tripartite motif containing 58
A_23_P257111	FBP1	1.24555	3.39E-06	8.89E-05	7.044519	Up	fructose-bisphosphatase 1
A_23_P21436	PHF19	1.212638	3.42E-06	8.94E-05	7.039658	Up	PHD finger protein 19
A_23_P65918	ITPKA	1.46634	3.45E-06	8.98E-05	7.034954	Up	inositol-trisphosphate 3-kinase A
A_23_P159316	BFSP2	2.079677	3.48E-06	9.05E-05	7.029798	Up	beaded filament structural protein 2
A_23_P102391	SLC40A1	1.277083	3.62E-06	9.31E-05	7.006994	Up	solute carrier family 40 member 1
A_23_P133123	MND1	1.453367	3.62E-06	9.32E-05	7.006473	Up	meiotic nuclear divisions 1
A_33_P3355014	TMEM229B	1.389628	3.9E-06	9.87E-05	6.9631	Up	transmembrane protein 229B
A_23_P103328	PTGER3	1.708427	3.99E-06	0.000101	6.949995	Up	prostaglandin E receptor 3
A_24_P383901	AL080243.1	1.219987	4.01E-06	0.000101	6.945906	Up	NA
A_23_P35871	E2F8	1.502242	4.06E-06	0.000101	6.939476	Up	E2F transcription factor 8
A_24_P218979	CDCA3	1.274266	4.39E-06	0.000108	6.894318	Up	cell division cycle associated 3
A_33_P3358243	KIAA1161	1.593236	4.55E-06	0.000111	6.872589	Up	KIAA1161
A_33_P3772937	KRT8P12	1.540186	4.57E-06	0.000112	6.870248	Up	keratin 8 pseudogene 12
A_24_P2648	PTPN14	1.381809	4.7E-06	0.000114	6.854188	Up	protein tyrosine phosphatase, non-receptor type 14
A_23_P43800	BOP1	1.260974	5.45E-06	0.000128	6.769022	Up	block of proliferation 1
A_24_P67395	KRT8	1.650833	5.8E-06	0.000136	6.733438	Up	keratin 8
A_24_P314571	SPC24	1.467398	6.09E-06	0.000141	6.705326	Up	SPC24, NDC80 kinetochore complex component
A_23_P166087	RASSF2	1.290762	6.14E-06	0.000142	6.700171	Up	Ras association domain family member 2
A_32_P154473	KIF5C	1.33259	6.17E-06	0.000142	6.69747	Up	kinesin family member 5C
A_33_P3336686	CLIC3	1.29647	6.25E-06	0.000144	6.690263	Up	chloride intracellular channel 3
A_23_P388812	CKAP2L	1.208228	6.31E-06	0.000145	6.685073	Up	cytoskeleton associated protein 2 like
A_32_P489	LOC100132735	2.411846	6.61E-06	0.00015	6.658005	Up	uncharacterized LOC100132735
A_33_P3281745	AL138760.1	1.692809	6.79E-06	0.000154	6.642797	Up	NA
A_33_P3363420	FRMD3	1.584154	7.09E-06	0.000159	6.617875	Up	FERM domain containing 3
A_33_P3232498	C2orf71	1.957637	7.6E-06	0.000169	6.578502	Up	chromosome 2 open reading frame 71
A_23_P42909	TMEM139	1.468495	7.62E-06	0.000169	6.577324	Up	transmembrane protein 139
A_23_P71880	SPINK4	4.399577	7.93E-06	0.000175	6.554657	Up	serine peptidase inhibitor, Kazal type 4
A_32_P211045	DHFR	1.660058	7.97E-06	0.000175	6.552102	Up	dihydrofolatereductase
A_33_P3265749	PTGER3	1.57945	8.29E-06	0.000182	6.529537	Up	prostaglandin E receptor 3
A_23_P35617	PLCE1	1.654785	8.37E-06	0.000183	6.524118	Up	phospholipase C epsilon 1
A_23_P69179	P3H2	1.344462	8.42E-06	0.000184	6.520608	Up	prolyl 3-hydroxylase 2
A_24_P6903	ACTBL2	1.234632	8.61E-06	0.000188	6.50808	Up	actin, beta like 2
A_33_P3350967	LINC00992	1.427673	9.81E-06	0.000209	6.434643	Up	long intergenic non-protein coding RNA 992

A_33_P3244803	ACOX1	1.271324	9.86E-06	0.000209	6.432035	Up	acyl-CoA oxidase 1
A_24_P406601	OLFM1	2.996465	1E-05	0.000212	6.423346	Up	olfactomedin 1
A_24_P404033	ARPIN	1.252601	1.02E-05	0.000215	6.414551	Up	actin-related protein 2/3 complex inhibitor
A_23_P102611	WISP2	1.911919	1.03E-05	0.000217	6.408638	Up	WNT1 inducible signaling pathway protein 2
A_33_P3352098	MS4A7	1.435775	1.05E-05	0.00022	6.397684	Up	membrane spanning 4-domains A7
A_24_P178631	CU634019.6	1.407875	1.05E-05	0.000221	6.395143	Up	NA
A_23_P335981	HOXC12	1.306287	1.07E-05	0.000224	6.385314	Up	homeobox C12
A_33_P3331511	LINC00173	1.473155	1.16E-05	0.000238	6.341628	Up	long intergenic non-protein coding RNA 173
A_33_P3365988	TGFB2-AS1	1.415387	1.17E-05	0.00024	6.336786	Up	TGFB2 antisense RNA 1 (head to head)
A_23_P350045	REEP5	1.251937	1.26E-05	0.000255	6.294923	Up	receptor accessory protein 5
A_32_P131640	SLITRK4	1.458493	1.54E-05	0.000299	6.185861	Up	SLIT and NTRK like family member 4
A_33_P3423830	TCAF1	1.478363	1.56E-05	0.000304	6.175784	Up	TRPM8 channel associated factor 1
A_24_P323148	LYPD5	1.386445	1.6E-05	0.000311	6.162293	Up	LY6/PLAUR domain containing 5
A_23_P353524	IVL	1.229862	1.63E-05	0.000314	6.154474	Up	involucrin
A_23_P33875	SSX4B	1.331625	1.65E-05	0.000317	6.146715	Up	SSX family member 4B
A_23_P96325	ERCC6L	1.562756	1.66E-05	0.000319	6.14203	Up	ERCC excision repair 6 like, spindle assembly checkpoint helicase
A_33_P3346891	MYBL1	1.438613	1.75E-05	0.000331	6.113882	Up	MYB proto-oncogene like 1
A_24_P357266	GRPR	2.583214	1.83E-05	0.000344	6.089923	Up	gastrin releasing peptide receptor
A_33_P3234020	IGDCC3	1.480564	1.86E-05	0.000348	6.081324	Up	immunoglobulin superfamily DCC subclass member 3
A_24_P401601	#N/A	1.242768	1.95E-05	0.000362	6.05478	Up	NA
A_24_P406986	SLC43A3	1.223654	2E-05	0.000368	6.042503	Up	solute carrier family 43 member 3
A_33_P3240702	RBBP8	1.193868	2.01E-05	0.00037	6.039627	Up	RB binding protein 8, endonuclease
A_23_P258493	LMNB1	1.424708	2.01E-05	0.00037	6.038602	Up	lamin B1
A_23_P252306	ID1	1.666772	2.05E-05	0.000375	6.028525	Up	inhibitor of DNA binding 1, HLH protein
A_19_P00811284	AC022182.3	1.521071	2.16E-05	0.000392	6.000606	Up	NA
A_32_P199429	NCAM2	1.656707	2.2E-05	0.000398	5.989807	Up	neural cell adhesion molecule 2
A_23_P100660	SERPINF1	1.214149	2.34E-05	0.000419	5.955146	Up	serpin family F member 1
A_23_P147918	S100A16	1.296011	2.55E-05	0.000447	5.909425	Up	S100 calcium binding protein A16
A_23_P117363	SERPINA6	2.8186	2.58E-05	0.000451	5.902803	Up	serpin family A member 6
A_33_P3216008	SKA3	1.231972	2.66E-05	0.000461	5.887481	Up	spindle and kinetochore associated complex subunit 3
A_32_P56525	TCAF1	1.569136	2.7E-05	0.000466	5.878984	Up	TRPM8 channel associated factor 1
A_23_P158096	COL27A1	1.531271	2.82E-05	0.000484	5.854882	Up	collagen type XXVII alpha 1 chain
A_33_P3248354	OLFM1	3.078285	3.03E-05	0.000511	5.817635	Up	olfactomedin 1
A_23_P163481	BUB1B	1.58082	3.26E-05	0.000545	5.777799	Up	BUB1 mitotic checkpoint serine/threonine kinase B
A_33_P3394694	LOC100128498	1.34801	3.59E-05	0.000586	5.727253	Up	uncharacterized LOC100128498
A_33_P3350493	CLPSL2	1.566803	4.31E-05	0.000685	5.63014	Up	colipase like 2
A_24_P307854	FBXL18	1.216303	4.43E-05	0.000701	5.615092	Up	F-box and leucine rich repeat protein 18
A_33_P3360072	RANBP3L	1.359278	4.62E-05	0.000726	5.593046	Up	RAN binding protein 3 like
A_33_P3403400	CLPSL1	1.424528	4.76E-05	0.000744	5.577079	Up	colipase like 1
A_33_P3411980	ABI3BP	2.267506	5.08E-05	0.000778	5.5431	Up	ABI family member 3 binding protein
A_23_P144656	CDH10	2.098146	5.56E-05	0.00084	5.495615	Up	cadherin 10
A_23_P24157	PYROXD2	1.203296	5.62E-05	0.000849	5.489715	Up	pyridine nucleotide-disulphide oxidoreductase domain 2
A_33_P3231739	ELOVL2	1.521507	5.64E-05	0.00085	5.488365	Up	ELOVL fatty acid elongase 2
A_23_P89431	CCL2	1.573389	5.77E-05	0.000867	5.476257	Up	C-C motif chemokine ligand 2
A_23_P77731	CRYM	1.622553	6.14E-05	0.000914	5.443578	Up	crystallin mu

A_23_P397480	ABCC13	1.355045	6.19E-05	0.000921	5.439448	Up	ATP binding cassette subfamily C member 13 (pseudogene)
A_24_P336718	RABIF	1.23078	6.72E-05	0.000981	5.397259	Up	RAB interacting factor
A_23_P133691	RRAGD	-3.14392	6.38E-15	2.31E-10	-29.181	Down	Ras related GTP binding D
A_24_P394533	NEU1	-2.35624	1.08E-14	2.31E-10	-28.1768	Down	neuraminidase 1
A_33_P3348244	BRI3	-2.56253	2.08E-14	2.36E-10	-26.9883	Down	brain protein I3
A_32_P8813	LINC00926	-3.05884	2.22E-14	2.36E-10	-26.8743	Down	long intergenic non-protein coding RNA 926
A_23_P81993	C6orf1	-2.28488	3.17E-14	2.7E-10	-26.2497	Down	chromosome 6 open reading frame 1
A_24_P382489	SLC27A1	-2.15686	3.9E-14	2.77E-10	-25.8906	Down	solute carrier family 27 member 1
A_23_P104318	DDIT4	-2.62018	2.02E-13	9.96E-10	-23.2122	Down	DNA damage inducible transcript 4
A_23_P114903	HSPA6	-3.82122	2.11E-13	9.96E-10	-23.1493	Down	heat shock protein family A (Hsp70) member 6
A_23_P7144	CXCL1	-3.34511	4.16E-13	1.77E-09	-22.1226	Down	C-X-C motif chemokine ligand 1
A_33_P3219090	INSIG1	-1.99383	8.21E-13	3.12E-09	-21.1407	Down	insulin induced gene 1
A_32_P95223	FDPSP2	-2.2297	8.8E-13	3.12E-09	-21.0434	Down	farnesyldiphosphate synthase pseudogene 2
A_23_P426021	SEL1L3	-1.96883	9.72E-13	3.18E-09	-20.9037	Down	SEL1L family member 3
A_23_P210690	TRIB3	-2.5687	1.26E-12	3.82E-09	-20.5448	Down	tribblespseudokinase 3
A_23_P144622	GNPDA1	-2.46317	1.35E-12	3.82E-09	-20.4519	Down	glucosamine-6-phosphate deaminase 1
A_23_P15402	SAT2	-1.89994	1.57E-12	4.17E-09	-20.2437	Down	spermidine/spermine N1-acetyltransferase family member 2
A_24_P103886	IDI1	-1.60352	2.14E-12	5.21E-09	-19.827	Down	isopentenyl-diphosphate delta isomerase 1
A_23_P152125	MVD	-2.57372	2.21E-12	5.21E-09	-19.7853	Down	mevalonatediphosphate decarboxylase
A_33_P3339246	LHX8	-2.13346	2.51E-12	5.61E-09	-19.6157	Down	LIM homeobox 8
A_23_P50504	FTL	-2.54844	2.84E-12	6.03E-09	-19.4534	Down	ferritin light chain
A_23_P75741	UBE2L6	-1.3938	3.11E-12	6.03E-09	-19.3328	Down	ubiquitin conjugating enzyme E2 L6
A_23_P9875	TESK2	-1.70649	3.12E-12	6.03E-09	-19.3306	Down	testis-specific kinase 2
A_33_P3356210	NCR3LG1	-2.81555	3.93E-12	6.96E-09	-19.0315	Down	natural killer cell cytotoxicity receptor 3 ligand 1
A_23_P104471	DUSP13	-2.69574	4.15E-12	7.06E-09	-18.96	Down	dual specificity phosphatase 13
A_33_P3269924	HIP1R	-2.33895	4.32E-12	7.06E-09	-18.9111	Down	huntingtin interacting protein 1 related
A_33_P3491294	AMDHD2	-1.84054	5.56E-12	8.4E-09	-18.5899	Down	amidohydrolase domain containing 2
A_23_P162142	TSKU	-2.26726	5.73E-12	8.4E-09	-18.5532	Down	tsukushi, small leucine rich proteoglycan
A_23_P122915	BRI3	-2.48865	5.94E-12	8.42E-09	-18.508	Down	brain protein I3
A_32_P155247	FTL	-2.55026	6.14E-12	8.42E-09	-18.4666	Down	ferritin light chain
A_23_P11859	HSD17B7	-2.18055	6.95E-12	9.15E-09	-18.3129	Down	hydroxysteroid 17-beta dehydrogenase 7
A_24_P382319	CEACAM1	-2.67258	7.1E-12	9.15E-09	-18.2866	Down	carcinoembryonic antigen related cell adhesion molecule 1
A_23_P1594	VEGFB	-1.75025	8.69E-12	1.09E-08	-18.0379	Down	vascular endothelial growth factor B
A_23_P126939	RAB29	-1.6814	9.07E-12	1.1E-08	-17.9854	Down	RAB29, member RAS oncogene family
A_24_P408047	PLEKHA4	-1.78609	9.48E-12	1.12E-08	-17.931	Down	pleckstrin homology domain containing A4
A_23_P134426	GPNMB	-3.73301	1.03E-11	1.18E-08	-17.8324	Down	glycoprotein nmb
A_23_P210900	ACSS2	-2.8789	1.09E-11	1.21E-08	-17.758	Down	acyl-CoA synthetase short-chain family member 2
A_23_P328069	HPS1	-1.41671	1.13E-11	1.21E-08	-17.7147	Down	HPS1, biogenesis of lysosomal organelles complex 3 subunit 1
A_33_P3283611	IFIT3	-3.36066	1.15E-11	1.21E-08	-17.6968	Down	interferon induced protein with tetratricopeptide repeats 3
A_23_P110212	ACSL1	-2.15062	1.16E-11	1.21E-08	-17.6848	Down	acyl-CoA synthetase long-chain family member 1
A_23_P26024	C15orf48	-2.56081	1.83E-11	1.79E-08	-17.1494	Down	chromosome 15 open reading

							frame 48
A_24_P114183	FDPS	-2.09636	1.83E-11	1.79E-08	-17.149	Down	farnesyldiphosphate synthase
A_23_P414308	FLCN	-2.28389	1.9E-11	1.79E-08	-17.1073	Down	folliculin
A_23_P17821	PLA2G3	-1.77446	1.96E-11	1.81E-08	-17.0706	Down	phospholipase A2 group III
A_23_P29773	LAMP3	-4.84253	2.04E-11	1.81E-08	-17.0204	Down	lysosomal associated membrane protein 3
A_24_P117410	KLHDC7B	-6.17994	2.08E-11	1.81E-08	-17.0004	Down	kelch domain containing 7B
A_33_P3271323	RHOQ	-2.11018	2.16E-11	1.84E-08	-16.9552	Down	ras homolog family member Q
A_19_P00812340	FTLP15	-2.54568	2.28E-11	1.87E-08	-16.8932	Down	ferritin light chain pseudogene 15
A_33_P3305790	NOS3	-3.60353	2.35E-11	1.87E-08	-16.8583	Down	nitric oxide synthase 3
A_23_P18447	PPARGC1A	-1.67525	2.38E-11	1.87E-08	-16.8446	Down	PPARG coactivator 1 alpha
A_23_P21134	DDIT3	-4.47779	2.44E-11	1.87E-08	-16.8142	Down	DNA damage inducible transcript 3
A_33_P3329088	PRSS8	-2.1146	2.54E-11	1.9E-08	-16.7694	Down	protease, serine 8
A_32_P45009	IDH1	-1.74742	2.61E-11	1.91E-08	-16.7392	Down	isocitrate dehydrogenase (NADP(+)) 1, cytosolic
A_23_P27013	HOXB9	-3.92675	3E-11	2.13E-08	-16.5801	Down	homeobox B9
A_23_P6535	KLHDC7B	-6.19651	3.68E-11	2.57E-08	-16.3519	Down	kelch domain containing 7B
A_23_P104996	BEST1	-3.07763	4.05E-11	2.76E-08	-16.246	Down	bestrophin 1
A_33_P3278573	MAGIX	-1.7451	4.17E-11	2.77E-08	-16.213	Down	MAGI family member, X-linked
A_23_P74778	C1orf54	-2.71976	4.44E-11	2.91E-08	-16.1432	Down	chromosome 1 open reading frame 54
A_32_P52609	LPIN1	-2.07337	4.66E-11	3.01E-08	-16.0893	Down	lipin 1
A_23_P217319	FGF13	-3.21104	4.74E-11	3.01E-08	-16.0699	Down	fibroblast growth factor 13
A_23_P397376	MAF	-1.86336	5.62E-11	3.47E-08	-15.884	Down	MAF bZIP transcription factor
A_23_P153745	IFI30	-2.42624	6.28E-11	3.81E-08	-15.765	Down	IFI30, lysosomalthiolreductase
A_23_P110184	MSMO1	-1.95108	6.72E-11	3.97E-08	-15.691	Down	methylsterolmonooxygenase 1
A_23_P34142	TCEAL9	-1.43397	6.83E-11	3.98E-08	-15.673	Down	transcription elongation factor A like 9
A_33_P3376095	SYPL2	-1.6491	7.21E-11	4.14E-08	-15.6158	Down	synaptophysin like 2
A_33_P3415087	CLCN5	-1.72655	7.41E-11	4.15E-08	-15.5866	Down	chloride voltage-gated channel 5
A_33_P3386671	RORC	-2.38424	7.55E-11	4.15E-08	-15.5655	Down	RAR related orphan receptor C
A_23_P47304	CASP5	-1.79663	8.62E-11	4.59E-08	-15.4246	Down	caspase 5
A_23_P129334	CLCN7	-1.77542	1.02E-10	5.19E-08	-15.2428	Down	chloride voltage-gated channel 7
A_23_P142075	ACP5	-2.02652	1.06E-10	5.27E-08	-15.2052	Down	acid phosphatase 5, tartrate resistant
A_23_P135164	UAP1L1	-2.76575	1.07E-10	5.27E-08	-15.202	Down	UDP-N-acetylglucosaminepyrophosphorylase 1 like 1
A_23_P117163	RCBTB1	-1.60574	1.2E-10	5.86E-08	-15.0794	Down	RCC1 and BTB domain containing protein 1
A_23_P64828	OAS1	-3.26876	1.31E-10	6.24E-08	-14.9902	Down	2'-5'-oligoadenylate synthetase 1
A_23_P153320	ICAM1	-3.3689	1.41E-10	6.66E-08	-14.9111	Down	intercellular adhesion molecule 1
A_23_P157580	SDCBP	-2.1227	1.43E-10	6.69E-08	-14.895	Down	syndecan binding protein
A_33_P3365142	GAD1	-1.39577	1.63E-10	7.48E-08	-14.7598	Down	glutamate decarboxylase 1
A_33_P3230598	ERVV-2	-2.25316	1.71E-10	7.66E-08	-14.7132	Down	endogenous retrovirus group V member 2
A_23_P169437	LCN2	-4.11453	1.94E-10	8.17E-08	-14.5865	Down	lipocalin 2
A_32_P214340	AL139260.1	-1.90384	1.94E-10	8.17E-08	-14.5846	Down	NA
A_33_P3419910	GGCT	-1.90927	1.96E-10	8.17E-08	-14.5761	Down	gamma-glutamylcyclotransferase
A_23_P30495	HMGCR	-1.69018	1.99E-10	8.21E-08	-14.5607	Down	3-hydroxy-3-methylglutaryl-CoA reductase
A_23_P16523	GDF15	-4.39511	2.04E-10	8.36E-08	-14.5326	Down	growth differentiation factor 15
A_33_P3382331	HSPA6	-2.49262	2.51E-10	9.88E-08	-14.3284	Down	heat shock protein family A (Hsp70) member 6
A_23_P160318	COL16A1	-2.9252	2.69E-10	1.04E-07	-14.2587	Down	collagen type XVI alpha 1 chain
A_23_P78209	MAFG	-2.23696	2.71E-10	1.04E-07	-14.2528	Down	MAF bZIP transcription factor G
A_23_P31453	STEAP1	-1.5725	2.78E-10	1.05E-07	-14.2273	Down	STEAP family member 1
A_24_P63522	HMGCS1	-1.79373	2.82E-10	1.05E-07	-14.2132	Down	3-hydroxy-3-methylglutaryl-CoA synthase 1
A_33_P3405334	GM2A	-1.3627	3.02E-10	1.1E-07	-14.144	Down	GM2 ganglioside activator
A_23_P146798	SEPHS2	-3.10513	3.18E-10	1.12E-07	-14.0933	Down	selenophosphatesynthetase 2
A_23_P431346	PRR15	-1.92937	3.34E-10	1.16E-07	-14.0456	Down	proline rich 15
A_24_P933908	GPNMB	-2.90759	3.4E-10	1.17E-07	-14.028	Down	glycoprotein nmb
A_32_P452655	LGALS9C	-1.68341	3.45E-10	1.17E-07	-14.0136	Down	galectin 9C
A_33_P3399788	SERPINA3	-3.69263	3.7E-10	1.23E-07	-13.9472	Down	serpin family A member 3

A_24_P185117	RILP	-1.57104	3.88E-10	1.26E-07	-13.9	Down	Rab interacting lysosomal protein
A_23_P332399	GULP1	-3.30529	3.89E-10	1.26E-07	-13.8969	Down	GULP, engulfment adaptor PTB domain containing 1
A_32_P217750	IL3RA	-2.13113	3.91E-10	1.26E-07	-13.892	Down	interleukin 3 receptor subunit alpha
A_19_P00320597	AP002004.1	-1.85459	3.99E-10	1.27E-07	-13.8732	Down	NA
A_33_P3343467	LINC00957	-1.42378	4.05E-10	1.27E-07	-13.8586	Down	long intergenic non-protein coding RNA 957
A_23_P360626	PLD6	-1.90942	4.05E-10	1.27E-07	-13.8578	Down	phospholipase D family member 6
A_33_P3222380	AHNAK2	-1.49924	4.14E-10	1.29E-07	-13.8376	Down	AHNAK nucleoprotein 2
A_33_P3276369	HSD17B7	-2.10289	4.89E-10	1.47E-07	-13.6784	Down	hydroxysteroid 17-beta dehydrogenase 7
A_33_P3225487	HSD17B7	-2.08512	5.74E-10	1.72E-07	-13.5253	Down	hydroxysteroid 17-beta dehydrogenase 7
A_23_P13364	NUCB2	-1.93346	6.02E-10	1.77E-07	-13.4801	Down	nucleobindin 2
A_33_P3329740	SDCBPP3	-2.31645	6.03E-10	1.77E-07	-13.4786	Down	syndecan binding protein pseudogene 3
A_23_P171077	EBP	-1.36443	6.07E-10	1.77E-07	-13.4733	Down	emopamil binding protein (sterol isomerase)
A_23_P211252	LSS	-1.83266	6.21E-10	1.8E-07	-13.451	Down	lanosterol synthase
A_33_P3415092	CLCN5	-1.73021	6.35E-10	1.8E-07	-13.4295	Down	chloride voltage-gated channel 5
A_23_P2920	SERPINA3	-3.71422	6.43E-10	1.8E-07	-13.4188	Down	serpin family A member 3
A_23_P117298	F7	-2.56787	6.47E-10	1.8E-07	-13.4129	Down	coagulation factor VII
A_23_P105862	FRY	-1.75735	6.5E-10	1.8E-07	-13.4088	Down	FRY microtubule binding protein
A_23_P410717	CIART	-2.08659	6.55E-10	1.8E-07	-13.4004	Down	circadian associated repressor of transcription
A_23_P156687	CFB	-2.143	6.74E-10	1.83E-07	-13.3741	Down	complement factor B
A_23_P46852	STN1	-1.3516	7.03E-10	1.88E-07	-13.3352	Down	STN1, CST complex subunit
A_23_P134347	CPVL	-1.60416	7.09E-10	1.89E-07	-13.3269	Down	carboxypeptidase, vitellogenic like
A_24_P110799	LSS	-1.89845	7.31E-10	1.93E-07	-13.2983	Down	lanosterol synthase
A_23_P354208	WDR81	-1.46829	7.66E-10	1.99E-07	-13.2553	Down	WD repeat domain 81
A_19_P00318331	NUCB2	-2.5376	7.9E-10	2.04E-07	-13.2267	Down	nucleobindin 2
A_23_P5778	RAB17	-1.58166	7.98E-10	2.04E-07	-13.2165	Down	RAB17, member RAS oncogene family
A_32_P66035	TMEM86A	-1.86192	8.59E-10	2.18E-07	-13.1486	Down	transmembrane protein 86A
A_33_P3330264	CXCL1	-2.44235	8.69E-10	2.19E-07	-13.1385	Down	C-X-C motif chemokine ligand 1
A_23_P81399	SQSTM1	-2.38953	9.8E-10	2.42E-07	-13.0273	Down	sequestosome 1
A_24_P388528	ST6GAL1	-2.01035	1E-09	2.46E-07	-13.0073	Down	ST6 beta-galactoside alpha-2,6-sialyltransferase 1
A_23_P18684	CLGN	-2.65266	1.01E-09	2.47E-07	-13	Down	calmegin
A_33_P3419912	GGCT	-2.11057	1.05E-09	2.53E-07	-12.9675	Down	gamma-glutamylcyclotransferas
A_23_P35912	CASP4	-1.93771	1.07E-09	2.56E-07	-12.9507	Down	caspase 4
A_33_P3209229	RAB26	-2.18628	1.17E-09	2.81E-07	-12.8623	Down	RAB26, member RAS oncogene family
A_24_P406334	STEAP1	-1.54092	1.2E-09	2.85E-07	-12.8396	Down	STEAP family member 1
A_33_P3357653	PSMG2	-2.03444	1.26E-09	2.96E-07	-12.8002	Down	proteasome assembly chaperone 2
A_24_P521994	KLHL24	-2.07066	1.28E-09	3E-07	-12.7814	Down	kelch like family member 24
A_23_P52336	UNC5B	-3.49857	1.34E-09	3.11E-07	-12.7405	Down	unc-5 netrin receptor B
A_19_P00322986	#N/A	-1.38535	1.35E-09	3.11E-07	-12.7368	Down	NA
A_33_P3361422	CYP27A1	-1.59718	1.35E-09	3.11E-07	-12.7356	Down	cytochrome P450 family 27 subfamily A member 1
A_23_P327519	STARD4	-1.77897	1.4E-09	3.2E-07	-12.7056	Down	StAR related lipid transfer domain containing 4
A_23_P149775	ARHGAP12	-1.50828	1.41E-09	3.21E-07	-12.6957	Down	Rho GTPase activating protein 12
A_23_P151975	RHCG	-3.52187	1.58E-09	3.52E-07	-12.5987	Down	Rh family C glycoprotein
A_23_P162918	SERPINA3	-3.52863	1.58E-09	3.52E-07	-12.5959	Down	serpin family A member 3
A_33_P3514487	VSTM1	-1.91763	1.65E-09	3.61E-07	-12.558	Down	V-set and transmembrane domain containing 1
A_23_P65967	SLC38A7	-1.3627	1.65E-09	3.61E-07	-12.5561	Down	solute carrier family 38 member 7
A_23_P170608	TSPYL2	-1.60237	1.7E-09	3.7E-07	-12.5291	Down	TSPY like 2
A_23_P16915	QPCT	-1.62165	1.76E-09	3.81E-07	-12.498	Down	glutaminyl-peptide cyclotransferase
A_24_P139943	HS1BP3	-1.82691	1.95E-09	4E-07	-12.4109	Down	HCLS1 binding protein 3

A_32_P230148	#N/A	-2.39676	2.21E-09	4.42E-07	-12.2981	Down	NA
A_23_P139786	OASL	-3.29184	2.32E-09	4.55E-07	-12.2567	Down	2'-5'-oligoadenylate synthetase like
A_33_P3232011	RAB17	-1.6434	2.35E-09	4.57E-07	-12.2465	Down	RAB17, member RAS oncogene family
A_23_P75800	RAB3IL1	-2.88666	2.46E-09	4.72E-07	-12.2079	Down	RAB3A interacting protein like 1
A_23_P31896	ST3GAL1	-1.94825	2.54E-09	4.84E-07	-12.1804	Down	ST3 beta-galactoside alpha-2,3-sialyltransferase 1
A_23_P108294	PLPP2	-1.42611	2.71E-09	5.08E-07	-12.1226	Down	phospholipid phosphatase 2
A_23_P72737	IFITM1	-2.08499	2.79E-09	5.16E-07	-12.0982	Down	interferon induced transmembrane protein 1
A_23_P210330	LGALSL	-1.89977	3.05E-09	5.54E-07	-12.0224	Down	galectin like
A_33_P3278684	AL513217.1	-1.90856	3.1E-09	5.6E-07	-12.0073	Down	NA
A_33_P3283828	CCDC154	-1.94046	3.14E-09	5.62E-07	-11.9968	Down	coiled-coil domain containing 154
A_24_P77082	KMO	-1.76488	3.25E-09	5.67E-07	-11.9667	Down	kynurenine 3-monooxygenase
A_33_P3221303	CCR10	-1.37085	3.29E-09	5.72E-07	-11.9556		C-C motif chemokine receptor 10
A_33_P3407324	GULP1	-2.83795	3.56E-09	6.05E-07	-11.8902	Down	GULP, engulfment adaptor PTB domain containing 1
A_32_P205624	SHC2	-2.02023	4.05E-09	6.56E-07	-11.7807	Down	SHC adaptor protein 2
A_24_P154037	IRS2	-2.32029	4.08E-09	6.57E-07	-11.7745	Down	insulin receptor substrate 2
A_23_P26954	VAT1	-1.78694	4.1E-09	6.58E-07	-11.77	Down	vesicle amine transport 1
A_23_P137157	RENBP	-2.113	4.13E-09	6.6E-07	-11.764		renin binding protein
A_33_P3256391	CRB3	-1.36757	4.22E-09	6.7E-07	-11.7451	Down	crumbs 3, cell polarity complex component
A_23_P502078	MAPK8IP2	-1.76992	4.29E-09	6.75E-07	-11.7326	Down	mitogen-activated protein kinase 8 interacting protein 2
A_23_P819	ISG15	-2.37184	4.35E-09	6.78E-07	-11.7195	Down	ISG15 ubiquitin-like modifier
A_23_P165989	NEURL2	-1.40916	4.39E-09	6.81E-07	-11.7127	Down	neuralized E3 ubiquitin protein ligase 2
A_23_P15146	IL32	-2.93057	4.71E-09	7.14E-07	-11.6531	Down	interleukin 32
A_23_P62764	CCDC28B	-1.42251	4.86E-09	7.27E-07	-11.628	Down	coiled-coil domain containing 28B
A_23_P65442	IRF9	-2.21817	4.92E-09	7.32E-07	-11.6166	Down	interferon regulatory factor 9
A_24_P130041	CYP51A1	-1.60963	4.96E-09	7.32E-07	-11.6109	Down	cytochrome P450 family 51 subfamily A member 1
A_24_P290163	SMG1P1	-1.87941	5.29E-09	7.71E-07	-11.5565	Down	SMG1P1, nonsense mediated mRNA decay associated PI3K related kinase pseudogene 1
A_23_P121253	TNFSF10	-1.59713	5.31E-09	7.71E-07	-11.5534	Down	TNF superfamily member 10
A_23_P78108	ALDOC	-1.43768	5.46E-09	7.88E-07	-11.5302	Down	aldolase, fructose-bisphosphate C
A_23_P24004	IFIT2	-3.04996	5.8E-09	8.17E-07	-11.4805	Down	interferon induced protein with tetratricopeptide repeats 2
A_24_P117029	LDLR	-1.64462	6.42E-09	8.87E-07	-11.3964	Down	low density lipoprotein receptor
A_33_P3315314	MT1H	-2.65313	6.47E-09	8.88E-07	-11.3911	Down	metallothionein 1H
A_19_P00806129	#N/A	-1.43011	6.77E-09	9.14E-07	-11.3541	Down	NA
A_33_P3413905	ADM2	-1.69031	7.03E-09	9.47E-07	-11.3222	Down	adrenomedullin 2
A_23_P108657	WDSUB1	-1.44782	7.37E-09	9.9E-07	-11.2839	Down	WD repeat, sterile alpha motif and U-box domain containing 1
A_19_P00803598	MIR148A	-1.70789	7.47E-09	9.96E-07	-11.2732	Down	microRNA 148a
A_24_P173124	FLCN	-1.49094	7.83E-09	1.03E-06	-11.2355	Down	folliculin
A_23_P139500	BHLHE41	-1.93977	7.91E-09	1.03E-06	-11.2263	Down	basic helix-loop-helix family member e41
A_33_P3251093	SLC36A1	-1.57491	7.92E-09	1.03E-06	-11.2261	Down	solute carrier family 36 member 1
A_32_P4199	RNF152	-1.87155	8.08E-09	1.05E-06	-11.2097	Down	ring finger protein 152
A_23_P203743	GAB2	-1.35987	8.41E-09	1.07E-06	-11.177	Down	GRB2 associated binding protein 2
A_19_P00329765	AC124798.1	-2.78692	8.44E-09	1.07E-06	-11.1745	Down	NA
A_23_P43846	FABP6	-1.57533	9.01E-09	1.12E-06	-11.1219	Down	fatty acid binding protein 6
A_33_P3346826	IL32	-2.71399	9.31E-09	1.13E-06	-11.0954	Down	interleukin 32
A_33_P3242623	SLC7A11	-3.07641	9.68E-09	1.16E-06	-11.0637	Down	solute carrier family 7 member 11
A_33_P3301010	SPIRE1	-2.32306	9.83E-09	1.17E-06	-11.0512	Down	spire type actin nucleation factor 1
A_23_P341223	KLHL21	-1.6896	9.9E-09	1.17E-06	-11.0459	Down	kelch like family member 21
A_33_P3378962	LOC644135	-3.27924	1.02E-08	1.19E-06	-11.0228	Down	uncharacterized LOC644135
A_23_P32404	ISG20	-3.06554	1.06E-08	1.22E-06	-10.9918	Down	interferon stimulated exonuclease gene 20
A_23_P334021	IGF2R	-1.53906	1.09E-08	1.24E-06	-10.9713	Down	insulin like growth factor 2

							receptor
A_23_P409438	IFNL2	-3.22569	1.11E-08	1.26E-06	-10.9575	Down	interferon lambda 2
A_19_P00801515	RNU2-22P	-1.86819	1.11E-08	1.26E-06	-10.9548	Down	RNA, U2 small nuclear 22, pseudogene
A_33_P3405369	KRTAP5-4	-2.29516	1.15E-08	1.3E-06	-10.9239	Down	keratin associated protein 5-4
A_23_P97623	RRAGC	-1.63142	1.16E-08	1.31E-06	-10.9165	Down	Ras related GTP binding C
A_23_P29953	IL15	-2.14572	1.28E-08	1.38E-06	-10.8441	Down	interleukin 15
A_23_P117992	ATP2C2	-1.4033	1.32E-08	1.42E-06	-10.8147	Down	ATPase secretory pathway Ca2+ transporting 2
A_23_P17065	CCL20	-3.62904	1.33E-08	1.42E-06	-10.814	Down	C-C motif chemokine ligand 20
A_33_P3416767	MAFG	-2.17606	1.33E-08	1.42E-06	-10.8092	Down	MAF bZIP transcription factor G
A_23_P106145	ERO1A	-2.035	1.34E-08	1.42E-06	-10.8078	Down	endoplasmic reticulum oxidoreductase 1 alpha
A_23_P24444	DHCR7	-1.55123	1.36E-08	1.43E-06	-10.7963	Down	7-dehydrocholesterol reductase
A_33_P3225512	OAS2	-1.47066	1.37E-08	1.43E-06	-10.7892	Down	2'-5'-oligoadenylate synthetase 2
A_24_P71468	QPCT	-1.54873	1.43E-08	1.47E-06	-10.7547	Down	glutaminyl-peptide cyclotransferase
A_33_P3372332	CYBA	-1.5743	1.44E-08	1.47E-06	-10.7507	Down	cytochrome b-245 alpha chain
A_23_P7361	ELOVL6	-1.39741	1.5E-08	1.52E-06	-10.7189	Down	ELOVL fatty acid elongase 6
A_19_P00322595	#N/A	-2.75822	1.55E-08	1.56E-06	-10.6939	Down	NA
A_23_P382775	BBC3	-2.42966	1.63E-08	1.63E-06	-10.6527	Down	BCL2 binding component 3
A_24_P183150	CXCL3	-1.41794	1.64E-08	1.63E-06	-10.6481	Down	C-X-C motif chemokine ligand 3
A_23_P40880	CMTM8	-1.60526	1.69E-08	1.66E-06	-10.6264	Down	CKLF like MARVEL transmembrane domain containing 8
A_33_P3236868	MT1X	-3.38556	1.7E-08	1.67E-06	-10.6177	Down	metallothionein 1X
A_23_P37983	MT1B	-2.46767	1.71E-08	1.67E-06	-10.6171	Down	metallothionein 1B
A_33_P3327822	SH3BGR	-2.67724	1.73E-08	1.68E-06	-10.6081	Down	SH3 domain binding glutamate rich protein
A_23_P360316	FUT3	-2.19786	1.74E-08	1.68E-06	-10.6022	Down	fucosyltransferase 3 (Lewis blood group)
A_19_P00322594	#N/A	-3.07197	1.74E-08	1.68E-06	-10.6014	Down	NA
A_23_P5903	SLCO4A1	-2.46506	1.75E-08	1.68E-06	-10.5989	Down	solute carrier organic anion transporter family member 4A1
A_23_P120883	HMOX1	-2.88395	1.81E-08	1.72E-06	-10.572	Down	hemeoxygenase 1
A_23_P17663	MX1	-2.79531	1.89E-08	1.78E-06	-10.5358	Down	MX dynamin like GTPase 1
A_23_P119362	EMP3	-3.06785	1.9E-08	1.78E-06	-10.5332	Down	epithelial membrane protein 3
A_23_P77630	MAP1LC3B	-1.48556	2.03E-08	1.87E-06	-10.4834	Down	microtubule associated protein 1 light chain 3 beta
A_19_P00315641	LINC00632	-1.7104	2.07E-08	1.88E-06	-10.4678	Down	long intergenic non-protein coding RNA 632
A_32_P220715	MAP1LC3B	-1.42198	2.1E-08	1.9E-06	-10.4568	Down	microtubule associated protein 1 light chain 3 beta
A_23_P24311	ALDH3B2	-1.719	2.12E-08	1.91E-06	-10.4504	Down	aldehyde dehydrogenase 3 family member B2
A_24_P257416	CXCL2	-2.8407	2.12E-08	1.91E-06	-10.4495	Down	C-X-C motif chemokine ligand 2
A_32_P56249	USP30-AS1	-2.48487	2.18E-08	1.95E-06	-10.429	Down	USP30 antisense RNA 1
A_24_P691826	WFDC21P	-1.96577	2.28E-08	2.03E-06	-10.3938	Down	WAP four-disulfide core domain 21, pseudogen
A_23_P52266	IFIT1	-2.99456	2.33E-08	2.06E-06	-10.3757	Down	interferon induced protein with tetratricopeptide repeats 1
A_33_P3423941	IFITM1	-1.99453	2.34E-08	2.07E-06	-10.3735	Down	interferon induced transmembrane protein 1
A_19_P00321332	NEAT1	-1.7751	2.36E-08	2.07E-06	-10.3679	Down	nuclear paraspeckle assembly transcript 1
A_33_P3228709	KRTAP5-7	-1.88396	2.4E-08	2.1E-06	-10.3546	Down	keratin associated protein 5-7
A_33_P3344831	TMEM45A	-1.59015	2.4E-08	2.1E-06	-10.3533	Down	transmembrane protein 45A
A_24_P38815	TPP1	-1.77809	2.41E-08	2.1E-06	-10.3504	Down	tripeptidyl peptidase 1
A_23_P58266	S100P	-1.95717	2.51E-08	2.16E-06	-10.3219	Down	S100 calcium binding protein P
A_23_P328740	NEURL3	-4.67283	2.55E-08	2.19E-06	-10.3099	Down	neuralized E3 ubiquitin protein ligase 3
A_33_P3245178	BEX2	-1.77461	2.71E-08	2.29E-06	-10.263	Down	brain expressed X-linked 2
A_23_P315364	CXCL2	-3.16641	2.75E-08	2.32E-06	-10.2516	Down	C-X-C motif chemokine ligand 2
A_23_P98350	BIRC3	-2.05523	2.8E-08	2.35E-06	-10.238	Down	baculoviral IAP repeat containing 3
A_24_P206776	CRYAB	-1.65566	2.83E-08	2.37E-06	-10.2297	Down	crystallin alpha B

A_23_P54846	HERPUD1	-1.99771	2.85E-08	2.38E-06	-10.2244	Down	homocysteine inducible ER protein with ubiquitin like domain 1
A_23_P23074	IFI44	-2.1783	3.27E-08	2.6E-06	-10.1217	Down	interferon induced protein 44
A_23_P257971	AKR1C1	-2.99357	3.45E-08	2.72E-06	-10.0814	Down	aldo-ketoreductase family 1 member C1
A_33_P3390357	SPIRE1	-2.44964	3.6E-08	2.82E-06	-10.0486	Down	spire type actin nucleation factor 1
A_23_P41470	DDX60	-2.12373	3.7E-08	2.88E-06	-10.0295	Down	DExD/H-box helicase 60
A_23_P130753	DBP	-1.80604	3.82E-08	2.93E-06	-10.0051	Down	D-box binding PAR bZIP transcription factor
A_24_P273253	AHNAK2	-1.44159	3.88E-08	2.96E-06	-9.99349	Down	AHNAK nucleoprotein 2
A_23_P152838	CCL5	-3.28669	3.95E-08	3.01E-06	-9.97966	Down	C-C motif chemokine ligand 5
A_23_P92499	TLR2	-2.13432	4.06E-08	3.08E-06	-9.96086	Down	toll like receptor 2
A_23_P94230	LY96	-1.66916	4.07E-08	3.08E-06	-9.95915	Down	lymphocyte antigen 96
A_33_P3246448	KCNE4	-4.60819	4.07E-08	3.08E-06	-9.95782	Down	potassium voltage-gated channel subfamily E regulatory subunit 4
A_33_P3687198	LOC283454	-3.37219	4.24E-08	3.17E-06	-9.92887	Down	uncharacterized LOC283454
A_23_P427703	MT1L	-2.5541	4.29E-08	3.21E-06	-9.91879	Down	metallothionein 1L, pseudogene
A_23_P99163	DRAM1	-1.84682	4.38E-08	3.25E-06	-9.9035	Down	DNA damage regulated autophagy modulator 1
A_19_P00809519	#N/A	-1.48034	4.43E-08	3.28E-06	-9.89536	Down	NA
A_33_P3334047	#N/A	-1.52506	4.55E-08	3.35E-06	-9.8769	Down	NA
A_23_P408455	SLC25A36	-1.59793	4.69E-08	3.43E-06	-9.85426	Down	solute carrier family 25 member 36
A_33_P3392460	LOC100128077	-1.51769	4.82E-08	3.51E-06	-9.83367	Down	uncharacterized LOC100128077
A_32_P49848	RHOQ	-1.96482	4.83E-08	3.51E-06	-9.8322	Down	ras homolog family member Q
A_32_P191895	SDCBPP2	-1.973	4.85E-08	3.51E-06	-9.82986	Down	syndecan binding protein pseudogene 2
A_23_P206724	MT1E	-2.53822	5.11E-08	3.65E-06	-9.79137	Down	metallothionein 1E
A_23_P35082	SESN2	-1.66693	5.57E-08	3.89E-06	-9.72891	Down	sestrin 2
A_33_P3243449	CD70	-2.03861	5.63E-08	3.91E-06	-9.72088	Down	CD70 molecule
A_23_P53126	LMO2	-1.72077	5.75E-08	3.97E-06	-9.70595	Down	LIM domain only 2
A_23_P139143	STX3	-1.42298	5.8E-08	3.99E-06	-9.69946	Down	syntaxin 3
A_23_P138541	AKR1C3	-3.13726	5.93E-08	4.05E-06	-9.68354	Down	aldo-ketoreductase family 1 member C3
A_23_P36484	RDH16	-2.1202	6.18E-08	4.19E-06	-9.6529	Down	retinol dehydrogenase 16 (all-trans)
A_33_P3397763	TNFSF9	-1.56938	6.25E-08	4.22E-06	-9.64478	Down	TNF superfamily member 9
A_23_P250118	HSPBAP1	-2.18523	6.71E-08	4.45E-06	-9.59391	Down	HSPB1 associated protein 1
A_33_P3350056	MT1X	-2.67	7.07E-08	4.65E-06	-9.55665	Down	metallothionein 1X
A_24_P99639	NEK8	-1.4896	7.4E-08	4.82E-06	-9.52342	Down	NIMA related kinase 8
A_23_P258944	DNAJB9	-1.8812	8.13E-08	5.23E-06	-9.45639	Down	DnaJ heat shock protein family (Hsp40) member B9
A_19_P00320345	MICE	-1.51587	8.39E-08	5.34E-06	-9.43411	Down	MHC class I polypeptide-related sequence E (pseudogene)
A_24_P233786	FAM129A	-1.87798	8.51E-08	5.38E-06	-9.42428	Down	family with sequence similarity 129 member A
A_24_P317708	SDR16C5	-1.68526	9.27E-08	5.73E-06	-9.36324	Down	short chain dehydrogenase/reductase family 16C member 5
A_24_P58037	MAP1LC3BP1	-1.41261	9.33E-08	5.76E-06	-9.35924	Down	microtubule associated protein 1 light chain 3 beta pseudogene 1
A_24_P219552	NFE2L1	-1.44746	9.42E-08	5.8E-06	-9.35247	Down	nuclear factor, erythroid 2 like 1
A_23_P5200	NPHS1	-2.44492	9.54E-08	5.85E-06	-9.34324	Down	NPHS1, nephrin
A_24_P48898	APOL2	-1.3825	9.84E-08	5.98E-06	-9.3215	Down	apolipoprotein L2
A_33_P3375934	NAMPT	-2.01817	1E-07	6.08E-06	-9.30714	Down	nicotinamidephosphoribosyltransferase
A_33_P3228762	SLC25A36	-1.82205	1.09E-07	6.48E-06	-9.2477	Down	solute carrier family 25 member 36
A_23_P255104	LHFPL2	-1.80419	1.1E-07	6.48E-06	-9.24547	Down	lipoma HMGIC fusion partner-like 2
A_24_P329065	BTN3A1	-1.3609	1.11E-07	6.53E-06	-9.23709	Down	butyrophilin subfamily 3 member A1
A_23_P15174	MT1F	-3.77823	1.14E-07	6.69E-06	-9.21508	Down	metallothionein 1F

A_33_P3251108	SLC33A1	-1.4609	1.17E-07	6.8E-06	-9.19713	Down	solute carrier family 33 member 1
A_23_P78762	HSD17B14	-2.1241	1.2E-07	6.88E-06	-9.18389	Down	hydroxysteroid 17-beta dehydrogenase 14
A_33_P3252394	GADD45G	-2.10582	1.2E-07	6.91E-06	-9.18019	Down	growth arrest and DNA damage inducible gamma
A_23_P74012	SPRR1A	-2.20697	1.27E-07	7.18E-06	-9.14203	Down	small proline rich protein 1A
A_23_P337800	IFNL1	-2.13086	1.32E-07	7.38E-06	-9.11767	Down	interferon lambda 1
A_33_P3288844	IL6R	-1.97356	1.34E-07	7.45E-06	-9.10693	Down	interleukin 6 receptor
A_24_P141707	INHBE	-3.40673	1.37E-07	7.61E-06	-9.08802	Down	inhibin beta E subunit
A_23_P85893	GLMP	-1.35086	1.42E-07	7.82E-06	-9.06459	Down	glycosylated lysosomal membrane protein
A_24_P11061	CSAG1	-1.46479	1.46E-07	8E-06	-9.04513	Down	chondrosarcoma associated gene 1
A_23_P127781	SCGB1D1	-1.40866	1.52E-07	8.25E-06	-9.01861	Down	secretoglobin family 1D member 1
A_19_P00321333	NEAT1_2	-1.67685	1.54E-07	8.27E-06	-9.0098	Down	Nuclear enriched abundant transcript 1 conserved region 2
A_23_P109143	PRNP	-1.47608	1.54E-07	8.27E-06	-9.00978	Down	prion protein
A_19_P00321885	LINC-PINT	-1.38811	1.69E-07	8.95E-06	-8.94693	Down	long intergenic non-protein coding RNA, p53 induced transcript
A_32_P209960	CIITA	-1.4198	1.77E-07	9.29E-06	-8.91314	Down	class II major histocompatibility complex transactivator
A_33_P3263538	NEAT1	-2.39475	1.88E-07	9.65E-06	-8.87413	Down	nuclear paraspeckle assembly transcript 1 (non-protein coding)
A_23_P85693	GBP2	-1.46877	1.88E-07	9.66E-06	-8.87238	Down	guanylate binding protein 2
A_33_P3213029	RNF43	-1.51929	1.91E-07	9.76E-06	-8.86126	Down	ring finger protein 43
A_24_P314477	TUBB2B	-2.24296	2.14E-07	1.07E-05	-8.78677	Down	tubulin beta 2B class IIb
A_23_P75647	HYOU1	-1.69381	2.22E-07	1.1E-05	-8.75944	Down	hypoxia up-regulated 1
A_24_P98411	HSPA5	-2.29078	2.24E-07	1.11E-05	-8.75378	Down	heat shock protein family A (Hsp70) member 5
A_24_P190168	TMEM97	-1.82208	2.27E-07	1.11E-05	-8.74682	Down	transmembrane protein 97
A_33_P3287348	CHN2	-2.55684	2.32E-07	1.13E-05	-8.73156	Down	chimerin 2
A_33_P3350863	RETN	-1.79104	2.33E-07	1.14E-05	-8.72695	Down	resistin
A_23_P68155	IFIH1	-1.86075	2.38E-07	1.15E-05	-8.71328	Down	interferon induced with helicase C domain 1
A_23_P23221	GADD45A	-2.01735	2.39E-07	1.15E-05	-8.71194	Down	growth arrest and DNA damage inducible alpha
A_24_P941167	APOL6	-1.67281	2.54E-07	1.21E-05	-8.67016	Down	apolipoprotein L6
A_24_P250227	NR1D1	-1.35423	2.61E-07	1.23E-05	-8.65157	Down	nuclear receptor subfamily 1 group D member 1
A_33_P3217689	JMY	-1.68701	2.63E-07	1.24E-05	-8.64703	Down	junction mediating and regulatory protein, p53 cofactor
A_23_P65651	WARS	-1.43795	2.66E-07	1.25E-05	-8.63824	Down	tryptophanyl-tRNAsynthetase
A_24_P12401	VEGFA	-2.18374	3.01E-07	1.39E-05	-8.55633	Down	vascular endothelial growth factor A
A_23_P70398	VEGFA	-2.52564	3.1E-07	1.42E-05	-8.53819	Down	vascular endothelial growth factor A
A_24_P120934	GADD45G	-2.01668	3.32E-07	1.49E-05	-8.49155	Down	growth arrest and DNA damage inducible gamma
A_33_P3371718	SAT1	-1.64814	3.36E-07	1.51E-05	-8.48495	Down	spermidine/spermine N1-acetyltransferase 1
A_19_P00332245	#N/A	-1.7902	3.55E-07	1.57E-05	-8.44791	Down	NA
A_19_P00807637	#N/A	-1.66282	3.63E-07	1.6E-05	-8.43335	Down	NA
A_23_P82503	PEG10	-1.61632	3.68E-07	1.62E-05	-8.42458	Down	paternally expressed 10
A_23_P147805	UPP1	-2.03566	3.75E-07	1.65E-05	-8.41189	Down	uridinephosphorylase 1
A_33_P3260722	UVRAG	-1.61846	3.76E-07	1.65E-05	-8.40988	Down	UV radiation resistance associated
A_23_P124619	S100A14	-1.60264	3.97E-07	1.72E-05	-8.37452	Down	S100 calcium binding protein A14
A_33_P3350703	ERO1B	-1.76117	4.18E-07	1.79E-05	-8.34027	Down	endoplasmic reticulum oxidoreductase 1 beta
A_33_P3348744	AC069240.1	-1.53897	4.34E-07	1.85E-05	-8.31691	Down	NA
A_24_P305223	CTAGE1	-1.36568	4.39E-07	1.86E-05	-8.30838	Down	cutaneous T-cell lymphoma-associated antigen 1
A_23_P404606	CREBRF	-1.60721	4.58E-07	1.93E-05	-8.28104	Down	CREB3 regulatory factor

A_23_P146284	SQLE	-1.57607	4.62E-07	1.94E-05	-8.27582	Down	squaleneepoxidase
A_19_P00321089	LINC00963	-1.54637	4.63E-07	1.94E-05	-8.27474	Down	long intergenic non-protein coding RNA 963
A_19_P00809262	#N/A	-1.92729	4.77E-07	1.99E-05	-8.25571	Down	NA
A_33_P3335386	FAM83G	-1.58601	4.96E-07	2.06E-05	-8.22972	Down	family with sequence similarity 83 member G
A_23_P128246	FICD	-1.53856	5.13E-07	2.1E-05	-8.20844	Down	FIC domain containing
A_33_P3266025	COX5BP2	-2.93144	5.18E-07	2.12E-05	-8.20184	Down	cytochrome c oxidase subunit 5B pseudogene 2
A_23_P202881	FEZ1	-1.40829	5.29E-07	2.15E-05	-8.18785	Down	fasciculation and elongation protein zeta 1
A_33_P3363355	ICAM4	-1.4149	5.32E-07	2.15E-05	-8.18511	Down	intercellular adhesion molecule 4 (Landsteiner-Wiener blood group)
A_23_P159382	SEC24D	-1.51224	5.6E-07	2.24E-05	-8.15123	Down	SEC24 homolog D, COPII coat complex component
A_23_P40453	CBR3	-1.63468	5.69E-07	2.27E-05	-8.14133	Down	carbonyl reductase 3
A_32_P153388	GULP1	-1.83194	5.72E-07	2.27E-05	-8.13782	Down	GULP, engulfment adaptor PTB domain containing 1
A_23_P200838	KMO	-1.61362	5.73E-07	2.27E-05	-8.13674	Down	kynurenine 3-monooxygenase
A_23_P401106	PDE2A	-1.70101	5.74E-07	2.28E-05	-8.13605	Down	phosphodiesterase 2A
A_33_P3290443	SCARNA9	-1.35872	6.19E-07	2.41E-05	-8.08771	Down	small Cajal body-specific RNA 9
A_24_P152968	AKR1C1	-2.66648	6.5E-07	2.52E-05	-8.05601	Down	aldo-ketoreductase family 1 member C1
A_19_P00805330	AC112484.3	-1.37127	6.52E-07	2.52E-05	-8.05485	Down	NA
A_24_P28722	RSAD2	-2.38853	6.79E-07	2.6E-05	-8.02856	Down	radical S-adenosyl methionine domain containing 2
A_23_P25194	HRK	-2.57172	7.26E-07	2.74E-05	-7.98622	Down	harakiri, BCL2 interacting protein
A_23_P137016	SAT1	-1.58948	7.33E-07	2.75E-05	-7.97998	Down	spermidine/spermine N1-acetyltransferase 1
A_33_P3408320	CERS1	-1.35723	7.41E-07	2.78E-05	-7.97306	Down	ceramide synthase 1
A_23_P93269	ZNF165	-2.1976	7.55E-07	2.8E-05	-7.96098	Down	zinc finger protein 165
A_23_P64854	KRT75	-1.90771	8.6E-07	3.11E-05	-7.87871	Down	keratin 75
A_23_P54840	MT1A	-2.05521	8.63E-07	3.12E-05	-7.87689	Down	metallothionein 1A
A_33_P3806965	LCAL1	-2.43498	8.72E-07	3.14E-05	-7.87034	Down	lung cancer associated lncRNA 1
A_32_P34920	FOXD1	-1.90531	9.15E-07	3.26E-05	-7.84014	Down	forkhead box D1
A_32_P49844	RHOQ	-1.8219	9.65E-07	3.38E-05	-7.80696	Down	ras homolog family member Q
A_32_P175739	HK2	-1.62356	9.71E-07	3.39E-05	-7.80319	Down	hexokinase 2
A_33_P3347477	AP000844.2	-1.67091	1.05E-06	3.61E-05	-7.75692	Down	NA
A_23_P41765	IRF1	-2.06905	1.12E-06	3.79E-05	-7.7168	Down	interferon regulatory factor 1
A_23_P351275	UPP1	-1.9013	1.13E-06	3.83E-05	-7.70649	Down	uridinephosphorylase 1
A_23_P320113	SRXN1	-1.69178	1.15E-06	3.88E-05	-7.69728	Down	sulfiredoxin 1
A_23_P50508	PLA2G4C	-1.35877	1.18E-06	3.96E-05	-7.68191	Down	phospholipase A2 group IVC
A_23_P106844	MT2A	-2.28545	1.19E-06	3.98E-05	-7.67656	Down	metallothionein 2A
A_19_P00809313	MIR612	-2.25872	1.2E-06	4.02E-05	-7.66972	Down	microRNA 612
A_23_P373619	IFNL3	-1.47491	1.21E-06	4.02E-05	-7.66785	Down	interferon lambda 3
A_33_P3364864	NAMPT	-2.0905	1.27E-06	4.18E-05	-7.63684	Down	nicotinamidephosphoribosyltransferase
A_23_P134176	SOD2	-2.09077	1.32E-06	4.32E-05	-7.61457	Down	superoxide dismutase 2
A_32_P154342	SLCO4C1	-1.74675	1.35E-06	4.41E-05	-7.59728	Down	solute carrier organic anion transporter family member 4C1
A_24_P332623	CTAGE11P	-1.378	1.37E-06	4.45E-05	-7.5913	Down	CTAGE family member 11, pseudogene
A_23_P3204	MAPK6	-1.4846	1.37E-06	4.45E-05	-7.59081	Down	mitogen-activated protein kinase 6
A_32_P52153	UNC5B-AS1	-3.233	1.49E-06	4.77E-05	-7.53721	Down	UNC5B antisense RNA 1
A_33_P3248265	LTB	-2.07004	1.52E-06	4.81E-05	-7.52754	Down	lymphotoxin beta
A_33_P3353030	UCN	-1.35048	1.55E-06	4.9E-05	-7.51253	Down	urocortin
A_24_P218265	TNFRSF10B	-1.65671	1.57E-06	4.95E-05	-7.50539	Down	TNF receptor superfamily member 10b
A_23_P64404	FADS3	-1.53093	1.71E-06	5.25E-05	-7.45409	Down	fatty acid desaturase 3
A_24_P329795	C10orf10	-1.35591	1.71E-06	5.25E-05	-7.45372	Down	chromosome 10 open reading frame 10
A_23_P145874	SAMD9L	-1.42032	1.74E-06	5.32E-05	-7.44392	Down	sterile alpha motif domain containing 9 like

A_23_P128744	BDKRB1	-2.30341	1.81E-06	5.5E-05	-7.41853	Down	bradykinin receptor B1
A_23_P428129	CDKN1C	-1.5539	1.83E-06	5.51E-05	-7.41423	Down	cyclin dependent kinase inhibitor 1C
A_23_P201808	PLPP3	-1.39173	1.9E-06	5.67E-05	-7.39039	Down	phospholipid phosphatase 3
A_23_P379026	GTPBP2	-2.21708	1.98E-06	5.84E-05	-7.36685	Down	GTP binding protein 2
A_23_P411296	CEBPB	-1.62166	2.04E-06	6.02E-05	-7.3465	Down	CCAAT/enhancer binding protein beta
A_23_P55706	RELB	-1.36401	2.13E-06	6.23E-05	-7.3202	Down	RELB proto-oncogene, NF-kB subunit
A_23_P111621	GTF2IRD1	-1.44116	2.14E-06	6.25E-05	-7.31803	Down	GTF2I repeat domain containing 1
A_23_P147025	RAB33A	-2.54376	2.27E-06	6.5E-05	-7.28281	Down	RAB33A, member RAS oncogene family
A_23_P104073	S100A3	-1.93486	2.33E-06	6.62E-05	-7.26871	Down	S100 calcium binding protein A3
A_24_P68908	NMRAL2P	-2.24345	2.74E-06	7.53E-05	-7.16993	Down	NmrA like redox sensor 2, pseudogene
A_23_P148410	FTHL17	-1.89202	2.9E-06	7.9E-05	-7.13635	Down	ferritin heavy chain like 17
A_23_P117782	LARP6	-2.3931	2.96E-06	7.99E-05	-7.12528	Down	La ribonucleoprotein domain family member 6
A_23_P52939	SLC43A1	-1.52546	3.15E-06	8.4E-05	-7.0884	Down	solute carrier family 43 member 1
A_24_P122921	BCL2L11	-1.74624	3.32E-06	8.76E-05	-7.05647	Down	BCL2 like 11
A_33_P3356462	C2CD4A	-2.52617	3.5E-06	9.09E-05	-7.02648	Down	C2 calcium dependent domain containing 4A
A_33_P3383611	KCP	-1.94671	3.53E-06	9.16E-05	-7.02057	Down	kielin/chordin-like protein
A_23_P24104	PLAU	-3.50612	3.54E-06	9.16E-05	-7.01986	Down	plasminogen activator, urokinase
A_19_P00318409	NEAT1_1	-1.6769	3.57E-06	9.22E-05	-7.01534	Down	Nuclear enriched abundant transcript 1 conserved region 1
A_23_P32233	KLF4	-1.77188	3.75E-06	9.59E-05	-6.98644	Down	Kruppel like factor 4
A_33_P3253747	CYP1A2	-1.78065	3.75E-06	9.6E-05	-6.98566	Down	cytochrome P450 family 1 subfamily A member 2
A_23_P340333	ITPRIP	-1.70305	3.85E-06	9.81E-05	-6.97049	Down	inositol 1,4,5-trisphosphate receptor interacting protein
A_33_P3705884	LINC00662	-1.52892	3.95E-06	9.98E-05	-6.95472	Down	long intergenic non-protein coding RNA 662
A_24_P304071	IFIT2	-2.19191	4.14E-06	0.000103	-6.92797	Down	interferon induced protein with tetratricopeptide repeats 2
A_24_P157926	TNFAIP3	-1.38088	4.19E-06	0.000104	-6.92148	Down	TNF alpha induced protein 3
A_19_P00320033	NEAT1	-1.63876	4.3E-06	0.000106	-6.9062	Down	nuclear paraspeckle assembly transcript 1 (non-protein coding)
A_23_P69810	GPAT3	-2.03281	4.37E-06	0.000108	-6.89595	Down	glycerol-3-phosphate acyltransferase 3
A_23_P135857	EIF2AK3	-1.47301	4.44E-06	0.000109	-6.88728	Down	eukaryotic translation initiation factor 2 alpha kinase 3
A_24_P365025	SPAG9	-1.38167	4.64E-06	0.000113	-6.86177	Down	sperm associated antigen 9
A_23_P26124	RORA	-1.836	5.06E-06	0.000121	-6.81171	Down	RAR related orphan receptor A
A_23_P76078	IL23A	-2.9759	5.32E-06	0.000126	-6.78276	Down	interleukin 23 subunit alpha
A_23_P20494	NDRG1	-1.40451	5.33E-06	0.000126	-6.78129	Down	N-myc downstream regulated 1
A_33_P3422802	ULBP1	-1.42076	5.88E-06	0.000137	-6.72557	Down	UL16 binding protein 1
A_23_P129903	TRIM16L	-1.93364	6.45E-06	0.000147	-6.67185	Down	tripartite motif containing 16 like
A_24_P137897	IFRD1	-1.88444	6.64E-06	0.000151	-6.65598	Down	interferon related developmental regulator 1
A_32_P176550	JMY	-1.36648	6.8E-06	0.000154	-6.64188	Down	junction mediating and regulatory protein, p53 cofactor
A_23_P61823	RAB24	-1.39304	7.02E-06	0.000158	-6.62354	Down	RAB24, member RAS oncogene family
A_33_P3282634	ALDH1L2	-1.47954	7.13E-06	0.00016	-6.61498	Down	aldehyde dehydrogenase 1 family member L2
A_33_P3774867	DLGAP1-AS2	-1.79449	7.92E-06	0.000175	-6.55513	Down	DLGAP1 antisense RNA 2
A_24_P92183	PABPC1L	-1.51119	8.49E-06	0.000186	-6.51592	Down	poly(A) binding protein cytoplasmic 1 like
A_24_P408603	RAB39B	-2.02038	8.67E-06	0.000189	-6.50445	Down	RAB39B, member RAS oncogene family
A_24_P225604	DNAJC10	-1.42899	9.4E-06	0.000202	-6.45908	Down	DnaJ heat shock protein family (Hsp40) member C10
A_24_P147461	SERPINB8	-1.52799	9.59E-06	0.000205	-6.44734	Down	serpin family B member 8
A_23_P18078	RARRES1	-1.4362	9.75E-06	0.000208	-6.43832	Down	retinoic acid receptor responder 1

A_24_P372134	TMEM140	-1.6791	1.09E-05	0.000228	-6.37452	Down	transmembrane protein 140
A_33_P3396766	#N/A	-2.49657	1.14E-05	0.000235	-6.35006	Down	NA
A_23_P255444	DAPP1	-1.56954	1.17E-05	0.00024	-6.33726	Down	dual adaptor of phosphotyrosine and 3-phosphoinositides 1
A_24_P298013	GTPBP2	-1.94526	1.18E-05	0.000241	-6.33143	Down	GTP binding protein 2
A_23_P43476	VLDLR	-1.45039	1.3E-05	0.00026	-6.27853	Down	very low density lipoprotein receptor
A_23_P76386	SLC6A12	-1.4905	1.34E-05	0.000268	-6.25926	Down	solute carrier family 6 member 12
A_33_P3277110	SLC5A3	-1.67593	1.5E-05	0.000294	-6.19875	Down	solute carrier family 5 member 3
A_33_P3259153	TINCR	-1.74236	1.72E-05	0.000327	-6.12407	Down	tissue differentiation-inducing non-protein coding RNA
A_24_P227927	IL21R	-1.44578	1.79E-05	0.000338	-6.10066	Down	interleukin 21 receptor
A_23_P103110	MAFF	-1.7854	2.39E-05	0.000424	-5.94447	Down	MAF bZIP transcription factor F
A_24_P226970	ZNF365	-1.70199	2.43E-05	0.00043	-5.9354	Down	zinc finger protein 365
A_19_P00319254	LINC01564	-2.70649	2.51E-05	0.000441	-5.91883	Down	long intergenic non-protein coding RNA 1564
A_24_P161018	PARP14	-1.36814	2.84E-05	0.000485	-5.85198	Down	poly(ADP-ribose) polymerase family member 14
A_23_P304897	BDKRB2	-1.52621	3.2E-05	0.000536	-5.78755	Down	bradykinin receptor B2
A_23_P143016	ARID5A	-1.51975	3.27E-05	0.000546	-5.77655	Down	AT-rich interaction domain 5A
A_19_P00330911	#N/A	-1.71457	3.38E-05	0.000561	-5.75812	Down	NA
A_23_P34915	ATF3	-1.91244	3.62E-05	0.00059	-5.72287	Down	activating transcription factor 3
A_23_P2414	SPX	-1.36048	3.72E-05	0.000604	-5.70697	Down	spexin hormone
A_33_P3269636	SBSN	-1.43039	4.04E-05	0.000647	-5.66336	Down	suprabasin
A_33_P3364869	NAMPT	-1.83974	4.69E-05	0.000734	-5.58478	Down	nicotinamidephosphoribosyltransferase
A_23_P90172	PPP1R15A	-1.45125	4.85E-05	0.000753	-5.56792	Down	protein phosphatase 1 regulatory subunit 15A
A_23_P66241	MT1M	-2.68548	4.99E-05	0.000767	-5.55294	Down	metallothionein 1M
A_24_P333326	MIA2	-1.6267	5.07E-05	0.000778	-5.54414	Down	melanoma inhibitory activity 2
A_19_P00320275	LINC00632	-1.51527	5.85E-05	0.000876	-5.4693	Down	long intergenic non-protein coding RNA 632

NA- not annotated

Table S2. The enriched pathway terms of the up-regulated differentially expressed genes

BIOCYC

Pathway ID	Pathway Name	P-value	FDR B&H	FDR B&Y	Bonferroni	Gene Count	Gene
1108775	lysine degradation II (pipecolate pathway)	2.29E-03	8.67E-02	3.83E-01	1.05E-01	2	CRYM,ALDH7A1
782392	pyrimidine deoxyribonucleosides salvage	3.77E-03	8.67E-02	3.83E-01	1.73E-01	2	TYMS,TK1
782390	superpathway of pyrimidine deoxyribonucleotides de novo biosynthesis	7.88E-03	1.21E-01	5.34E-01	3.62E-01	3	RRM2,TYMS,CTPS1
545335	4-aminobutyrate degradation	2.03E-02	1.51E-01	6.65E-01	9.31E-01	1	ABAT
545280	dermatansulfate biosynthesis	2.27E-02	1.51E-01	6.65E-01	1.00E+00	2	CHST14,XYLT1
782380	pyrimidine deoxyribonucleotides de novo biosynthesis	3.47E-02	1.51E-01	6.65E-01	1.00E+00	2	RRM2,TYMS
782381	pyrimidine deoxyribonucleotides biosynthesis from CTP	3.47E-02	1.51E-01	6.65E-01	1.00E+00	2	RRM2,TYMS
782391	superpathway of pyrimidine deoxyribonucleoside salvage	3.92E-02	1.51E-01	6.65E-01	1.00E+00	2	TYMS,TK1
142362	lysine degradation I	3.93E-02	1.51E-01	6.65E-01	1.00E+00	1	ALDH7A1

142417	(saccharopine pathway) propionyl-CoA degradation	3.93E-02	1.51E-01	6.65E-01	1.00E+00	1	MCEE
**KEGG**							
83039	DNA replication	7.29E-17	1.39E-14	8.08E-14	1.39E-14	15	MCM2,MCM3,MCM4,MCM5,MCM6,MCM7,POLA2,POLA1,POLD1,POLE2,RFC2,RFC3,RFC5,LIG1,PCNA
83054	Cell cycle	3.19E-15	3.03E-13	1.77E-12	6.06E-13	22	CDKN2C,ORC6,MCM2,MCM3,MCM4,MCM5,MCM6,MCM7,CDC7,CDC45,ESPL1,BUB1B,E2F1,E2F2,CCNA2,PKMYT1,TGFB2,CCNE1,CCNE2,CDC25A,PCNA,CDK2
83045	Mismatch repair	8.13E-09	5.15E-07	3.00E-06	1.54E-06	8	POLD1,RFC2,RFC3,RFC5,LIG1,MSH6,EXO1,PCNA
83046	Homologous recombination	1.16E-06	5.52E-05	3.22E-04	2.21E-04	8	RAD54L,POLD1,XRCC3,BARD1,BLM,BRCA1,RAD51,RBBP8
83044	Nucleotide excision repair	3.55E-05	1.35E-03	7.86E-03	6.75E-03	7	POLD1,POLE2,RFC2,RFC3,RFC5,LIG1,PCNA
1393107	Hippo signaling pathway -multiple species	2.37E-04	7.52E-03	4.38E-02	4.51E-02	5	WTIP,RASSF2,TEAD1,TEAD4,AJUBA
83043	Base excision repair	4.45E-04	1.21E-02	7.03E-02	8.45E-02	5	UNG,POLD1,POLE2,LIG1,PCNA
377262	Fanconianemia pathway	7.38E-04	1.75E-02	1.02E-01	1.40E-01	6	FANCA,RMI2,BLM,BRCA1,RAD51,FANCI
82946	Pyrimidine metabolism	1.14E-03	2.41E-02	1.40E-01	2.17E-01	8	RRM2,POLA2,TYMS,POLA1,POLD1,POLE2,CTPS1,TK1
83111	Prostate cancer	1.69E-03	3.20E-02	1.87E-01	3.20E-01	7	PDGFB,FGFR2,E2F1,E2F2,CCNE1,CCNE2,CDK2
**Pathway Interaction Database**							
137934	E2F transcription factor network	4.33E-14	2.69E-12	1.27E-11	2.69E-12	17	CDKN2C,MCM3,RRM2,TYMS,POLA1,MYBL2,BRCA1,DHFR,RBBP8,E2F1,E2F2,CCNA2,CCNE1,TK1,CCNE2,CDC25A,CDK2
137959	BARD1 signaling events	6.42E-08	1.99E-06	9.37E-06	3.98E-06	8	FANCA,BARD1,BRCA1,RAD51,RBBP8,CCNE1,PCNA,CDK2
138007	PLK1 signaling events	2.28E-05	4.70E-04	2.22E-03	1.41E-03	7	NDC80,SPC24,ERCC6L,FBXO5,BUB1B,CENPU,KIF20A
138080	Aurora B signaling	9.80E-04	1.52E-02	7.15E-02	6.07E-02	5	NDC80,KIF23,NCAPG,KIF20A,AURKB
169349	Validated transcriptional targets of AP1 family members Fra1 and Fra2	4.39E-03	5.45E-02	2.57E-01	2.72E-01	4	CCL2,DCN,IVL,CCNA2
137969	Signaling events mediated by PRL	1.02E-02	1.05E-01	4.96E-01	6.32E-01	3	CCNA2,CCNE1,CDK2
137925	Aurora A signaling	2.30E-02	1.93E-01	9.07E-01	1.00E+00	3	BRCA1,AJUBA,AURKB
137916	Regulation of retinoblastoma protein	2.48E-02	1.93E-01	9.07E-01	1.00E+00	4	CCNA2,TGFB2,CCNE1,CDK2
**REACTOME**							
1269741	Cell Cycle	7.27E-41	4.49E-38	3.15E-37	4.49E-38	77	CDKN2C,ORC6,HJURP,SPC25,MCM2,MCM3,MCM4,MCM5,MCM6,MCM7,MND1,MCM10,RRM2,POLA2,HIST2H3A,DSN1,SYCE2,CDC7,CDC45,TYMS,HAUS8,HIST1H2BF,FBXL18,NDC80,HIST1H3B,HIST1H4D,CENPM,PSMC3IP,GINS3,KIF23,POLA1,POLD1,POLE2,GTSE1,RMI2,GINS4,SPC24,NDC1,GINS2,ESPL1,CDCA5,MYBL2,KNTC1,ERCC6L,BARD1,GINS1,BLM,NCAPG,FBXO5,BRCA1,DHFR,BUB1B,CDT1,RAD51,HIST1H3F,RBB

							P8,CENPU,E2F1,E2F2,RFC2,RFC3,RFC5,ZWINT,CCNA2,ESCO2,PKMYT1,KIF20A,CCNE1,LIG1,LMNB1,CCNE2,EXO1,CDC25A,AJUBA,PCNA,CDK2,AURKB
1269763	Cell Cycle, Mitotic	1.36E-35	4.22E-33	2.95E-32	8.43E-33	66	CDKN2C,ORC6,SPC25,MCM2,MCM3,MCM4,MCM5,MCM6,MCM7,MCM10,RRM2,POLA2,HIST2H3A,DSN1,CDC7,CDC45,TYMS,HAUS8,HIST1H2BF,FBXL18,NDC80,HIST1H3B,HIST1H4D,CENPM,GINS3,KIF23,POLA1,POLD1,POLE2,GTSE1,GINS4,SPC24,NDC1,GINS2,ESPL1,CDCA5,MYBL2,KNTC1,ERCC6L,GINS1,NCAPG,FBXO5,DHFR,BUB1B,CDT1,HIST1H3F,CENPU,E2F1,E2F2,RFC2,RFC3,RFC5,ZWINT,CCNA2,ESCO2,PKMYT1,KIF20A,CCNE1,LIG1,LMNB1,CCNE2,CDC25A,AJUBA,PCNA,CDK2,AURKB
1269784	DNA strand elongation	6.20E-25	1.28E-22	8.94E-22	3.83E-22	19	MCM2,MCM3,MCM4,MCM5,MCM6,MCM7,POLA2,CDC45,GINS3,POLA1,POLD1,GINS4,GINS2,GINS1,RFC2,RFC3,RFC5,LIG1,PCNA
1269875	DNA Replication	1.58E-23	2.44E-21	1.71E-20	9.77E-21	28	ORC6,MCM2,MCM3,MCM4,MCM5,MCM6,MCM7,MCM10,POLA2,CDC7,CDC45,GINS3,POLA1,POLD1,POLE2,GINS4,GINS2,GINS1,CDT1,E2F1,E2F2,RFC2,RFC3,RFC5,CCNA2,LIG1,PCNA,CDK2
1269777	S Phase	1.73E-22	2.14E-20	1.50E-19	1.07E-19	29	ORC6,MCM2,MCM3,MCM4,MCM5,MCM6,MCM7,POLA2,CDC45,GINS3,POLA1,POLD1,POLE2,GINS4,GINS2,CDCA5,GINS1,CDT1,RFC2,RFC3,RFC5,CCNA2,ESCO2,CCNE1,LIG1,CCNE2,CDC25A,PCNA,CDK2
1269764	Mitotic G1-G1/S phases	3.99E-20	4.11E-18	2.88E-17	2.46E-17	28	CDKN2C,ORC6,MCM2,MCM3,MCM4,MCM5,MCM6,MCM7,MCM10,RRM2,POLA2,CDC7,CDC45,TYMS,POLA1,POLE2,MYBL2,FBXO5,DHFR,CDT1,E2F1,E2F2,CCNA2,CCNE1,CCNE2,CDC25A,PCNA,CDK2
1269779	Synthesis of DNA	2.01E-19	1.77E-17	1.24E-16	1.24E-16	24	ORC6,MCM2,MCM3,MCM4,MCM5,MCM6,MCM7,POLA2,CDC45,GINS3,POLA1,POLD1,POLE2,GINS4,GINS2,GINS1,CDT1,RFC2,RFC3,RFC5,CCNA2,LIG1,PCNA,CDK2
1269768	G1/S Transition	7.94E-19	6.13E-17	4.30E-16	4.91E-16	25	ORC6,MCM2,MCM3,MCM4,MCM5,MCM6,MCM7,MCM10,RRM2,POLA2,CDC7,CDC45,TYMS,POLA1,POLE2,FBXO5,DHFR,CDT1,E2F1,CCNA2,CCNE1,CCNE2,CDC25A,PCNA,CDK2
1269785	Unwinding of DNA	1.77E-18	1.22E-16	8.52E-16	1.09E-15	11	MCM2,MCM3,MCM4,MCM5,MCM6,MCM7,CDC45,GINS3,GINS4,GINS2,GINS1
1269773	Activation of the pre-replicative complex	7.95E-18	4.92E-16	3.44E-15	4.92E-15	15	ORC6,MCM2,MCM3,MCM4,MCM5,MCM6,MCM7,MCM10,POLA2,CDC7,CDC45,POLA1,P

							OLE2,CDT1,CDK2
			**GenMAPP**				
MAP00240	Pyrimidine metabolism	2.15E-04	6.68E-03	2.69E-02	6.68E-03	6	RRM2,TYMS,POLA1,POLD1,CTPS1,TK1
MAP00670	One carbon pool by folate	1.92E-02	2.35E-01	9.44E-01	5.94E-01	2	TYMS,DHFR
MAP00790	Folate biosynthesis	2.27E-02	2.35E-01	9.44E-01	7.04E-01	2	TYMS,DHFR
			**MSigDB C2 BIOCARTA (v6.0)**				
M6682	CDK Regulation of DNA Replication	3.00E-13	2.64E-11	1.34E-10	2.64E-11	10	ORC6,MCM2,MCM3,MCM4,MCM5,MCM6,MCM7,CDT1,CCNE1,CDK2
M3686	Expression of cyclins regulates progression through the cell cycle by activating cyclin-dependent kinases.	8.77E-08	3.86E-06	1.95E-05	7.71E-06	6	E2F1,E2F2,CCNA2,CCNE1,CCNE2,CDK2
M648	Cell Cycle: G1/S Check Point	1.50E-05	4.40E-04	2.23E-03	1.32E-03	6	DHFR,E2F1,TGFB2,CCNE1,CDC25A,CDK2
M17770	Cyclins and Cell Cycle Regulation	7.41E-05	1.63E-03	8.25E-03	6.52E-03	5	CDKN2C,E2F1,CCNE1,CDC25A,CDK2
M14863	p53 Signaling Pathway	2.28E-04	4.01E-03	2.03E-02	2.01E-02	4	E2F1,CCNE1,PCNA,CDK2
M6031	E2F1 Destruction Pathway	8.35E-04	1.23E-02	6.20E-02	7.35E-02	3	E2F1,CCNE1,CDK2
M17977	Regulation of p27 Phosphorylation during Cell Cycle Progression	1.90E-03	2.39E-02	1.21E-01	1.68E-01	3	E2F1,CCNE1,CDK2
M1529	Cdk2, 4, and 6 bind cyclin D in G1, while cdk2/cyclin E promotes the G1/S transition.	2.94E-03	3.24E-02	1.64E-01	2.59E-01	3	E2F1,E2F2,CDK2
M10628	ATM Signaling Pathway	6.85E-03	6.70E-02	3.39E-01	6.03E-01	3	BRCA1,RAD51,RBBP8
M5884	Ensemble of genes encoding core extracellular matrix including ECM glycoproteins, collagens and proteoglycans	8.85E-03	7.79E-02	3.94E-01	7.79E-01	12	EFEMP1,COL12A1,ABI3BP,COL27A1,IGFBP4,IGFBP5,CYR61,CTGF,SMOC2,DCN,WISP2,THBS1
			**PantherDB**				
P00017	DNA replication	2.28E-04	1.00E-02	4.39E-02	1.00E-02	4	HIST1H3B,POLA1,POLD1,PCNA
P02742	Tetrahydrofolate biosynthesis	3.77E-03	8.18E-02	3.58E-01	1.66E-01	2	TYMS,DHFR
P02743	Formyltetrahydroformate biosynthesis	5.58E-03	8.18E-02	3.58E-01	2.46E-01	2	TYMS,DHFR
P00059	p53 pathway	2.03E-02	2.23E-01	9.75E-01	8.92E-01	5	RRM2,GTSE1,CCNE1,THBS1,CDK2
P02739	De novo pyrimidine deoxyribonucleotide biosynthesis	3.05E-02	2.68E-01	1.00E+00	1.00E+00	2	RRM2,TYMS
P02726	Aminobutyrate degradation	3.93E-02	2.88E-01	1.00E+00	1.00E+00	1	ABAT
P04372	5-Hydroxytryptamine degredation	4.87E-02	2.91E-01	1.00E+00	1.00E+00	2	ALDH1A3,ALDH7A1
			**Pathway Ontology**				
PW:0000662	mismatch repair pathway	2.46E-08	1.03E-06	4.47E-06	1.03E-06	8	POLD1,RFC2,RFC3,RFC5,LIG1,MSH6,EXO1,PCNA
PW:0000130	nucleotide excision repair	4.00E-04	8.40E-03	3.64E-02	1.68E-02	2	POLD1,LIG1
PW:0000243	vascular endothelial growth factor signaling	1.15E-02	1.06E-01	4.58E-01	4.82E-01	3	CCL2,FLT4,EGLN3
PW:0000490	transforming growth factor-beta Smad dependent signaling	1.43E-02	1.06E-01	4.58E-01	6.02E-01	3	DCN,RUNX1,TGFB2
PW:0000244	Angiotensin II signaling	1.59E-02	1.06E-01	4.58E-01	6.67E-01	2	CCL2,OLR1
PW:0000095	G1/S DNA damage	2.03E-02	1.06E-01	4.58E-01	8.50E-01	1	CCNE1

	checkpoint						
PW:0000350	altered vascular endothelial growth factor signaling	2.03E-02	1.06E-01	4.58E-01	8.50E-01	1	FLT4
PW:0000032	pyrimidine metabolic	2.27E-02	1.06E-01	4.58E-01	9.53E-01	2	TYMS,DHFR
PW:0000238	insulin-like growth factor signaling	2.27E-02	1.06E-01	4.58E-01	9.53E-01	2	IGFBP4,IGFBP5
PW:0000189	folate mediated one-carbon metabolic	3.47E-02	1.27E-01	5.49E-01	1.00E+00	2	TYMS,DHFR
				SMPDB			
SMP00046	Pyrimidine Metabolism	1.29E-02	2.72E-01	1.00E+00	7.20E-01	3	TYMS,CTPS1,TK1
SMP00351	GABA-Transaminase Deficiency	2.03E-02	2.72E-01	1.00E+00	1.00E+00	1	ABAT
SMP00033	Methionine Metabolism	3.47E-02	2.72E-01	1.00E+00	1.00E+00	2	MARS,MSRB3
SMP00380	Nimodipine Pathway	4.87E-02	2.72E-01	1.00E+00	1.00E+00	2	MYL9,CALD1
SMP00378	Isradipine Pathway	4.87E-02	2.72E-01	1.00E+00	1.00E+00	2	MYL9,CALD1
SMP00382	Nitrendipine Pathway	4.87E-02	2.72E-01	1.00E+00	1.00E+00	2	MYL9,CALD1
SMP00377	Felodipine Pathway	4.87E-02	2.72E-01	1.00E+00	1.00E+00	2	MYL9,CALD1
SMP00379	Nifedipine Pathway	4.87E-02	2.72E-01	1.00E+00	1.00E+00	2	MYL9,CALD1
SMP00376	Amlodipine Pathway	4.87E-02	2.72E-01	1.00E+00	1.00E+00	2	MYL9,CALD1
SMP00381	Nisoldipine Pathway	4.87E-02	2.72E-01	1.00E+00	1.00E+00	2	MYL9,CALD1

Kyoto Encyclopedia of Genes and Genomes (KEGG), GenMAPP(MAP) and Pathway Ontology (PW)

Table S3: The enriched pathway terms of the down-regulated differentially expressed genes

Pathway ID	Pathway Name	P-value	FDR B&H	FDR B&Y	Bonferroni	Gene Count	Gene
				BIOCYC			
142269	superpathway of cholesterol biosynthesis	2.51E-14	1.45E-12	6.75E-12	1.45E-12	12	HMGCR,HMGCS1,MSMO1,FDPS,HSD17B7,IDI1,EBP,MVD,SQLE,CYP51A1,DHCR7,LSS
142266	cholesterol biosynthesis II (via 24,25-dihydrolanosterol)	2.67E-09	3.87E-08	1.80E-07	1.55E-07	7	MSMO1,HSD17B7,EBP,SQLE,CYP51A1,DHCR7,LSS
142267	cholesterol biosynthesis I	2.67E-09	3.87E-08	1.80E-07	1.55E-07	7	MSMO1,HSD17B7,EBP,SQLE,CYP51A1,DHCR7,LSS
142268	cholesterol biosynthesis III (via desmosterol)	2.67E-09	3.87E-08	1.80E-07	1.55E-07	7	MSMO1,HSD17B7,EBP,SQLE,CYP51A1,DHCR7,LSS
545288	superpathway of geranylgeranyldiphosphate biosynthesis I (via mevalonate)	2.84E-06	3.30E-05	1.53E-04	1.65E-04	5	HMGCR,HMGCS1,FDPS,IDI1, MVD
142207	mevalonate pathway	3.70E-05	3.58E-04	1.66E-03	2.15E-03	4	HMGCR,HMGCS1,IDI1,MVD
545317	zymosterol biosynthesis	1.78E-04	1.47E-03	6.85E-03	1.03E-02	3	MSMO1,HSD17B7,CYP51A1
545337	putrescine degradation III	4.83E-04	3.50E-03	1.63E-02	2.80E-02	3	SAT1,ALDH3B2,SAT2
142319	N-acetylglucosamine degradation I	1.32E-03	8.48E-03	3.94E-02	7.63E-02	2	GNPDA1,AMDHD2
142221	trans, trans-farnesyldiphosphate biosynthesis	2.59E-03	1.25E-02	5.82E-02	1.50E-01	2	FDPS,IDI1
				KEGG			
212237	Mineral absorption	6.84E-09	1.63E-06	9.85E-06	1.63E-06	11	HMOX1,STEAP1,MT1A,MT1B,MT1E,MT1F,MT1H,MT1M,MT1X,MT2A,FTL
413390	Cholesterol biosynthesis, squalene 2,3-epoxide => cholesterol	3.55E-08	4.22E-06	2.56E-05	8.45E-06	6	MSMO1,HSD17B7,EBP,CYP51A1,DHCR7,LSS

83051	Cytokine-cytokine receptor interaction	6.21E-08	4.93E-06	2.98E-05	1.48E-05	22	CCL5,CCL20,VEGFA,VEGFB,IL23A,IL21R,IL3RA,IL6R,IL15,TNFSF10,TNFSF9,TNFRSF10B,CCR10,INHBE,CXCL1,CXCL2,CXCL3,CD70,LTB,IFNL2,IFNL3,IFNL1
82937	Steroid biosynthesis	1.06E-07	6.32E-06	3.83E-05	2.53E-05	7	MSMO1,HSD17B7,EBP,SQLE,CYP51A1,DHCR7,LSS
1510435	Ferroptosis	1.53E-06	7.27E-05	4.40E-04	3.63E-04	8	HMOX1,SLC7A11,ACSL1,SAT1,FTL,PRNP,MAP1LC3B,SAT2
122191	NOD-like receptor signaling pathway	1.47E-05	5.75E-04	3.48E-03	3.51E-03	14	IRF9,CCL5,BIRC3,CYBA,GBP2,CASP4,CASP5,OAS1,OAS2,CXCL1,CXCL2,CXCL3,NAMPT,TNFAIP3
217173	Influenza A	1.80E-05	5.75E-04	3.48E-03	4.28E-03	14	IRF9,CIITA,FDPS,CCL5,EIF2AK3,HSPA6,ICAM1,RSAD2,MX1,TNFSF10,TNFRSF10B,IFIH1,OAS1,OAS2
200309	Rheumatoid arthritis	1.93E-05	5.75E-04	3.48E-03	4.60E-03	10	ACP5,CCL5,CCL20,VEGFA,ICAM1,IL23A,IL15,CXCL1,TLR2,LTB
213306	Measles	2.63E-05	6.97E-04	4.22E-03	6.27E-03	12	IRF9,EIF2AK3,HSPA6,BBC3,MX1,TNFSF10,TNFRSF10B,IFIH1,OAS1,OAS2,TLR2,TNFAIP3
413387	C5 isoprenoid biosynthesis, mevalonate pathway	3.70E-05	8.80E-04	5.33E-03	8.80E-03	4	HMGCR,HMGCS1,IDI1,MVD

Pathway Interaction Database

138006	ATF-2 transcription factor network	1.48E-04	1.20E-02	5.96E-02	1.20E-02	7	PLAU,IL23A,ATF3,HRK,GADD45A,DDIT3,PPARGC1A
137939	Direct p53 effectors	1.94E-03	7.84E-02	3.90E-01	1.57E-01	9	NDRG1,DDIT4,GDF15,ATF3,BBC3,TNFRSF10B,GADD45A,JMY,BDKRB2
137924	VEGFR1 specific signals	3.05E-03	8.23E-02	4.10E-01	2.47E-01	4	SHC2,VEGFA,VEGFB,NOS3
138045	HIF-1-alpha transcription factor network	1.19E-02	2.42E-01	1.00E+00	9.67E-01	5	HK2,HMOX1,NDRG1,VEGFA,BHLHE41
169349	Validated transcriptional targets of AP1 family members Fra1 and Fra2	3.45E-02	4.21E-01	1.00E+00	1.00E+00	3	HMOX1,PLAU,NOS3
138012	Signaling mediated by p38-alpha and p38-beta	3.71E-02	4.21E-01	1.00E+00	1.00E+00	3	CEBPB,DDIT3,PPARGC1A
169353	Validated targets of C-MYC transcriptional repression	3.98E-02	4.21E-01	1.00E+00	1.00E+00	4	NDRG1,CCL5,GADD45A,DDIT3
137940	Signaling events mediated by VEGFR1 and VEGFR2	4.40E-02	4.21E-01	1.00E+00	1.00E+00	4	SHC2,VEGFA,VEGFB,NOS3
138046	Syndecan-1-mediated signaling events	4.91E-02	4.21E-01	1.00E+00	1.00E+00	2	CCL5,SDCBP

REACTOME

1270037	Cholesterol biosynthesis	1.33E-14	6.76E-12	4.60E-11	6.76E-12	12	HMGCR,HMGCS1,MSMO1,FDPS,HSD17B7,IDI1,EBP,MVD,SQLE,CYP51A1,DHCR7,LSS
1339157	Response to metal ions	5.57E-12	9.46E-10	6.44E-09	2.84E-09	8	MT1A,MT1B,MT1E,MT1F,MT1H,MT1M,MT1X,MT2A
1339158	Metallothioneins bind metals	5.57E-12	9.46E-10	6.44E-09	2.84E-09	8	MT1A,MT1B,MT1E,MT1F,MT1H,MT1M,MT1X,MT2A
1269310	Cytokine Signaling in Immune system	7.89E-11	1.00E-08	6.84E-08	4.02E-08	46	IL32,UBE2L6,HMOX1,IRF9,SHC2,CIITA,IFI30,CCL5,CCL20,VE

							GFA,ICAM1,IFITM1,BIRC3,IFIT2,IL23A,IFIT1,IFIT3,RSAD2,MT2A,ISG15,OASL,IRS2,IL3RA,IL6R,MX1,IL15,TNFSF9,GBP2,IRF1,ISG20,GAB2,STX3,SQSTM1,OAS1,OAS2,RELB,LCN2,CXCL1,CXCL2,CD70,RORA,RORC,LTB,IFNL2,IFNL3,IFNL1
1269312	Interferon alpha/beta signaling	1.37E-10	1.39E-08	9.48E-08	6.96E-08	14	IRF9,IFITM1,IFIT2,IFIT1,IFIT3,RSAD2,ISG15,OASL,MX1,GBP2,IRF1,ISG20,OAS1,OAS2
1270038	Regulation of cholesterol biosynthesis by SREBP (SREBF)	1.20E-09	1.02E-07	6.95E-07	6.13E-07	12	HMGCR,HMGCS1,FDPS,ELOVL6,IDI1,MVD,INSIG1,SQLE,CYP51A1,SEC24D,DHCR7,LSS
1270039	Activation of gene expression by SREBF (SREBP)	1.21E-08	8.80E-07	6.00E-06	6.16E-06	10	HMGCR,HMGCS1,FDPS,ELOVL6,IDI1,MVD,SQLE,CYP51A1,DHCR7,LSS
1270001	Metabolism of lipids and lipoproteins	2.11E-08	1.34E-06	9.15E-06	1.08E-05	43	HMGCR,HMGCS1,FABP6,ACSL1,MSMO1,FDPS,ELOVL6,ALDH3B2,VLDLR,HSD17B7,STARD4,PLA2G3,PLEKHA4,IDH1,IDI1,NR1D1,PLA2G4C,TRIB3,PLPP2,PLPP3,EBP,AKR1C3,PLD6,CERS1,MVD,CYP1A2,INSIG1,CYP27A1,SQLE,CYP51A1,AKR1C1,LPIN1,PPARGC1A,SEC24D,NEU1,SLC27A1,DHCR7,GM2A,GPAT3,LDLR,LSS,RORA,HSD17B14
1269311	Interferon Signaling	5.32E-08	3.01E-06	2.05E-05	2.71E-05	19	UBE2L6,IRF9,CIITA,IFI30,ICAM1,IFITM1,IFIT2,IFIT1,IFIT3,RSAD2,MT2A,ISG15,OASL,MX1,GBP2,IRF1,ISG20,OAS1,OAS2
1269314	Interferon gamma signaling	2.83E-05	1.44E-03	9.83E-03	1.44E-02	10	IRF9,CIITA,IFI30,ICAM1,MT2A,OASL,GBP2,IRF1,OAS1,OAS2
**GenMAPP**							
MAP00100	Sterol biosynthesis	1.64E-08	5.26E-07	2.13E-06	5.26E-07	6	HMGCR,FDPS,IDI1,MVD,SQLE,LSS
MAP00361	gamma Hexachlorocyclohexane degradation	2.27E-02	3.17E-01	1.00E+00	7.27E-01	3	ACP5,CYP1A2,CYP51A1
MAP00530	Aminosugars metabolism	2.97E-02	3.17E-01	1.00E+00	9.52E-01	2	HK2,RENBP
**MSigDB C2 BIOCARTA (v6.0)**							
M5883	Genes encoding secreted soluble factors	3.77E-04	3.31E-02	1.68E-01	3.31E-02	18	S100A14,S100A3,S100P,CCL5,FGF13,CCL20,VEGFA,VEGFB,GDF15,IL23A,IL15,TNFSF10,TNFSF9,INHBE,CXCL1,CXCL2,CXCL3,LTB
M4383	Actions of Nitric Oxide in the Heart	3.91E-03	1.72E-01	8.70E-01	3.44E-01	4	PDE2A,VEGFA,BDKRB2,NOS3
M5885	Ensemble of genes encoding ECM-associated proteins including ECM-affilaited proteins, ECM regulators and secreted factors	2.88E-02	5.61E-01	1.00E+00	1.00E+00	24	LGALS9C,SERPINA3,S100A14,F7,S100A3,S100P,SERPINB8,CCL5,PLAU,FGF13,CCL20,VEGFA,VEGFB,GDF15,IL23A,LGALSL,IL15,TNFSF10,TNFSF9,INHBE,CXCL1,CXCL2,CXCL3,LTB
M14339	Oxidative Stress Induced Gene Expression Via Nrf2	2.97E-02	5.61E-01	1.00E+00	1.00E+00	2	MAFG,MAFF
M14971	Induction of apoptosis through DR3 and DR4/5 Death Receptors	3.19E-02	5.61E-01	1.00E+00	1.00E+00	3	BIRC3,TNFSF10,TNFRSF10B

M13324	Hypoxia-Inducible Factor in the Cardiovascular System	3.90E-02	5.71E-01	1.00E+00	1.00E+00	2	VEGFA,NOS3
			**PantherDB**				
P00014	Cholesterol biosynthesis	3.55E-08	1.78E-06	7.99E-06	1.78E-06	6	HMGCR,HMGCS1,FDPS,MVD,SQLE,LSS
P00006	Apoptosis signaling pathway	5.76E-05	1.44E-03	6.48E-03	2.88E-03	10	HSPA5,HSPA6,BIRC3,IGF2R,ATF3,TNFSF10,TNFRSF10B,BCL2L11,RELB,LTB
P00036	Interleukin signaling pathway	3.30E-03	5.50E-02	2.47E-01	1.65E-01	7	IL23A,IRS2,MAPK6,IL3RA,IL6R,IL15,NOS3
P02756	N-acetylglucosamine metabolism	6.31E-03	7.88E-02	3.55E-01	3.15E-01	2	GNPDA1,AMDHD2
P00056	VEGF signaling pathway	3.39E-02	3.39E-01	1.00E+00	1.00E+00	4	SHC2,VEGFA,CRYAB,NOS3
			**Pathway Ontology**				
PW:0000454	cholesterol biosynthetic	5.63E-13	3.21E-11	1.49E-10	3.21E-11	10	HMGCR,HMGCS1,FDPS,IDI1,EBP,MVD,SQLE,CYP51A1,DHCR7,LSS
PW:0000248	steroid biosynthetic	6.69E-09	1.91E-07	8.82E-07	3.81E-07	6	FDPS,IDI1,MVD,SQLE,DHCR7,LSS
PW:0000184	terpenoid biosynthetic	3.67E-05	6.97E-04	3.23E-03	2.09E-03	3	FDPS,IDI1,SQLE
PW:0000021	hypertension	2.59E-03	3.70E-02	1.71E-01	1.48E-01	2	HMOX1,SOD2
PW:0000111	gamma-hexachlorocyclohexane degradation	1.79E-02	1.85E-01	8.56E-01	1.00E+00	2	ACP5,CYP1A2
PW:0000413	heme catabolic	2.16E-02	1.85E-01	8.56E-01	1.00E+00	1	HMOX1
PW:0000104	intrinsic apoptotic	2.27E-02	1.85E-01	8.56E-01	1.00E+00	3	BBC3,HRK,BCL2L11
PW:0000492	renin-angiotensin system signaling	3.90E-02	1.93E-01	8.91E-01	1.00E+00	2	IGF2R,RENBP
PW:0000065	butanoate metabolic	3.90E-02	1.93E-01	8.91E-01	1.00E+00	2	HMGCS1,GAD1
PW:0000353	altered vascular endothelial growth factor signaling involving proteins affecting its expression	4.18E-02	1.93E-01	8.91E-01	1.00E+00	1	VEGFA
			**SMPDB**				
SMP00023	Steroid Biosynthesis	8.88E-14	8.08E-12	4.12E-11	8.08E-12	11	HMGCR,HMGCS1,MSMO1,FDPS,HSD17B7,IDI1,EBP,MVD,SQLE,CYP51A1,LSS
SMP00112	Risedronate Pathway	6.97E-08	5.29E-07	2.69E-06	6.35E-06	6	HMGCR,HMGCS1,FDPS,MVD,SQLE,LSS
SMP00099	Lovastatin Pathway	6.97E-08	5.29E-07	2.69E-06	6.35E-06	6	HMGCR,HMGCS1,FDPS,MVD,SQLE,LSS
SMP00119	Fluvastatin Pathway	6.97E-08	5.29E-07	2.69E-06	6.35E-06	6	HMGCR,HMGCS1,FDPS,MVD,SQLE,LSS
SMP00117	Pamidronate Pathway	6.97E-08	5.29E-07	2.69E-06	6.35E-06	6	HMGCR,HMGCS1,FDPS,MVD,SQLE,LSS
SMP00131	Atorvastatin Pathway	6.97E-08	5.29E-07	2.69E-06	6.35E-06	6	HMGCR,HMGCS1,FDPS,MVD,SQLE,LSS
SMP00079	Ibandronate Pathway	6.97E-08	5.29E-07	2.69E-06	6.35E-06	6	HMGCR,HMGCS1,FDPS,MVD,SQLE,LSS
SMP00089	Pravastatin Pathway	6.97E-08	5.29E-07	2.69E-06	6.35E-06	6	HMGCR,HMGCS1,FDPS,MVD,SQLE,LSS
SMP00092	Rosuvastatin Pathway	6.97E-08	5.29E-07	2.69E-06	6.35E-06	6	HMGCR,HMGCS1,FDPS,MVD,SQLE,LSS
SMP00107	Zoledronate Pathway	6.97E-08	5.29E-07	2.69E-06	6.35E-06	6	HMGCR,HMGCS1,FDPS,MVD,SQLE,LSS

Kyoto Encyclopedia of Genes and Genomes (KEGG), GenMAPP(MAP) and Pathway Ontology (PW)

Table S4: The enriched GO terms of the up-regulated differentially expressed genes

GO ID	CATEGORY	GO Name	P Value	FDR B&H	FDR B&Y	Bonferroni	Gene Count	Gene
GO:0007049	BP	cell cycle	4.66E-31	2.13E-27	1.92E-26	2.13E-27	107	NUSAP1,CDKN2C,CHAF1B,PDGFB,ORC6,HJURP,SPC25,MCM2,MCM3,MCM4,MCM5,MCM6,MCM7,MND1,MCM10,RRM2,POLA2,TRIP13,DSN1,SYCE2,CDC7,CDC45,FANCA,TYMS,HAUS8,AUNIP,NDC80,CENPM,ANLN,RECQL4,MKI67,ASPM,PSMC3IP,FGFR2,DSCC1,RAD54L,FLNA,KIF23,POLA1,POLE2,GTSE1,DTL,ID3,DCLRE1B,XRCC3,SIPA1,SPAG5,EGFL6,SPC24,NDC1,UHRF1,GINS2,CTGF,ESPL1,CDCA5,MYBL2,STARD13,CDCA3,KNTC1,ERCC6L,PCLAF,INHBA,RASSF2,PBK,BARD1,GINS1,BLM,NEDD9,NCAPG,FBXO5,BRCA1,KIF15,DHFR,BUB1B,BOP1,CDT1,KIF11,KIFC1,RAD51,TCF19,RBBP8,CENPU,E2F1,E2F2,CIT,E2F8,ZWINT,CCNA2,ESCO2,PKMYT1,KIF20A,TGFB2,CCNE1,LIG1,MSH6,THBS1,LMNB1,CCNE2,FANCI,EXO1,SKA3,CDC25A,AJUBA,PCNA,CDK2,AURKB,HELLS
GO:0006260	BP	DNA replication	9.16E-30	2.09E-26	1.88E-25	4.18E-26	49	CHAF1B,PDGFB,ORC6,MCM2,MCM3,MCM4,MCM5,MCM6,MCM7,MCM10,RRM2,POLA2,CDC7,CDC45,RECQL4,DSCC1,GINS3,POLA1,POLD1,POLE2,DTL,RMI2,ID3,GINS4,GINS2,PCLAF,BARD1,GINS1,BLM,BRCA1,NFIB,TONSL,CDT1,RAD51,MMS22L,RBBP8,RFC2,RFC3,RFC5,E2F8,ESCO2,CCNE1,LIG1,CCNE2,EXO1,CDC25A,PCNA,CDK2,AURKB
GO:0022402	BP	cell cycle process	2.72E-29	4.14E-26	3.72E-25	1.24E-25	92	NUSAP1,CDKN2C,PDGFB,ORC6,SPC25,MCM2,MCM3,MCM4,MCM5,MCM6,MCM7,MCM10,RRM2,POLA2,TRIP13,DSN1,SYCE2,CDC7,CDC45,FANCA,TYMS,HAUS8,AUNIP,NDC80,CENPM,ANLN,RECQL4,MKI67,ASPM,FGFR2,DSCC1,RAD54L,FLNA,KIF23,POLA1,POLE2,GTSE1,DTL,DCLRE1B,XRCC3,SPAG5,SPC24,NDC1,GINS2,CTGF,ESPL1,CDCA5,MYBL2,CDCA3,KNTC1,ERCC6L,INHBA,PBK,BARD1,GINS1,BLM,NEDD9,NCAPG,FBXO5,BRCA1,KIF15,DHFR,BUB1B,CDT1,KIF11,KIFC1,RAD51,TCF19,RBBP8,CENPU,E2F1,CIT,E

								2F8,ZWINT,CCNA2,PKMYT1,KIF20A,TGFB2,CCNE1,LIG1,MSH6,THBS1,LMNB1,CCNE2,FANCI,SKA3,CDC25A,AJUBA,PCNA,CDK2,AURKB,HELLS
GO:0006261	BP	DNA-dependent DNA replication	2.32E-28	2.64E-25	2.38E-24	1.06E-24	33	ORC6,MCM2,MCM3,MCM4,MCM5,MCM6,MCM7,MCM10,POLA2,CDC7,CDC45,DSCC1,GINS3,POLA1,POLD1,POLE2,GINS4,GINS2,GINS1,BLM,TONSL,CDT1,RAD51,MMS22L,RFC2,RFC3,RFC5,E2F8,CCNE1,LIG1,CCNE2,PCNA,CDK2
GO:1903047	BP	mitotic cell cycle process	2.43E-27	2.22E-24	2.00E-23	1.11E-23	73	NUSAP1,CDKN2C,PDGFB,ORC6,SPC25,MCM2,MCM3,MCM4,MCM5,MCM6,MCM7,MCM10,RRM2,POLA2,DSN1,CDC7,CDC45,TYMS,HAUS8,NDC80,ANLN,ASPM,FGFR2,DSCC1,FLNA,KIF23,POLA1,POLE2,GTSE1,XRCC3,SPAG5,SPC24,NDC1,GINS2,ESPL1,CDCA5,MYBL2,CDCA3,KNTC1,ERCC6L,INHBA,PBK,GINS1,BLM,NEDD9,NCAPG,FBXO5,KIF15,DHFR,BUB1B,CDT1,KIF11,KIFC1,TCF19,RBBP8,E2F1,CIT,ZWINT,CCNA2,PKMYT1,KIF20A,CCNE1,LIG1,LMNB1,CCNE2,FANCI,SKA3,CDC25A,AJUBA,PCNA,CDK2,AURKB,HELLS
GO:0000278	BP	mitotic cell cycle	1.01E-25	7.68E-23	6.91E-22	4.61E-22	74	NUSAP1,CDKN2C,PDGFB,ORC6,SPC25,MCM2,MCM3,MCM4,MCM5,MCM6,MCM7,MCM10,RRM2,POLA2,DSN1,CDC7,CDC45,TYMS,HAUS8,NDC80,ANLN,ASPM,FGFR2,DSCC1,FLNA,KIF23,POLA1,POLE2,GTSE1,XRCC3,SPAG5,SPC24,NDC1,GINS2,ESPL1,CDCA5,MYBL2,CDCA3,KNTC1,ERCC6L,INHBA,PBK,GINS1,BLM,NEDD9,NCAPG,FBXO5,KIF15,DHFR,BUB1B,CDT1,KIF11,KIFC1,TCF19,RBBP8,E2F1,CIT,E2F8,ZWINT,CCNA2,PKMYT1,KIF20A,CCNE1,LIG1,LMNB1,CCNE2,FANCI,SKA3,CDC25A,AJUBA,PCNA,CDK2,AURKB,HELLS
GO:0006270	BP	DNA replication initiation	8.03E-23	4.88E-20	4.39E-19	3.67E-19	18	MCM2,MCM3,MCM4,MCM5,MCM6,MCM7,MCM10,POLA2,CDC7,CDC45,POLA1,POLE2,GINS4,GINS2,CDT1,CCNE1,CCNE2,CDK2
GO:0006259	BP	DNA metabolic process	8.55E-23	4.88E-20	4.39E-19	3.90E-19	69	CHAF1B,PDGFB,ORC6,MCM2,MCM3,MCM4,MCM5,MCM6,MCM7,MND1,MCM10,RRM2,POLA2,TRIP13,CDC7,CDC45,FANCA,TYMS,HIST1H4D,RECQL4,MKI67,PSMC3IP,UNG,DSCC1,RAD54L,GINS3,POLA1,POLD1,POL

								E2,DTL,RMI2,ID3,GINS4,DCLRE1B,XRCC3,RAD51AP1,IGFBP4,UHRF1,GINS2,CTGF,CDCA5,PCLAF,BARD1,GINS1,BLM,BRCA1,NFIB,TONSL,CDT1,RAD51,MMS22L,RBBP8,RFC2,RFC3,RFC5,E2F8,ESCO2,CCNE1,LIG1,MSH6,TK1,CCNE2,FANCI,EXO1,CDC25A,PCNA,CDK2,AURKB,HELLS
GO:0051276	BP	chromosome organization	4.47E-19	2.27E-16	2.04E-15	2.04E-15	70	NUSAP1,CHAF1B,HJURP,SPC25,MCM2,MCM3,MCM4,MCM5,MCM6,MCM7,POLA2,TRIP13,HIST2H3A,DSN1,SYCE2,CDC45,HIST1H2AI,HIST1H2AL,HIST1H2BF,SATB1,NDC80,HIST1H3B,HIST1H4D,CENPM,RECQL4,DSCC1,RAD54L,KIF23,POLA1,POLD1,POLE2,GINS4,DCLRE1B,XRCC3,ATAD2,SPAG5,SPC24,NDC1,ASF1B,UHRF1,GINS2,ESPL1,CDCA5,KDM4B,KNTC1,PHF19,ERCC6L,GINS1,BLM,NCAPG,BRCA1,BUB1B,KIFC1,RAD51,HIST1H3F,CENPU,RFC2,RFC3,RFC5,CIT,ZWINT,LIG1,MSH6,WDHD1,HIST1H1D,HIST1H1B,PCNA,CDK2,AURKB,HELLS
GO:0048285	BP	organelle fission	9.31E-19	4.25E-16	3.83E-15	4.25E-15	50	NUSAP1,PDGFB,ACOX1,SPC25,TRIP13,DSN1,SYCE2,FANCA,HAUS8,NDC80,ANLN,MKI67,ASPM,FGFR2,DSCC1,RAD54L,FLNA,KIF23,XRCC3,SPAG5,SPC24,NDC1,ESPL1,CDCA5,MYBL2,CDCA3,KNTC1,ERCC6L,PBK,DCN,NEDD9,NCAPG,FBXO5,KIF15,BUB1B,KIF11,KIFC1,RAD51,RBBP8,DNM1L,CIT,ZWINT,CCNA2,PKMYT1,MSH6,SKA3,CDC25A,CDK2,AURKB,HELLS
GO:0005694	CC	chromosome	1.98E-28	9.20E-26	6.18E-25	9.20E-26	74	NUSAP1,CHAF1B,ORC6,HJURP,SPC25,MCM2,MCM3,MCM4,MCM5,MCM6,MCM7,MCM10,POLA2,HIST2H3A,DSN1,SYCE2,CDC45,HIST1H2AI,HIST1H2AL,HIST1H2BF,SATB1,NDC80,HIST1H3B,HIST1H4D,CENPM,RECQL4,MKI67,DSCC1,FLNA,POLA1,POLD1,POLE2,DTL,GINS4,DCLRE1B,XRCC3,SPAG5,SPC24,ASF1B,UHRF1,GINS2,CDCA5,KDM4B,MYBL2,KNTC1,ERCC6L,GINS1,BLM,NCAPG,BRCA1,ANKRD2,TONSL,BUB1B,RAD51,HIST1H3F,TCF4,MMS22L,RBBP8,CENPU,E2F1,RFC2,RFC3,RFC5,ZWINT,ESCO2,MSH6,WDHD1,HIST1H1D,HIST1H1B,SKA3,PCNA,CDK2,AURKB,HELLS

GO:0044427	CC	chromosomal part	1.30E-27	3.03E-25	2.04E-24	6.06E-25	69	CHAF1B,ORC6,HJURP,SPC25,MCM2,MCM3,MCM4,MCM5,MCM6,MCM7,MCM10,POLA2,HIST2H3A,DSN1,SYCE2,CDC45,HIST1H2AI,HIST1H2AL,HIST1H2BF,SATB1,NDC80,HIST1H3B,HIST1H4D,CENPM,MKI67,DSCC1,FLNA,POLA1,POLD1,POLE2,GINS4,DCLRE1B,XRCC3,SPAG5,SPC24,ASF1B,UHRF1,GINS2,CDCA5,KDM4B,MYBL2,KNTC1,ERCC6L,GINS1,BLM,NCAPG,ANKRD2,TONSL,BUB1B,RAD51,HIST1H3F,TCF4,MMS22L,CENPU,E2F1,RFC2,RFC3,RFC5,ZWINT,ESCO2,MSH6,WDHD1,HIST1H1D,HIST1H1B,SKA3,PCNA,CDK2,AURKB,HELLS
GO:0098687	CC	chromosomal region	8.24E-19	1.28E-16	8.58E-16	3.83E-16	37	HJURP,SPC25,MCM2,MCM3,MCM4,MCM5,MCM6,MCM7,DSN1,NDC80,HIST1H3B,HIST1H4D,CENPM,MKI67,DSCC1,POLD1,DCLRE1B,XRCC3,SPAG5,SPC24,CDCA5,KDM4B,KNTC1,ERCC6L,BLM,BUB1B,RAD51,HIST1H3F,CENPU,ZWINT,ESCO2,WDHD1,SKA3,PCNA,CDK2,AURKB,HELLS
GO:0044454	CC	nuclear chromosome part	1.44E-18	1.68E-16	1.13E-15	6.70E-16	45	CHAF1B,ORC6,MCM2,MCM3,MCM4,MCM5,MCM6,MCM7,MCM10,POLA2,SYCE2,CDC45,HIST1H2AI,HIST1H2AL,HIST1H2BF,SATB1,NDC80,HIST1H3B,HIST1H4D,POLA1,POLD1,POLE2,GINS4,DCLRE1B,XRCC3,ASF1B,UHRF1,GINS2,CDCA5,KDM4B,GINS1,BLM,TONSL,BUB1B,RAD51,HIST1H3F,TCF4,MMS22L,E2F1,ESCO2,MSH6,HIST1H1D,HIST1H1B,PCNA,AURKB
GO:0000228	CC	nuclear chromosome	3.70E-18	3.45E-16	2.32E-15	1.72E-15	46	CHAF1B,ORC6,MCM2,MCM3,MCM4,MCM5,MCM6,MCM7,MCM10,POLA2,SYCE2,CDC45,HIST1H2AI,HIST1H2AL,HIST1H2BF,SATB1,NDC80,HIST1H3B,HIST1H4D,POLA1,POLD1,POLE2,GINS4,DCLRE1B,XRCC3,ASF1B,UHRF1,GINS2,CDCA5,KDM4B,GINS1,BLM,BRCA1,TONSL,BUB1B,RAD51,HIST1H3F,TCF4,MMS22L,E2F1,ESCO2,MSH6,HIST1H1D,HIST1H1B,PCNA,AURKB
GO:0005657	CC	replication fork	6.61E-17	5.12E-15	3.44E-14	3.07E-14	18	MCM3,MCM10,POLA2,CDC45,POLA1,POLD1,POLE2,GINS4,XRCC3,UHRF1,GINS2,BLM,TONSL,MMS22L,RFC2,RFC3,RFC5,PCNA
GO:0042555	CC	MCM complex	1.29E-12	8.56E-11	5.76E-10	5.99E-10	8	MCM2,MCM3,MCM4,MCM5,MCM6,MCM7,TONSL,

								MMS22L
GO:0043596	CC	nuclear replication fork	3.45E-12	2.00E-10	1.35E-09	1.60E-09	12	MCM3,MCM10,POLA2,CDC45,POLA1,POLD1,POLE2,GINS4,GINS2,TONSL,MMS22L,PCNA
GO:0032993	CC	protein-DNA complex	1.03E-11	5.32E-10	3.57E-09	4.79E-09	21	MCM3,POLA2,HIST2H3A,CDC45,HIST1H2AI,HIST1H2AL,HIST1H2BF,HIST1H3B,HIST1H4D,POLA1,POLD1,POLE2,GINS4,GINS2,GINS1,TONSL,HIST1H3F,TEAD4,HIST1H1D,HIST1H1B,PCNA
GO:0000775	CC	chromosome, centromeric region	1.27E-11	5.90E-10	3.97E-09	5.90E-09	21	HJURP,SPC25,DSN1,NDC80,CENPM,MKI67,DSCC1,SPAG5,SPC24,CDCA5,KDM4B,KNTC1,ERCC6L,BUB1B,CENPU,ZWINT,ESCO2,WDHD1,SKA3,AURKB,HELLS
GO:0003678	MF	DNA helicase activity	1.53E-09	1.25E-06	9.13E-06	1.25E-06	12	MCM2,MCM3,MCM4,MCM5,MCM6,MCM7,CDC45,RECQL4,GINS4,GINS2,GINS1,BLM
GO:0043138	MF	3'-5' DNA helicase activity	4.85E-08	1.99E-05	1.45E-04	3.98E-05	6	CDC45,RECQL4,GINS4,GINS2,GINS1,BLM
GO:0004386	MF	helicase activity	5.00E-07	1.37E-04	9.96E-04	4.10E-04	15	MCM2,MCM3,MCM4,MCM5,MCM6,MCM7,CDC45,RECQL4,RAD54L,GINS4,GINS2,ERCC6L,GINS1,BLM,HELLS
GO:0008094	MF	DNA-dependent ATPase activity	8.45E-07	1.73E-04	1.26E-03	6.93E-04	11	MCM4,MCM6,MCM7,RECQL4,DSCC1,XRCC3,BLM,RAD51,RFC2,RFC3,RFC5
GO:0043142	MF	single-stranded DNA-dependent ATPase activity	3.13E-06	5.13E-04	3.74E-03	2.56E-03	5	DSCC1,RAD51,RFC2,RFC3,RFC5
GO:0003682	MF	chromatin binding	6.42E-06	8.77E-04	6.39E-03	5.26E-03	25	CHAF1B,MCM5,CDC45,LHX2,SATB1,POLA1,POLD1,CITED1,ATAD2,UHRF1,GRHL3,CDCA5,PCLAF,BRCA1,ANKRD2,RAD51,TCF4,MSH6,WDHD1,HIST1H1D,HIST1H1B,EXO1,AJUBA,PCNA,HELLS
GO:0033170	MF	protein-DNA loading ATPase activity	1.18E-05	1.08E-03	7.86E-03	9.70E-03	4	DSCC1,RFC2,RFC3,RFC5
GO:0003688	MF	DNA replication origin binding	1.18E-05	1.08E-03	7.86E-03	9.70E-03	4	MCM2,MCM5,MCM10,CDC45
GO:0003689	MF	DNA clamp loader activity	1.18E-05	1.08E-03	7.86E-03	9.70E-03	4	DSCC1,RFC2,RFC3,RFC5
GO:0044877	MF	protein-containing	1.39E-05	1.14E-03	8.30E-03	1.14E-02	53	NUSAP1,CHAF1B,PDGFB,MAP6D1,MCM5,CDC45,S100A8,S100A9,LHX2,SATB1,PIP

		complex binding						,FLNA,KIF23,ABI3BP,POLA1,POLD1,ID1,CITED1,ATAD2,SPAG5,EGFL6,MB21D2,CYR61,UHRF1,CTGF,GRHL3,CDCA5,PCLAF,DCN,WISP2,BRCA1,KIF15,ANKRD2,BOP1,KIF5C,KIF11,KIFC1,RAD51,TCF4,KRT8,DOK7,KIF20A,CCNE1,MSH6,THBS1,WDHD1,HIST1H1D,HIST1H1B,EXO1,AJUBA,PCNA,CDK2,HELLS

Biological Process(BP), Cellular Component(CC) and Molecular Functions (MF)

Table S5. The enriched GO terms of the down-regulated differentially expressed genes

GO ID	CATEGORY	GO Name	P Value	FDR B&H	FDR B&Y	Bonferroni	Gene Count	Gene
GO:0034097	BP	response to cytokine	6.06E-14	2.90E-10	2.62E-09	2.90E-10	49	SERPINA3,PDE2A,CEBPB,ACP5,KLF4,ACSL1,IRF9,CIITA,IFI30,CCL5,CCL20,HSPA5,VLDLR,ICAM1,IFITM1,BIRC3,IFIT2,IFIT1,IFIT3,MT1X,RSAD2,MT2A,ISG15,IL21R,OASL,LAMP3,IL3RA,IL6R,MX1,CYBA,GBP2,IRF1,ISG20,TNFRSF10B,CEACAM1,CREBRF,PPARGC1A,SLC27A1,CCR10,OAS1,OAS2,RELB,CXCL1,CXCL2,CXCL3,CD70,RORA,LTB,TNFAIP3
GO:0071345	BP	cellular response to cytokine stimulus	3.65E-13	8.71E-10	7.88E-09	1.74E-09	44	PDE2A,CEBPB,ACSL1,IRF9,CIITA,IFI30,CCL5,CCL20,HSPA5,VLDLR,ICAM1,IFITM1,BIRC3,IFIT2,IFIT1,IFIT3,MT1X,RSAD2,MT2A,ISG15,IL21R,OASL,IL3RA,IL6R,MX1,CYBA,GBP2,IRF1,ISG20,TNFRSF10B,CEACAM1,CREBRF,PPARGC1A,SLC27A1,CCR10,OAS1,OAS2,CXCL1,CXCL2,CXCL3,CD70,RORA,LTB,TNFAIP3
GO:0008202	BP	steroid metabolic process	1.89E-12	2.97E-09	2.69E-08	9.04E-09	28	HMGCR,HMGCS1,FABP6,MSMO1,FDPS,APOL2,VLDLR,HSD17B7,STARD4,IDI1,NR1D1,RDH16,EBP,AKR1C3,MVD,CYP1A2,INSIG1,CYP27A1,SQLE,CYP51A1,AKR1C1,PPARGC1A,DHCR7,LDLR,LSS,RORA,RORC,HSD17B14
GO:1902653	BP	secondary alcohol biosynthetic process	3.11E-12	2.97E-09	2.69E-08	1.49E-08	13	HMGCR,HMGCS1,MSMO1,FDPS,HSD17B7,IDI1,EBP,MVD,INSIG1,SQLE,CYP51A1,DHCR7,LSS
GO:0006695	BP	cholesterol	3.11E-12	2.97E-09	2.69E-08	1.49E-08	13	HMGCR,HMGCS1,MS

		biosynthetic process						MO1,FDPS,HSD17B7,IDI1,EBP,MVD,INSIG1,SQLE,CYP51A1,DHCR7,LSS
GO:0009607	BP	response to biotic stimulus	4.64E-12	3.69E-09	3.34E-08	2.21E-08	52	CEBPB,ACP5,S100A14,LY96,IRF9,CCL5,PLAU,CCL20,EIF2AK3,HSPA5,VLDLR,DDIT4,DDX60,ICAM1,IFI44,IFITM1,BIRC3,NR1D1,IFIT2,IL23A,IFIT1,IFIT3,RSAD2,ISG15,OASL,ULBP1,IL6R,MX1,CYBA,CYP1A2,IL15,GBP2,IRF1,ISG20,TNFRSF10B,BDKRB1,DDIT3,IFIH1,PPARGC1A,NOS3,BCL2L11,OAS1,OAS2,LCN2,CXCL1,CXCL2,CXCL3,TLR2,TNFAIP3,IFNL2,IFNL3,IFNL1
GO:0006955	BP	immune response	6.52E-12	4.39E-09	3.97E-08	3.11E-08	67	IL32,CEBPB,S100A14,HMOX1,LY96,IRF9,CIITA,IFI30,CCL5,CCL20,VEGFA,DDX60,PLA2G3,ICAM1,ICAM4,IFITM1,BIRC3,ST6GAL1,NR1D1,IFIT2,IL23A,IFIT1,IFIT3,RSAD2,MT2A,ISG15,OASL,LAMP3,ULBP1,IL6R,PRNP,MX1,CYBA,IL15,TNFSF10,TNFSF9,GBP2,IRF1,ISG20,TNFRSF10B,NCR3LG1,BDKRB2,CFB,GAB2,CEACAM1,IFIH1,GADD45G,CCR10,RAB17,CASP4,OAS1,OAS2,RELB,LCN2,CXCL1,CXCL2,CXCL3,BTN3A1,TLR2,CD70,RORA,RORC,LTB,TNFAIP3,IFNL2,IFNL3,IFNL1
GO:0006915	BP	apoptotic process	7.35E-12	4.39E-09	3.97E-08	3.51E-08	76	HK2,CEBPB,DRAM1,S100A14,HMGCR,HMOX1,LY96,PPP1R15A,KLF4,NDRG1,DNAJC10,GGCT,UCN,CCL5,FGF13,ALDOC,EIF2AK3,HSPA5,GULP1,VEGFA,VEGFB,DDIT4,RNF152,HYOU1,GDF15,ERO1A,ICAM1,BIRC3,ST6GAL1,IFIT2,IFIT3,CRYAB,IGF2R,TRIB3,LAMP3,AKR1C3,FLCN,ITPRIP,ATF3,IRS2,BBC3,HERPUD1,IL6R,PRNP,MX1,SOD2,HRK,TNFSF10,TNFSF9,UNC5B,PEG10,IRF1,TNFRSF10B,GADD45A,JMY,BDKRB2,DDIT3,RRAGC,CEACAM1,PPARGC1A,PSMG2,GADD45G,SQSTM1,BEX2,NOS3,INHBE,BCL2L11,HIP1R,CASP4,CASP5,HS1BP3,LCN2,TLR2,CD70,RORC,TNFAIP3

GO:0016126	BP	sterol biosynthetic process	1.14E-11	6.05E-09	5.48E-08	5.45E-08	13	HMGCR,HMGCS1,MSMO1,FDPS,HSD17B7,IDI1,EBP,MVD,INSIG1,SQLE,CYP51A1,DHCR7,LSS
GO:0019221	BP	cytokine-mediated signaling pathway	1.34E-11	6.41E-09	5.80E-08	6.41E-08	36	ACSL1,IRF9,CIITA,IFI30,CCL5,CCL20,ICAM1,IFITM1,BIRC3,IFIT2,IFIT1,IFIT3,RSAD2,MT2A,ISG15,IL21R,OASL,IL3RA,IL6R,MX1,GBP2,IRF1,ISG20,TNFRSF10B,CEACAM1,CREBRF,SLC27A1,CCR10,OAS1,OAS2,CXCL1,CXCL2,CXCL3,CD70,LTB,TNFAIP3
GO:0005783	CC	endoplasmic reticulum	2.38E-08	1.04E-05	6.93E-05	1.04E-05	62	PDE2A,CLGN,HMGCR,HMOX1,DNAJB9,PPP1R15A,SLC7A11,F7,ACSL1,DNAJC10,MSMO1,ELOVL6,SDR16C5,APOL2,EIF2AK3,HSPA5,UVRAG,SDCBP,HSD17B7,COL16A1,HYOU1,ERO1B,STARD4,ERO1A,ST6GAL1,IFIT2,RSAD2,PLA2G4C,RDH16,MIA2,PLPP3,EBP,ULBP1,CERS1,BBC3,HERPUD1,PRNP,MX1,CYBA,CYP1A2,SLC36A1,INSIG1,SQLE,CYP51A1,RNF43,BDKRB1,LPIN1,SEC24D,NFE2L1,SQSTM1,SLC27A1,DHCR7,TMEM97,NUCB2,GPAT3,CASP4,OAS1,OAS2,HS1BP3,FADS3,LSS,SLC33A1
GO:0042175	CC	nuclear outer membrane-endoplasmic reticulum membrane network	1.43E-06	2.15E-04	1.44E-03	6.25E-04	40	CLGN,HMGCR,HMOX1,DNAJB9,PPP1R15A,ACSL1,MSMO1,ELOVL6,SDR16C5,APOL2,EIF2AK3,HSPA5,SDCBP,HSD17B7,ERO1B,ERO1A,RSAD2,PLA2G4C,RDH16,PLPP3,EBP,CERS1,HERPUD1,MX1,CYBA,CYP1A2,INSIG1,SQLE,CYP51A1,RNF43,LPIN1,SEC24D,NFE2L1,DHCR7,NUCB2,GPAT3,CASP4,FADS3,LSS,SLC33A1
GO:0044432	CC	endoplasmic reticulum part	1.66E-06	2.15E-04	1.44E-03	7.25E-04	44	CLGN,HMGCR,HMOX1,DNAJB9,PPP1R15A,F7,ACSL1,DNAJC10,MSMO1,ELOVL6,SDR16C5,APOL2,EIF2AK3,HSPA5,SDCBP,HSD17B7,COL16A1,HYOU1,ERO1B,ERO1A,RSAD2,PLA2G4C,RDH16,MIA2,PLPP3,EBP,CERS1,HERPUD1,MX1,CYBA,CYP1A2,INSIG1,SQLE,CYP51A1,RNF43,LPIN1,SEC24D,NFE2L1,DHCR7,GPAT3,CASP4,FAD

								S3,LSS,SLC33A1
GO:0005789	CC	endoplasmic reticulum membrane	2.17E-06	2.15E-04	1.44E-03	9.52E-04	39	CLGN,HMGCR,HMOX1,DNAJB9,PPP1R15A,ACSL1,MSMO1,ELOVL6,SDR16C5,APOL2,EIF2AK3,HSPA5,SDCBP,HSD17B7,ERO1B,ERO1A,RSAD2,PLA2G4C,RDH16,PLPP3,EBP,CERS1,HERPUD1,MX1,CYBA,CYP1A2,INSIG1,SQLE,CYP51A1,RNF43,LPIN1,SEC24D,NFE2L1,DHCR7,GPAT3,CASP4,FADS3,LSS,SLC33A1
GO:0005615	CC	extracellular space	3.03E-06	2.15E-04	1.44E-03	1.33E-03	50	SERPINA3,IL32,ACP5,HMOX1,LY96,F7,SERPINB8,CCL5,PLAU,CCL20,HSPA6,SDCBP,VEGFA,VEGFB,VLDLR,GDF15,PLA2G3,ICAM1,ICAM4,IL23A,TSKU,SCGB1D1,RETN,IGF2R,ASIP,IL6R,IL15,PRSS8,TNFSF10,TNFSF9,CFB,CEACAM1,CMTM8,VSTM1,INHBE,NUCB2,KCP,LCN2,CXCL1,CXCL2,CXCL3,CBR3,LDLR,SPX,NAMPT,CD70,LTB,IFNL2,IFNL3,IFNL1
GO:0005764	CC	lysosome	3.44E-06	2.15E-04	1.44E-03	1.51E-03	26	DRAM1,ACP5,GLMP,RRAGD,CLCN5,CLCN7,TPP1,HPS1,IFI30,UVRAG,RNF152,PLA2G3,IGF2R,LAMP3,FLCN,BBC3,SLC36A1,RILP,RRAGC,NEU1,STX3,SQSTM1,GM2A,TMEM97,LDLR,TNFAIP3
GO:0000323	CC	lytic vacuole	3.44E-06	2.15E-04	1.44E-03	1.51E-03	26	DRAM1,ACP5,GLMP,RRAGD,CLCN5,CLCN7,TPP1,HPS1,IFI30,UVRAG,RNF152,PLA2G3,IGF2R,LAMP3,FLCN,BBC3,SLC36A1,RILP,RRAGC,NEU1,STX3,SQSTM1,GM2A,TMEM97,LDLR,TNFAIP3
GO:0031968	CC	organelle outer membrane	4.42E-05	2.42E-03	1.61E-02	1.94E-02	13	HK2,PPP1R15A,ACSL1,VAT1,KMO,RSAD2,PLD6,BBC3,PRNP,LPIN1,DHCR7,BCL2L11,NUCB2
GO:0019867	CC	outer membrane	6.35E-05	3.09E-03	2.06E-02	2.78E-02	13	HK2,PPP1R15A,ACSL1,VAT1,KMO,RSAD2,PLD6,BBC3,PRNP,LPIN1,DHCR7,BCL2L11,NUCB2
GO:0005773	CC	vacuole	2.17E-04	8.83E-03	5.88E-02	9.52E-02	39	DRAM1,ACP5,LY96,GLMP,SAMD9L,NDRG1,RRAGD,CLCN5,CLCN7,TPP1,HPS1,IFI30,UVRAG,STEAP1,RNF152,PLA2G3,IGF2R,LAMP3,FLCN,BBC3,CYBA,IL15,SLC36A1,RILP,BDKRB2,DDIT3,RRAGC,NEU1,STX3,RAB24,SQSTM1,CCDC154

								,GM2A,TMEM97,MAP1LC3B,RAB29,RAB17,LDLR,TNFAIP3
GO:0005125	MF	cytokine activity	4.41E-10	4.44E-07	3.33E-06	4.44E-07	21	IL32,CCL5,CCL20,VEGFA,GDF15,IL23A,IL15,TNFSF10,TNFSF9,CMTM8,VSTM1,INHBE,CXCL1,CXCL2,CXCL3,NAMPT,CD70,LTB,IFNL2,IFNL3,IFNL1
GO:0005126	MF	cytokine receptor binding	4.86E-08	2.45E-05	1.84E-04	4.90E-05	21	S100A14,CCL5,CCL20,SDCBP,VEGFA,VEGFB,HSD17B7,GDF15,IL23A,IL6R,IL15,TNFSF10,TNFSF9,INHBE,NUCB2,CXCL1,CXCL2,CXCL3,CD70,LTB,IFNL1
GO:0016709	MF	oxidoreductase activity, acting on paired donors, with incorporation or reduction of molecular oxygen, NAD(P)H as one donor, and incorporation of one atom of oxygen	6.00E-07	2.02E-04	1.51E-03	6.05E-04	8	MSMO1,KMO,AKR1C3,CYP27A1,SQLE,CYP51A1,AKR1C1,NOS3
GO:0005102	MF	signaling receptor binding	2.67E-06	6.73E-04	5.04E-03	2.69E-03	54	IL32,CEBPB,ADM2,S100A14,F7,S100P,SHC2,UCN,CCL5,FGF13,GPNMB,CCL20,APOL2,SDCBP,VEGFA,VEGFB,HSD17B7,COL16A1,GDF15,ICAM1,ICAM4,IDH1,IL23A,RETN,PLPP3,ASIP,OASL,ULBP1,IRS2,IL6R,IL15,TNFSF10,TNFSF9,RNF43,BDKRB2,GAB2,CMTM8,LPIN1,PPARGC1A,SQSTM1,VSTM1,INHBE,NUCB2,CXCL1,CXCL2,CXCL3,SPX,NAMPT,TLR2,CD70,LTB,IFNL2,IFNL3,IFNL1
GO:0016491	MF	oxidoreductase activity	1.01E-05	2.04E-03	1.53E-02	1.02E-02	31	HMGCR,HMOX1,DNAJC10,MSMO1,IFI30,ALDH3B2,SDR16C5,STEAP1,VAT1,HSD17B7,ERO1B,ERO1A,IDH1,KMO,RDH16,AKR1C3,SOD2,CYBA,CYP1A2,SRXN1,CYP27A1,SQLE,CYP51A1,AKR1C1,ALDH1L2,DHCR7,SESN2,NOS3,CBR3,FADS3,HSD17B14
GO:0001730	MF	2'-5'-oligoadenylate synthetase activity	2.14E-05	3.60E-03	2.70E-02	2.16E-02	3	OASL,OAS1,OAS2
GO:0004497	MF	monooxygenase activity	5.41E-05	7.78E-03	5.83E-02	5.45E-02	9	MSMO1,KMO,AKR1C3,CYP1A2,CYP27A1,SQLE,CYP51A1,AKR1C1,NOS3
GO:0070191	MF	methionine-R-	1.04E-04	1.32E-02	9.86E-02	1.05E-01	3	DNAJC10,ERO1B,ERO1

		sulfoxidereductas e activity						A
GO:0033743	MF	peptide-methionine (R)-S-oxide reductase activity	1.80E-04	1.74E-02	1.30E-01	1.82E-01	3	DNAJC10,ERO1B,ERO1A
GO:0070566	MF	adenylyltransfera se activity	1.93E-04	1.74E-02	1.30E-01	1.94E-01	5	UAP1L1,OASL,OAS1,OAS2,FICD

Biological Process(BP), Cellular Component(CC) and Molecular Functions (MF)

Table S6. Topology table for up and down regulated genes

Regulation	Gene	Degree	Betweenness	Stress	Closeness	Clustering Coefficient
Up	OLR1	14	6.97E-04	1446932	0.267779	0
Up	CHST8	33	0.005764	3197290	0.269557	0
Up	KLF8	14	7.79E-04	755870	0.295879	0
Up	CFH	23	0.003746	2465838	0.291245	0
Up	XAGE2	11	7.71E-04	617610	0.268648	0
Up	TCF4	238	0.04852	35161704	0.342369	0.001551
Up	HIST1H1B	74	5.05E-03	3973868	0.341265	0.017031
Up	ASPM	97	0.012646	12481606	0.319094	4.30E-04
Up	H19	1	0.00E+00	0	0.195629	0
Up	KCNB1	19	0.004046	2375640	0.281404	0
Up	COL12A1	10	4.37E-04	187734	0.273509	0
Up	DIAPH3	36	0.003611	4114808	0.307832	0
Up	ZNF185	4	3.89E-05	20532	0.290891	0.166667
Up	HSPB11	20	0.002876	1629338	0.270042	0
Up	HIST1H3F	454	6.42E-02	47668690	0.388514	0.006878
Up	CDC45	40	0.001654	1011394	0.341157	0.139744
Up	ACTA1	212	3.14E-02	22994438	0.358556	0.005332
Up	SFXN2	18	0.002236	3511728	0.266474	0
Up	KLF17	1	0.00E+00	0	1	0
Up	GLDC	23	0.002688	2619952	0.295729	0
Up	THBS1	58	9.61E-03	3401560	0.305443	0.011039
Up	ASPM	97	0.012646	12481606	0.319094	4.30E-04
Up	CDH18	7	4.85E-04	485330	0.257357	0
Up	DIAPH3	36	0.003611	4114808	0.307832	0
Up	PLXNA1	29	4.24E-03	3505792	0.277401	0
Up	CALD1	43	1.73E-03	1937352	0.314753	0.003322
Up	KRT4	27	1.37E-03	1272494	0.299643	0
Up	HELLS	49	4.62E-03	4012396	0.32025	0.002551
Up	PCNA	326	4.58E-02	35744384	0.383414	0.012556
Up	DISP1	3	3.35E-04	236830	0.228153	0
Up	CCNE2	17	4.19E-04	280320	0.327714	0.095588
Up	HR	15	7.46E-04	734500	0.293031	0
Up	HIST1H3B	454	6.42E-02	47668690	0.388514	0.006878
Up	SULF2	18	3.45E-03	2091034	0.278598	0
Up	VSIG10L	1	0.00E+00	0	0.244451	0
Up	EXO1	31	1.79E-03	1238350	0.319505	0.032258

Up	FSTL1	39	8.75E-03	4902390	0.283406	0
Up	TMEM79	18	3.47E-03	2079642	0.245371	0
Up	SYNPO	159	9.71E-03	6818146	0.342951	0.022736
Up	HIST2H3A	116	1.10E-02	7238882	0.356496	0.013793
Up	ZWINT	98	1.20E-02	6063532	0.323785	0.01299
Up	PCNA	326	4.58E-02	35744384	0.383414	0.012556
Up	HIST1H2BF	262	4.13E-02	22206672	0.373387	0.004185
Up	CDK2	737	1.32E-01	84519756	0.417518	0.005023
Up	TNNI2	20	2.84E-03	2026358	0.281392	0
Up	SLC12A4	28	4.72E-03	4477100	0.274323	0.00E+00
Up	UHRF1	70	4.95E-03	3499650	0.338597	0.028973
Up	ATAD2	22	6.74E-04	514362	0.32359	0.082251
Up	KIF23	64	6.11E-03	4207438	0.33089	0.011634
Up	HAUS8	53	4.85E-03	4406746	0.29949	0
Up	TYMS	57	5.89E-03	6846108	0.301293	0
Up	MCM10	61	5.90E-03	2777822	0.346672	0.046756
Up	CARD9	80	9.65E-03	7148952	0.303575	0.002331
Up	CYR61	13	3.03E-04	205540	0.299921	0
Up	HIST1H2AI	55	4.27E-03	2327038	0.337552	0.022222
Up	HIST1H4D	323	4.07E-02	35951898	0.385843	0.01213
Up	CDC25A	88	9.58E-03	6265808	0.341861	0.012325
Up	WDHD1	49	6.59E-03	3798682	0.322088	0.015306
Up	MKI67	63	5.20E-03	3343804	0.348545	0.023497
Up	KRT8	82	7.70E-03	10821362	0.323185	0
Up	ESCO2	19	5.29E-04	306544	0.30452	0.023392
Up	RAD51	126	1.63E-02	9135232	0.347531	0.015998
Up	RFC2	68	4.17E-03	3117994	0.335325	0.044337
Up	MYBL2	54	3.98E-03	1840776	0.336076	0.039216
Up	GINS2	23	1.37E-03	656898	0.29596	0.075099
Up	TGFB2	20	2.67E-03	1160224	0.2893	0.019608
Up	COL12A1	10	4.37E-04	187734	0.273509	0
Up	VTCN1	2	1.00E+00	2	1	0
Up	GPR20	3	6.20E-04	362154	0.253635	0
Up	FRMD3	11	1.93E-03	1205382	0.257213	0
Up	AMOTL2	79	1.03E-02	4780216	0.318449	0.006152
Up	CCNE2	17	4.19E-04	280320	0.327714	0.095588
Up	MYCBP	44	5.38E-03	2872166	0.318103	0.006342
Up	NDC80	85	8.84E-03	6063802	0.331876	0.02821
Up	MSH6	102	6.57E-03	5782582	0.344048	0.024073
Up	C16orf59	10	9.83E-04	623078	0.253207	0
Up	SPC25	35	2.22E-03	1258912	0.297914	0.067227
Up	PGR	78	7.39E-03	3889220	0.341319	0.018596
Up	HELLS	49	4.62E-03	4012396	0.32025	0.002551
Up	ALDH1A3	9	1.00E-03	327000	0.316094	0.027778
Up	CITED1	13	6.64E-04	885046	0.297488	0
Up	SATB1	56	4.36E-03	7006108	0.303033	0
Up	MCM3	157	1.32E-02	10716498	0.373064	0.040993
Up	DSN1	53	5.16E-03	2728942	0.331587	0.051765

Up	GRIK3	5	4.65E-04	177868	0.284129	0
Up	FANCI	62	4.57E-03	6129518	0.317212	0
Up	KIF23	64	6.11E-03	4207438	0.33089	0.011634
Up	BRCA1	561	1.06E-01	87536324	0.398937	0.005238
Up	UGT2B15	1	0.00E+00	0	1	0
Up	RRM2	43	2.55E-03	2289082	0.310124	0.002215
Up	MSRB3	18	3.02E-03	938432	0.296968	0
Up	THBS1	58	9.61E-03	3401560	0.305443	0.011039
Up	FGFR2	100	1.83E-02	9663342	0.314354	0.001052
Up	FANCA	88	8.54E-03	5237228	0.336198	0.011218
Up	TIAM2	10	6.90E-04	202162	0.283879	0
Up	LIG1	22	1.50E-03	713850	0.327365	0.060606
Up	SYT8	1	0.00E+00	0	0.241343	0
Up	CCDC85A	3	3.11E-04	304696	0.217336	0
Up	PGR	78	7.39E-03	3889220	0.341319	0.018596
Up	RUNX1T1	68	7.52E-03	3492070	0.324517	0.020979
Up	CST6	37	5.45E-03	2003914	0.296613	0.001502
Up	PSMC3IP	25	1.42E-03	698384	0.305746	0
Up	C21orf58	3	1.99E-05	27522	0.25846	0
Up	TMEM79	18	3.47E-03	2079642	0.245371	0
Up	MARS	91	6.82E-03	6147160	0.338953	7.08E-03
Up	KCNC1	10	4.99E-04	291266	0.239658	0
Up	CCNE1	84	4.39E-03	2638518	0.349318	0.045468
Up	TEAD1	28	2.56E-03	801936	0.291825	0.027692
Up	POLE2	35	3.46E-03	4228544	0.295608	0
Up	KIFC1	21	1.85E-03	874666	0.324224	0.02381
Up	E2F2	27	4.39E-04	357046	0.304104	0.042735
Up	DONSON	3	4.28E-06	5532	0.260231	0
Up	NCAPG	53	5.64E-03	5729282	0.310856	0.004354
Up	HAUS8	53	4.85E-03	4406746	0.29949	0
Up	MCM5	254	2.34E-02	19431894	0.382754	0.019825
Up	IGFBP4	8	4.52E-04	341784	0.249865	0
Up	GPR37L1	2	1.00E+00	2	1	0
Up	AJUBA	26	2.32E-03	842226	0.314262	0.027692
Up	TRIP13	127	2.15E-02	21279310	0.322233	7.74E-04
Up	PREP	38	4.88E-03	2241216	0.319663	0.007112
Up	PTPN14	67	6.38E-03	9281020	0.306778	0
Up	GPM6B	14	1.35E-03	1061888	0.275318	0
Up	SCUBE2	3	0.00E+00	0	1	0
Up	UNG	20	1.46E-03	717578	0.319299	0.047368
Up	MB21D2	17	1.61E-03	1076068	0.286501	0
Up	CADM1	17	1.13E-03	393962	0.291311	0.009524
Up	DTL	62	3.66E-03	2737290	0.325943	0.016922
Up	KNTC1	26	2.58E-03	2381948	0.288421	0.00E+00
Up	RFC3	48	1.96E-03	2104388	0.326157	0.078901
Up	FAM111B	8	3.40E-04	252564	0.275388	0
Up	PHACTR1	3	2.38E-06	1146	0.279176	0.333333
Up	TCF19	16	2.40E-04	251912	0.293911	0

Up	SAMD4A	18	1.11E-03	1195764	0.289898	0
Up	FBXO5	35	1.95E-03	1147486	0.325073	0.023529
Up	ARHGEF17	15	7.40E-04	323618	0.299879	0.115385
Up	GINS3	36	2.61E-03	1293176	0.317775	0.057143
Up	ABAT	37	4.76E-03	3967944	0.294005	0.001681
Up	CENPM	15	2.55E-03	2394524	0.251893	0
Up	SPAG5	86	1.23E-02	5947608	0.31324	0.002189
Up	RUNX1	86	6.15E-03	9166940	0.313119	0.00459
Up	STARD13	17	1.57E-03	2267444	0.272171	0
Up	NEDD9	54	5.69E-03	4774130	0.311246	0
Up	PMEPA1	8	9.60E-04	542642	0.266331	0
Up	POLD1	79	5.82E-03	4175544	0.341572	0.027588
Up	CDC7	36	2.19E-03	1008880	0.340563	0.131907
Up	MCM4	111	5.55E-03	4472664	0.365956	0.070846
Up	GINS4	43	4.10E-03	2281010	0.31526	0.065338
Up	ZWINT	98	1.20E-02	6063532	0.323785	0.01299
Up	MCM5	254	2.34E-02	19431894	0.382754	0.019825
Up	NETO2	31	5.64E-03	2172612	0.268268	0
Up	FLNB	327	5.41E-02	36113460	0.371475	0.004957
Up	DCN	43	8.70E-03	2686162	0.321398	0.014396
Up	ASF1B	52	3.70E-03	1914596	0.337729	0.079186
Up	RFC3	48	1.96E-03	2104388	0.326157	0.078901
Up	KDM4B	31	1.49E-03	869694	0.309026	0.010753
Up	HJURP	21	9.74E-04	486394	0.302069	0.046784
Up	SEC14L2	2	1.00E+00	2	1	0
Up	CUX2	1	0.00E+00	0	0.250349	0
Up	DNM1L	67	9.17E-03	7512686	0.315692	9.62E-04
Up	GRIK4	4	9.30E-04	855018	0.253555	0
Up	CDCA5	55	8.45E-03	3864816	0.319094	0.004354
Up	PKMYT1	20	1.43E-03	963304	0.296831	0.021053
Up	KCNC1	10	4.99E-04	291266	0.239658	0
Up	KIF15	27	2.75E-03	1546400	0.295296	0
Up	POLA1	54	4.76E-03	2614278	0.34945	0.069881
Up	C19orf57	32	2.63E-03	1572790	0.301758	0
Up	NUSAP1	35	3.63E-03	1599426	0.321783	0.008913
Up	SIPA1	17	9.60E-04	869620	0.292991	0.051471
Up	CHST14	6	9.67E-04	656244	0.256855	0
Up	E2F1	135	1.25E-02	9945144	0.348752	0.019302
Up	ARHGEF19	4	3.27E-04	309528	0.245259	0
Up	S100A8	78	6.95E-03	5104874	0.349753	0.02386
Up	WNT7B	7	8.24E-04	679132	0.255341	0
Up	ESPL1	39	3.37E-03	4066794	0.300676	0
Up	CKMT2	17	1.28E-03	948980	0.270008	0
Up	SAMD4A	18	1.11E-03	1195764	0.289898	0
Up	GRIK4	4	9.30E-04	855018	0.253555	0
Up	ARHGAP15	9	5.86E-04	435304	0.284781	0
Up	LHX2	5	1.24E-03	712548	0.222767	0
Up	MCM6	103	5.95E-03	4177406	0.363995	0.072871

Up	ODAM	5	6.23E-04	477384	0.278178	0
Up	CD24	4	6.21E-04	396070	0.270438	0
Up	RAD54L	9	4.59E-04	177116	0.27937	0
Up	DHFR	17	1.73E-03	1065814	0.309633	0
Up	KRT83	11	1.64E-04	197234	0.274732	0
Up	S100A9	115	1.02E-02	8326754	0.341121	0.01122
Up	GRHL3	8	6.20E-04	799722	0.255614	0
Up	PBK	112	7.93E-03	7887630	0.329622	0.002002
Up	BARD1	277	4.45E-02	22735780	0.379023	0.008042
Up	TBXAS1	1	0.00E+00	0	0.23205	0
Up	SMTN	12	3.42E-04	194140	0.30789	0
Up	HIST1H2AL	55	4.27E-03	2327038	0.337552	0.022222
Up	MCM7	283	3.75E-02	29997454	0.383095	0.016777
Up	EGFL6	15	2.56E-03	1824678	0.265378	0
Up	PDGFB	83	1.22E-02	9472946	0.296627	0.00216
Up	MCEE	3	6.20E-04	480818	0.259384	0
Up	SEC14L2	2	1.00E+00	2	1	0
Up	WTIP	11	4.18E-04	172790	0.282401	0.054545
Up	BLM	93	8.72E-03	6062032	0.338953	0.024176
Up	ESCO2	19	5.29E-04	306544	0.30452	0.023392
Up	UHRF1	70	4.95E-03	3499650	0.338597	0.028973
Up	RECQL4	26	2.33E-03	1959336	0.292049	0
Up	LY6E	3	6.20E-04	507162	0.234708	0
Up	SMOC2	1	0.00E+00	0	0.230459	0
Up	BANK1	5	3.16E-04	324842	0.274615	0
Up	TK1	182	3.28E-02	29837994	0.331757	1.86E-04
Up	CCNA2	125	1.01E-02	5837458	0.357127	0.031587
Up	MCM2	875	1.67E-01	127048732	0.414382	0.003502
Up	TRIM6	8	8.74E-05	61480	0.271782	0
Up	KIF11	134	1.65E-02	13556478	0.332064	0.001851
Up	CYP2J2	3	6.20E-04	368706	0.220303	0
Up	CECR6	1	0.00E+00	0	1	0
Up	ACOT7	38	5.44E-03	2127562	0.324877	0.009957
Up	AMTN	11	1.58E-03	776002	0.259875	0
Up	FERMT1	16	1.33E-03	1002112	0.286348	0
Up	DSCC1	20	1.73E-03	1324922	0.28555	0.110526
Up	NFIB	12	1.42E-03	848700	0.287637	0
Up	MMS22L	29	1.33E-03	634070	0.332441	0.282051
Up	DCLRE1B	15	9.94E-04	1422128	0.281061	0
Up	INHBA	17	2.84E-03	2587244	0.21417	0
Up	AURKB	177	2.21E-02	23052080	0.353954	0.007422
Up	CENPM	15	2.55E-03	2394524	0.251893	0
Up	CHAF1B	44	2.68E-03	1561278	0.345744	0.064482
Up	GINS1	9	3.41E-04	141104	0.288964	0.388889
Up	KIF20A	38	4.38E-03	1576352	0.325368	0.018492
Up	FLNA	338	5.59E-02	37326480	0.392197	0.010135
Up	PTGER3	113	2.27E-02	11141298	0.3123	1.58E-04
Up	SEMA3G	1	0.00E+00	0	1	0

Up	HIST1H1D	55	4.02E-03	2938548	0.334595	0.012121
Up	LCAT	6	1.55E-03	1121350	0.218026	0
Up	RHBDL2	2	1.00E+00	2	1	0
Up	NRGN	13	1.12E-03	744946	0.26809	0
Up	MKI67	63	5.20E-03	3343804	0.348545	0.023497
Up	KCNC3	2	2.53E-04	137500	0.271988	0
Up	TRAM2	8	6.25E-04	660838	0.26207	0
Up	ALDH7A1	41	4.06E-03	5078254	0.30537	0.00122
Up	DOK7	7	6.63E-04	598660	0.249469	0
Up	RFC5	71	3.21E-03	4409762	0.325762	0.034608
Up	RASSF7	33	3.18E-03	2892400	0.287586	0
Up	FLT4	34	3.71E-03	2686344	0.301518	0
Up	SPIN4	4	1.14E-05	18204	0.268603	0
Up	PLCE1	8	3.97E-04	404172	0.27337	0
Up	CTGF	27	2.69E-03	1513624	0.306822	0.011396
Up	CIT	22	2.60E-03	1310098	0.303503	0.008658
Up	GTSE1	141	1.88E-02	17091376	0.328967	0.002736
Up	PPM1J	16	1.60E-03	2322182	0.280182	0
Up	RASL11B	6	1.44E-04	125428	0.277436	0.00E+00
Up	EGLN3	241	4.36E-02	28153850	0.344544	0.001828
Up	MYBL1	8	2.12E-05	19452	0.259436	0
Up	XRCC3	151	2.03E-02	19264374	0.323023	9.84E-04
Up	ZFP36L2	11	9.77E-05	114652	0.280645	0
Up	RAD51AP1	16	1.02E-03	452868	0.306036	0.025
Up	CACNG4	17	2.95E-03	2056094	0.250631	0
Up	TRPM8	12	1.88E-03	1166534	0.253307	0
Up	MYL9	18	1.21E-03	366208	0.320139	0.026144
Up	LRP4	28	5.22E-03	2887648	0.284004	0
Up	XYLT1	3	3.13E-04	209990	0.255008	0
Up	EFEMP1	59	8.70E-03	7824456	0.299046	0
Up	PCP4L1	1	0.00E+00	0	0.222898	0
Up	ID3	46	5.45E-03	2395046	0.321735	0.019027
Up	PIP	22	2.20E-03	877002	0.317384	0.030303
Up	SULT2B1	13	1.58E-03	1097000	0.235857	0
Up	TONSL	35	8.84E-04	819624	0.345559	0.24053
Up	GREB1	13	3.89E-04	125492	0.288151	0.012821
Up	FOLR1	8	1.66E-04	117984	0.272148	0
Up	CDT1	70	3.75E-03	2093976	0.351544	0.062774
Up	IGFBP5	23	2.67E-03	1813404	0.287611	0.007905
Up	PTPN14	67	6.38E-03	9281020	0.306778	0
Up	SLC23A1	16	3.61E-04	357840	0.268057	0.00E+00
Up	CDKN2C	47	4.07E-03	2831574	0.311426	0.004625
Up	CISH	31	3.33E-03	2251712	0.300186	0
Up	NRAP	11	9.64E-04	347178	0.283967	0.054545
Up	TPM1	127	1.65E-02	9646758	0.353586	0.012903
Up	ANLN	148	1.16E-02	8481006	0.338313	0.022551
Up	PTGER3	113	2.27E-02	11141298	0.3123	1.58E-04
Up	JPH2	3	3.98E-06	4314	0.249913	0

Up	VGLL1	4	4.93E-06	1706	0.242122	0.5
Up	PREX1	8	8.74E-05	60518	0.260936	0
Up	ANKRD2	8	9.51E-04	769462	0.278526	0
Up	ETNK2	5	3.12E-04	262594	0.246958	0
Up	TEAD4	34	3.15E-03	1931170	0.304133	0.01426
Up	TRH	3	3.21E-04	245526	0.232845	0
Up	POLA2	60	6.88E-03	4370234	0.313804	0.005085
Up	CCDC85A	3	3.11E-04	304696	0.217336	0
Up	OLFML3	6	3.70E-04	256174	0.253108	0
Up	SERTAD4	14	7.84E-04	597382	0.284117	0
Up	LRRC4C	25	5.16E-03	1436010	0.253247	0
Up	TRIM58	8	3.30E-04	207472	0.259343	0
Up	FBP1	49	6.49E-03	7634696	0.298865	0
Up	PHF19	44	4.64E-03	1855502	0.316373	0.013937
Up	ITPKA	8	6.58E-04	260210	0.283269	0.035714
Up	BFSP2	18	1.75E-03	1291774	0.259708	6.54E-03
Up	SLC40A1	4	9.30E-04	721224	0.259405	0
Up	MND1	7	7.03E-04	323310	0.270121	0
Up	PTGER3	113	2.27E-02	11141298	0.3123	1.58E-04
Up	E2F8	8	4.00E-04	315810	0.275294	0
Up	CDCA3	28	3.03E-03	2047980	0.296069	0
Up	KIAA1161	15	2.91E-03	3094638	0.264054	0
Up	KRT8P12	2	3.10E-04	151488	0.22739	0
Up	PTPN14	67	6.38E-03	9281020	0.306778	0
Up	BOP1	61	6.72E-03	12594626	0.306632	0
Up	KRT8	82	7.70E-03	10821362	0.323185	0
Up	SPC24	41	2.81E-03	1262284	0.308937	0.069512
Up	RASSF2	31	2.03E-03	2598442	0.298175	0
Up	KIF5C	33	3.25E-03	3728976	0.301181	0
Up	CLIC3	10	1.28E-03	858242	0.275706	0
Up	CKAP2L	4	3.79E-06	9276	0.260905	0
Up	FRMD3	11	1.93E-03	1205382	0.257213	0
Up	C2orf71	5	6.22E-04	360648	0.243382	0
Up	TMEM139	3	1.04E-05	7256	0.235144	0
Up	SPINK4	7	1.55E-03	857744	0.210901	0
Up	DHFR	17	1.73E-03	1065814	0.309633	0
Up	PTGER3	113	2.27E-02	11141298	0.3123	1.58E-04
Up	PLCE1	8	3.97E-04	404172	0.27337	0
Up	ACTBL2	185	2.86E-02	16272402	0.362931	0.008167
Up	ACOX1	44	6.27E-03	1946752	0.317696	0.0074
Up	OLFM1	14	1.15E-03	1040808	0.278911	0
Up	WISP2	12	1.63E-03	1519160	0.261476	0
Up	MS4A7	3	6.20E-04	375602	0.226687	0
Up	REEP5	76	1.70E-02	12787628	0.293537	0
Up	SLITRK4	2	1.13E-04	38016	0.239854	0
Up	LYPD5	1	0.00E+00	0	0.214177	0
Up	IVL	39	4.58E-03	5049884	0.275694	0
Up	SSX4B	4	3.12E-04	228054	0.247223	0

Up	ERCC6L	29	1.89E-03	946908	0.314262	0.009852
Up	MYBL1	8	2.12E-05	19452	0.259436	0
Up	GRPR	53	1.29E-02	6708638	0.260452	0
Up	IGDCC3	1	0.00E+00	0	1	0
Up	SLC43A3	7	1.55E-03	858216	0.245633	0
Up	RBBP8	56	4.84E-03	2718260	0.33496	0.027254
Up	LMNB1	111	1.12E-02	9127646	0.348262	0.009865
Up	ID1	47	4.04E-03	1960290	0.312255	0.007401
Up	NCAM2	6	4.08E-04	337458	0.256773	0
Up	SERPINF1	13	1.68E-03	1767720	0.26976	0
Up	S100A16	38	4.98E-03	4650996	0.302238	0
Up	SERPINA6	9	1.70E-03	1379738	0.260968	0
Up	SKA3	47	5.47E-03	2017856	0.317025	0.010628
Up	COL27A1	3	0.00E+00	0	0.20884	0
Up	OLFM1	14	1.15E-03	1040808	0.278911	0
Up	BUB1B	98	9.89E-03	7358968	0.331433	0.016009
Up	FBXL18	44	4.85E-03	5150848	0.312754	0
Up	RANBP3L	3	3.11E-04	195116	0.23346	0
Up	ABI3BP	9	7.44E-04	541158	0.282327	0
Up	CDH10	7	7.64E-04	937234	0.268235	0
Up	PYROXD2	3	3.16E-04	145116	0.242313	0
Up	ELOVL2	10	1.86E-03	1157104	0.273451	0
Up	CCL2	24	3.44E-03	1764994	0.320155	0.082251
Up	CRYM	4	3.11E-04	285950	0.255018	0
Up	ABCC13	1	0.00E+00	0	0.2056	0
Up	RABIF	31	4.23E-03	1557158	0.289119	0
Down	RRAGD	47	8.08E-03	4837168	0.268828	0.011101
Down	NEU1	13	1.22E-03	640030	0.256574	0
Down	BRI3	2	3.69E-04	186280	0.210717	0
Down	SLC27A1	1	0.00E+00	0	1	0
Down	DDIT4	17	1.21E-03	1143368	0.272206	0
Down	HSPA6	104	1.65E-02	6510190	0.336963	0.01531
Down	CXCL1	12	1.29E-03	1197244	0.242477	0
Down	INSIG1	22	2.84E-03	1814442	0.270601	0.035088
Down	SEL1L3	8	1.23E-03	522386	0.244845	0.071429
Down	TRIB3	41	7.43E-03	2466626	0.311404	0.006098
Down	GNPDA1	42	6.31E-03	2413398	0.289124	0.008974
Down	SAT2	9	4.50E-04	129452	0.252754	0.047619
Down	IDI1	6	4.63E-04	307216	0.24916	0
Down	MVD	35	5.21E-03	1145432	0.287573	0.039773
Down	LHX8	8	8.14E-04	500312	0.227933	0
Down	FTL	57	1.36E-02	4077762	0.299337	0.003367
Down	UBE2L6	52	9.78E-03	2338020	0.293019	0.004082
Down	TESK2	10	1.19E-03	1113212	0.254848	0
Down	DUSP13	81	1.72E-02	7321168	0.295305	0
Down	HIP1R	71	2.03E-02	5595266	0.308776	0.005968
Down	AMDHD2	4	3.92E-04	77922	0.236897	0.166667
Down	TSKU	2	1.00E+00	2	1	0

Down	BRI3	2	3.69E-04	186280	0.210717	0
Down	FTL	57	1.36E-02	4077762	0.299337	0.003367
Down	HSD17B7	14	1.85E-03	630098	0.244073	0
Down	CEACAM1	23	3.10E-03	1826872	0.270682	0
Down	VEGFB	16	2.32E-03	669816	0.249516	0.032967
Down	PLEKHA4	7	3.99E-04	253766	0.250834	0
Down	GPNMB	5	3.06E-04	173244	0.225672	0
Down	ACSS2	5	4.46E-04	85202	0.257074	0
Down	HPS1	9	2.23E-03	832514	0.22159	0
Down	IFIT3	73	8.51E-03	5187884	0.304881	0.024547
Down	ACSL1	10	2.40E-03	843632	0.270804	0.022222
Down	C15orf48	120	3.10E-02	15084098	0.277664	2.80E-04
Down	FDPS	54	1.00E-02	4547904	0.298019	0.006289
Down	FLCN	44	7.58E-03	2636654	0.30459	0.009514
Down	PLA2G3	2	1.25E-05	5812	0.189621	0
Down	LAMP3	68	1.75E-02	3274316	0.283627	0.001317
Down	KLHDC7B	1	0.00E+00	0	0.194197	0
Down	RHOQ	21	4.75E-03	1751864	0.272878	0
Down	NOS3	55	1.24E-02	6001878	0.303039	7.26E-04
Down	PPARGC1A	68	1.29E-02	7608658	0.298462	0.002195
Down	DDIT3	81	1.95E-02	5292882	0.307199	0.021422
Down	PRSS8	1	0.00E+00	0	0.179305	0
Down	IDH1	60	1.20E-02	5452874	0.298429	0.005445
Down	HOXB9	49	9.36E-03	3508558	0.28515	0
Down	KLHDC7B	1	0.00E+00	0	0.194197	0
Down	BEST1	4	6.17E-06	1950	0.197217	0
Down	MAGIX	23	4.95E-03	2997504	0.237959	0
Down	LPIN1	11	1.26E-03	1129650	0.264598	0
Down	FGF13	8	5.83E-04	108266	0.266683	0.133333
Down	MAF	27	1.88E-03	1106912	0.264883	0.016667
Down	IFI30	40	9.99E-03	2975508	0.250335	0
Down	MSMO1	14	1.57E-03	672992	0.264883	0
Down	SYPL2	12	2.89E-03	482460	0.292182	0.015152
Down	CLCN5	12	1.02E-03	625624	0.278077	0
Down	RORC	44	7.64E-03	3560208	0.282075	0.013742
Down	CASP5	8	8.17E-04	125332	0.237709	0
Down	CLCN7	27	4.43E-03	1257986	0.268601	0.008547
Down	ACP5	31	5.79E-03	1375136	0.299056	0.017241
Down	UAP1L1	6	7.92E-04	466412	0.21415	0
Down	RCBTB1	20	2.32E-03	933752	0.297642	0.026316
Down	OAS1	6	8.09E-04	390706	0.221527	0
Down	ICAM1	174	3.59E-02	38584000	0.302869	0
Down	SDCBP	238	6.95E-02	30023038	0.325812	1.44E-04
Down	GAD1	18	1.39E-03	910366	0.26951	0
Down	LCN2	21	1.74E-03	1511404	0.247305	0
Down	GGCT	12	1.81E-03	1114252	0.272343	0
Down	HMGCR	38	5.58E-03	4782990	0.262202	0.010084
Down	GDF15	10	1.26E-03	525132	0.226654	0

Down	HSPA6	104	1.65E-02	6510190	0.336963	0.01531
Down	COL16A1	5	3.74E-04	290696	0.240806	0
Down	MAFG	38	4.38E-03	1912960	0.291442	6.67E-02
Down	STEAP1	1	0.00E+00	0	1	0
Down	HMGCS1	33	6.50E-03	1510674	0.27598	0
Down	GM2A	27	4.46E-03	3331802	0.277365	0
Down	SEPHS2	13	2.08E-03	1554682	0.242749	0
Down	PRR15	2	1.20E-05	2996	0.238725	0
Down	GPNMB	5	3.06E-04	173244	0.225672	0
Down	LGALS9C	27	5.08E-03	2400172	0.241817	0.011396
Down	SERPINA3	41	8.61E-03	11787492	0.261999	0
Down	RILP	15	2.39E-03	849296	0.238609	0
Down	GULP1	14	2.17E-03	1403364	0.251416	0
Down	IL3RA	19	3.30E-03	2031720	0.256016	0
Down	PLD6	12	2.40E-03	1115656	0.256599	0
Down	AHNAK2	22	4.42E-03	2194462	0.270277	0
Down	HSD17B7	14	1.85E-03	630098	0.244073	0
Down	HSD17B7	14	1.85E-03	630098	0.244073	0
Down	NUCB2	31	3.77E-03	1759036	0.289108	0
Down	EBP	40	8.30E-03	5770036	0.292402	0
Down	LSS	15	2.82E-03	2083666	0.268815	0
Down	CLCN5	12	1.02E-03	625624	0.278077	0
Down	SERPINA3	41	8.61E-03	11787492	0.261999	0
Down	F7	24	3.72E-03	3071938	0.266893	0
Down	FRY	4	7.40E-04	344288	0.223908	0
Down	CFB	15	1.85E-03	1803376	0.245545	0
Down	CPVL	52	1.08E-02	8155370	0.282208	0
Down	LSS	15	2.82E-03	2083666	0.268815	0
Down	WDR81	16	1.67E-03	1675254	0.267764	0
Down	NUCB2	31	3.77E-03	1759036	0.289108	0
Down	RAB17	17	4.36E-03	1612168	0.234822	0
Down	TMEM86A	1	0.00E+00	0	0.234182	0
Down	CXCL1	12	1.29E-03	1197244	0.242477	0
Down	SQSTM1	308	9.98E-02	39034828	0.369146	0.003364
Down	ST6GAL1	19	3.33E-03	1500128	0.232314	0
Down	CLGN	38	6.19E-03	6133040	0.278951	0
Down	GGCT	12	1.81E-03	1114252	0.272343	0
Down	CASP4	60	1.28E-02	8454490	0.288985	0
Down	RAB26	6	1.85E-03	758630	0.23144	0
Down	STEAP1	1	0.00E+00	0	1	0
Down	PSMG2	46	9.80E-03	3370878	0.276445	0.002899
Down	KLHL24	18	3.08E-03	2208176	0.274539	0
Down	UNC5B	13	1.99E-03	1202896	0.253559	0
Down	CYP27A1	6	4.83E-04	246112	0.241289	0
Down	ARHGAP12	10	2.16E-03	980814	0.250023	0
Down	RHCG	1	0.00E+00	0	0.200067	0
Down	SERPINA3	41	8.61E-03	11787492	0.261999	0
Down	SLC38A7	11	1.29E-03	662016	0.235404	0

Down	TSPYL2	29	6.09E-03	4125992	0.260324	0
Down	HS1BP3	12	2.44E-03	950484	0.251171	0
Down	OASL	8	8.40E-04	278926	0.216548	0
Down	RAB17	17	4.36E-03	1612168	0.234822	0
Down	RAB3IL1	30	6.33E-03	3836770	0.261746	0
Down	ST3GAL1	15	4.09E-03	1482704	0.241299	0
Down	IFITM1	6	1.11E-03	578730	0.207384	0
Down	KMO	2	3.69E-04	106794	0.209998	0
Down	CCR10	17	4.05E-03	3623876	0.248566	0
Down	GULP1	14	2.17E-03	1403364	0.251416	0
Down	SHC2	13	1.13E-03	674860	0.272316	0
Down	IRS2	60	1.21E-02	7744912	0.305002	0
Down	VAT1	23	5.20E-03	1103354	0.291301	0
Down	RENBP	3	3.69E-04	109584	0.211838	0
Down	CRB3	7	9.37E-04	411966	0.229158	0
Down	MAPK8IP2	97	2.52E-02	9968290	0.312608	4.48E-04
Down	ISG15	199	5.19E-02	26453166	0.336858	0.002589
Down	NEURL2	1	0.00E+00	0	0.231995	0
Down	IL32	18	2.17E-03	1708286	0.253227	0
Down	CCDC28B	6	5.55E-04	306650	0.251043	0
Down	IRF9	35	6.79E-03	2243194	0.282193	0.007576
Down	CYP51A1	25	4.83E-03	2706892	0.260174	0
Down	TNFSF10	23	1.95E-03	739832	0.286994	0.07619
Down	ALDOC	72	1.68E-02	7843374	0.305053	0.001956
Down	IFIT2	44	3.50E-03	2082594	0.289495	0.049683
Down	LDLR	41	7.86E-03	1544814	0.300083	0.009447
Down	MT1H	3	1.50E-05	8418	0.22712	0
Down	ADM2	1	0.00E+00	0	1	0
Down	WDSUB1	1	0.00E+00	0	0.216808	0
Down	FLCN	44	7.58E-03	2636654	0.30459	0.009514
Down	BHLHE41	40	5.66E-03	1549870	0.29052	0.021337
Down	SLC36A1	8	2.70E-04	229466	0.252142	0
Down	RNF152	5	4.90E-04	383034	0.212996	0
Down	GAB2	50	8.36E-03	6513310	0.288032	0
Down	FABP6	1	0.00E+00	0	1	0
Down	IL32	18	2.17E-03	1708286	0.253227	0
Down	SLC7A11	7	4.38E-04	213000	0.22732	0
Down	SPIRE1	8	4.15E-04	483202	0.242738	0
Down	KLHL21	18	2.36E-03	2137996	0.261153	0
Down	ISG20	10	2.52E-03	1119988	0.229878	0
Down	IGF2R	44	9.05E-03	4060122	0.293226	0.002323
Down	RRAGC	31	6.71E-03	1700326	0.284581	0.051724
Down	IL15	7	1.18E-03	579144	0.235804	0
Down	ATP2C2	1	0.00E+00	0	1	0
Down	CCL20	5	8.61E-04	329886	0.241224	0
Down	MAFG	38	4.38E-03	1912960	0.291442	6.67E-02
Down	DHCR7	19	2.79E-03	2857100	0.260499	0
Down	OAS2	5	5.13E-04	225948	0.241429	0

Down	QPCT	21	1.37E-03	1251586	0.269551	0
Down	CYBA	17	2.93E-03	2005092	0.253191	0
Down	ELOVL6	1	0.00E+00	0	1	0
Down	BBC3	9	3.94E-04	251256	0.255642	0
Down	CXCL3	2	2.00E-08	2	0.170669	0
Down	CMTM8	1	0.00E+00	0	0.237105	0
Down	MT1X	5	2.15E-04	102730	0.253571	0
Down	SH3BGR	5	1.11E-03	388480	0.220831	0
Down	FUT3	15	2.86E-03	1637096	0.231856	0
Down	SLCO4A1	2	1.63E-05	7664	0.225156	0
Down	HMOX1	36	7.62E-03	4133476	0.274692	0
Down	MX1	47	9.89E-03	2605116	0.302109	2.02E-03
Down	EMP3	3	1.00E+00	6	1	0
Down	MAP1LC3B	375	1.01E-01	51072854	0.361868	0.002393
Down	MAP1LC3B	375	1.01E-01	51072854	0.361868	0.002393
Down	ALDH3B2	6	5.94E-04	120814	0.239305	0
Down	CXCL2	11	1.10E-03	858948	0.244062	0
Down	IFIT1	47	5.12E-03	2122902	0.297217	0.049029
Down	IFITM1	6	1.11E-03	578730	0.207384	0
Down	TPP1	29	4.78E-03	3709944	0.285963	0
Down	S100P	28	4.41E-03	3587638	0.262813	0
Down	NEURL3	1	0.00E+00	0	1	0
Down	BEX2	35	6.31E-03	2802748	0.278506	0.010084
Down	CXCL2	11	1.10E-03	858948	0.244062	0
Down	BIRC3	88	1.56E-02	9078926	0.305915	0.005746
Down	CRYAB	110	2.83E-02	15944744	0.304333	1.73E-04
Down	HERPUD1	33	5.56E-03	3109980	0.284955	0
Down	IFI44	2	3.09E-06	2708	0.229878	0
Down	AKR1C1	10	1.27E-03	482734	0.23842	0.071429
Down	SPIRE1	8	4.15E-04	483202	0.242738	0
Down	DDX60	4	4.15E-04	280796	0.241332	0
Down	DBP	13	2.55E-04	88482	0.263363	0.179487
Down	AHNAK2	22	4.42E-03	2194462	0.270277	0
Down	CCL5	46	1.02E-02	6920254	0.264844	0
Down	TLR2	48	8.52E-03	2959130	0.289541	9.66E-04
Down	LY96	5	5.80E-04	106326	0.246933	0.1
Down	KCNE4	8	1.86E-03	784230	0.228192	0
Down	DRAM1	3	4.15E-04	195424	0.237469	0
Down	SLC25A36	2	3.69E-04	180260	0.207352	0
Down	RHOQ	21	4.75E-03	1751864	0.272878	0
Down	MT1E	1	0.00E+00	0	0.225334	0
Down	SESN2	14	1.80E-03	355848	0.293846	0.021978
Down	CD70	79	2.25E-02	4500850	0.285556	0
Down	LMO2	156	3.81E-02	17153632	0.303276	5.16E-04
Down	STX3	68	1.73E-02	6420236	0.270169	4.66E-04
Down	AKR1C3	11	1.58E-03	550554	0.238672	0.036364
Down	TNFSF9	32	7.45E-03	4574170	0.250764	0
Down	HSPBAP1	7	9.72E-04	597998	0.21796	0

Down	MT1X	5	2.15E-04	102730	0.253571	0
Down	NEK8	41	7.12E-03	2918182	0.292118	0.006748
Down	DNAJB9	45	1.14E-02	2109896	0.312122	0.006061
Down	FAM129A	8	8.20E-04	547228	0.244757	0
Down	SDR16C5	5	7.50E-04	369950	0.216254	0
Down	NFE2L1	36	3.82E-03	1897100	0.27755	0.02139
Down	NPHS1	19	2.36E-03	1968922	0.258584	0
Down	APOL2	31	5.72E-03	5003754	0.255617	0
Down	NAMPT	37	5.84E-03	1869520	0.297478	0.016807
Down	SLC25A36	2	3.69E-04	180260	0.207352	0
Down	BTN3A1	4	0.001108	322344	0.17555	0
Down	MT1F	2	3.69E-04	267452	0.16583	0
Down	SLC33A1	63	1.31E-02	10494070	0.289294	0.001536
Down	HSD17B14	21	3.30E-03	784300	0.256988	0
Down	GADD45G	59	8.25E-03	4913340	0.293703	0.011105
Down	SPRR1A	5	1.08E-04	157456	0.272357	0
Down	IL6R	23	5.01E-03	4621722	0.2469	0
Down	INHBE	17	2.89E-03	646640	0.30588	0.038095
Down	CSAG1	3	1.70E-04	85516	0.233034	0
Down	SCGB1D1	72	2.18E-02	6267242	0.289108	0.001174
Down	PRNP	93	2.58E-02	4993604	0.32546	0.003907
Down	CIITA	31	3.84E-03	5315770	0.268921	0
Down	GBP2	45	7.57E-03	2116944	0.302565	0.015152
Down	RNF43	23	2.90E-03	2367220	0.262113	0
Down	TUBB2B	113	2.79E-02	16753828	0.318548	4.74E-04
Down	HYOU1	83	1.41E-02	6321556	0.32858	0.01355
Down	HSPA5	572	2.28E-01	144471588	0.382038	0.00229
Down	TMEM97	13	3.10E-03	1221104	0.277806	0
Down	CHN2	16	1.16E-03	955442	0.268242	0
Down	RETN	9	1.19E-03	347216	0.290069	0.095238
Down	IFIH1	18	2.52E-03	870152	0.267275	0
Down	GADD45A	87	1.62E-02	8909078	0.306019	0.005882
Down	APOL6	7	1.93E-04	111824	0.243065	0
Down	NR1D1	15	1.53E-03	1068448	0.260312	0
Down	JMY	10	1.08E-03	759210	0.257685	0
Down	WARS	68	1.88E-02	4557734	0.314442	0.010256
Down	VEGFA	41	1.09E-02	3516642	0.291066	0.005398
Down	VEGFA	41	1.09E-02	3516642	0.291066	0.005398
Down	GADD45G	59	8.25E-03	4913340	0.293703	0.011105
Down	SAT1	82	1.73E-02	7447064	0.302143	0.002215
Down	PEG10	42	7.22E-03	4300340	0.276685	0
Down	UPP1	14	2.87E-03	1836234	0.25344	0
Down	UVRAG	64	1.32E-02	5391342	0.287161	0
Down	S100A14	21	2.32E-03	1865864	0.27629	0
Down	CTAGE1	1	0.00E+00	0	1	0
Down	CREBRF	6	7.47E-04	544330	0.216202	0
Down	SQLE	15	3.14E-03	488538	0.257685	0
Down	FAM83G	10	2.50E-04	237310	0.249528	0

Down	FICD	2	3.69E-04	98746	0.231737	0
Down	FEZ1	131	3.49E-02	9788284	0.309978	6.15E-04
Down	ICAM4	12	1.53E-03	1072152	0.247068	0
Down	SEC24D	35	7.39E-03	5371112	0.271619	0
Down	CBR3	19	3.02E-03	660950	0.285466	0.005848
Down	GULP1	14	2.17E-03	1403364	0.251416	0
Down	KMO	2	3.69E-04	106794	0.209998	0
Down	PDE2A	6	4.06E-04	294964	0.234802	0
Down	AKR1C1	10	1.27E-03	482734	0.23842	0.071429
Down	RSAD2	4	3.96E-04	118476	0.207297	0
Down	HRK	8	3.60E-04	175996	0.25388	0
Down	SAT1	82	1.73E-02	7447064	0.302143	0.002215
Down	CERS1	3	4.15E-05	3040	0.270061	0
Down	ZNF165	23	3.37E-03	2099520	0.261835	0
Down	KRT75	23	1.80E-03	763652	0.285586	0.01581
Down	MT1A	5	5.65E-04	347096	0.242586	0
Down	FOXD1	2	7.97E-06	1764	0.195367	0
Down	RHOQ	21	4.75E-03	1751864	0.272878	0
Down	HK2	62	1.78E-02	6653544	0.304213	0.003702
Down	IRF1	37	4.00E-03	2053822	0.300466	0.010511
Down	UPP1	14	2.87E-03	1836234	0.25344	0
Down	SRXN1	39	6.48E-03	4447774	0.270304	0
Down	PLA2G4C	4	1.09E-04	79442	0.231628	0
Down	MT2A	25	4.85E-03	3334524	0.24803	0
Down	NAMPT	37	5.84E-03	1869520	0.297478	0.016807
Down	SOD2	82	1.81E-02	7936274	0.298347	0.001582
Down	SLCO4C1	4	4.15E-05	30006	0.238978	0
Down	MAPK6	392	1.10E-01	73838870	0.345146	2.66E-04
Down	LTB	6	7.42E-04	384226	0.228684	0
Down	UCN	4	1.11E-03	186408	0.212495	0
Down	TNFRSF10B	65	1.75E-02	5147152	0.30495	0.009135
Down	FADS3	17	4.98E-03	1297490	0.224698	0
Down	C10orf10	8	1.15E-03	791792	0.221581	0
Down	SAMD9L	4	3.55E-05	20960	0.234588	0
Down	BDKRB1	3	3.95E-04	94990	0.191675	0
Down	CDKN1C	24	3.13E-03	3423572	0.268162	0
Down	GTPBP2	22	5.62E-03	2448598	0.256089	0
Down	CEBPB	90	1.86E-02	9328346	0.317019	0.009666
Down	RELB	102	2.02E-02	7398234	0.32946	0.005772
Down	GTF2IRD1	45	9.78E-03	5083078	0.279498	0
Down	RAB33A	8	1.21E-03	573140	0.22851	0
Down	S100A3	17	3.69E-03	2231346	0.240485	0
Down	LARP6	3	5.83E-04	249158	0.23145	0
Down	SLC43A1	3	3.75E-04	49526	0.195142	0
Down	BCL2L11	69	1.31E-02	5246600	0.306643	0.001357
Down	KCP	1	0.00E+00	0	0.234213	0
Down	PLAU	33	6.93E-03	1729004	0.28476	0.004301
Down	KLF4	30	3.05E-03	2819388	0.290504	0

Down	CYP1A2	8	7.86E-04	466614	0.202113	0
Down	ITPRIP	23	3.57E-03	1262274	0.268096	0
Down	IFIT2	44	3.50E-03	2082594	0.289495	0.049683
Down	TNFAIP3	93	2.03E-02	10677758	0.314423	0.00757
Down	EIF2AK3	47	7.96E-03	1920892	0.307164	0.011111
Down	SPAG9	71	1.78E-02	3590982	0.314095	0.001705
Down	RORA	32	4.09E-03	1225356	0.289758	0.009195
Down	IL23A	9	1.59E-03	214866	0.237271	0
Down	NDRG1	115	2.65E-02	10792318	0.338184	0.006712
Down	ULBP1	10	2.96E-03	1748050	0.180898	0
Down	TRIM16L	10	1.52E-03	978418	0.242119	0
Down	IFRD1	19	3.44E-03	1068842	0.274817	0
Down	JMY	10	1.08E-03	759210	0.257685	0
Down	RAB24	14	3.35E-03	1794294	0.211317	0
Down	ALDH1L2	14	2.41E-03	1598046	0.248224	0
Down	PABPC1L	6	3.96E-04	393670	0.255932	0
Down	RAB39B	24	6.86E-03	1741344	0.268948	0.003623
Down	DNAJC10	65	1.00E-02	3880912	0.325479	0.020192
Down	SERPINB8	27	4.39E-03	2460148	0.268654	0
Down	TMEM140	2	1.03E-05	4028	0.209243	0
Down	DAPP1	25	4.18E-03	3003076	0.25694	0
Down	GTPBP2	22	5.62E-03	2448598	0.256089	0
Down	VLDLR	12	6.37E-04	256764	0.236908	0
Down	SLC6A12	4	7.49E-04	362144	0.231717	0
Down	SLC5A3	15	2.04E-03	1442568	0.241278	0
Down	IL21R	4	1.11E-03	318462	0.197902	0
Down	MAFF	38	3.78E-03	1256886	0.28012	0.060317
Down	ZNF365	3	3.95E-04	163252	0.233013	0
Down	PARP14	13	1.21E-03	1139134	0.249862	0
Down	BDKRB2	21	5.52E-03	1684446	0.270317	0.004762
Down	ARID5A	21	2.85E-03	2157550	0.26075	0
Down	ATF3	70	1.13E-02	6148500	0.307129	0.027217
Down	SBSN	17	1.96E-03	1880156	0.25798	0
Down	NAMPT	37	5.84E-03	1869520	0.297478	0.016807
Down	PPP1R15A	32	5.83E-03	6327942	0.26612	0
Down	MT1M	2	2.20E-06	150	0.252212	0
Down	MIA2	1	0.00E+00	0	1	0

Table S7. miRNA - target gene interaction table

Regulation	Genes	Degree	MicroRNA	Regulation	Genes	Degree	MicroRNA
Up	OLR1	80	hsa-mir-1914-3p	Down	RRAGD	59	hsa-mir-548g-5p
Up	CHST8	1	hsa-mir-335-5p	Down	NEU1	1	hsa-mir-155-5p
Up	KLF8	54	hsa-mir-1236-3p	Down	BRI3	2	hsa-mir-484
Up	CFH	1	hsa-mir-146a-5p	Down	SLC27A1	95	hsa-mir-6797-5p
Up	TCF4	15	hsa-mir-519d-3p	Down	DDIT4	82	hsa-mir-8079
Up	HIST1H1B	19	hsa-mir-1-1	Down	HSPA6	57	hsa-mir-6873-3p
Up	ASPM	6	hsa-let-7a-5p	Down	CXCL1	4	hsa-mir-1-1

Up	H19	1	hsa-mir-375	Down	INSIG1	85	hsa-mir-8073
Up	KCNB1	67	hsa-mir-20a-5p	Down	SEL1L3	22	hsa-mir-877-3p
Up	COL12A1	29	hsa-mir-520d-5p	Down	TRIB3	39	hsa-mir-6808-5p
Up	DIAPH3	1	hsa-mir-155-5p	Down	GNPDA1	5	hsa-mir-3157-5p
Up	ZNF185	31	hsa-mir-1271-5p	Down	SAT2	1	hsa-mir-186-5p
Up	HSPB11	1	hsa-mir-155-5p	Down	IDI1	5	hsa-mir-193b-3p
Up	HIST1H3F	14	hsa-mir-9-5p	Down	MVD	2	hsa-mir-124-3p
Up	MYZAP	22	hsa-mir-527	Down	FTL	2	hsa-mir-133a-3p
Up	CDC45	2	hsa-mir-455-3p	Down	TESK2	17	hsa-mir-6829-3p
Up	ACTA1	25	hsa-let-7c-5p	Down	HIP1R	4	hsa-mir-92a-3p
Up	SFXN2	3	hsa-mir-132-3p	Down	AMDHD2	19	hsa-mir-6856-3p
Up	KLF17	17	hsa-mir-24-3p	Down	TSKU	78	hsa-mir-6810-3p
Up	GLDC	1	hsa-mir-423-3p	Down	BRI3	2	hsa-let-7e-5p
Up	THBS1	95	hsa-mir-198	Down	FTL	2	hsa-mir-16-5p
Up	ASPM	6	hsa-mir-215-5p	Down	HSD17B7	2	hsa-mir-155-5p
Up	CDH18	1	hsa-let-7e-5p	Down	VEGFB	18	hsa-mir-6768-5p
Up	DIAPH3	1	hsa-mir-155-5p	Down	PLEKHA4	2	hsa-mir-124-3p
Up	PLXNA1	27	hsa-mir-30a-5p	Down	GPNMB	1	hsa-mir-26b-5p
Up	CALD1	5	hsa-mir-34a-5p	Down	ACSS2	1	hsa-mir-335-5p
Up	KRT4	2	hsa-mir-26b-5p	Down	HPS1	2	hsa-mir-93-5p
Up	HELLS	11	hsa-mir-138-2-3p	Down	IFIT3	23	hsa-mir-4438
Up	PCNA	27	hsa-mir-4799-3p	Down	ACSL1	10	hsa-mir-154-5p
Up	DISP1	2	hsa-mir-124-3p	Down	C15orf48	11	hsa-mir-4436b-5p
Up	CCNE2	41	hsa-mir-449a	Down	FDPS	2	hsa-mir-30c-2-3p
Up	HIST1H3B	53	hsa-mir-146a-3p	Down	FLCN	27	hsa-mir-6838-5p
Up	SULF2	11	hsa-mir-193b-3p	Down	LAMP3	8	hsa-mir-5692c
Up	EXO1	3	hsa-mir-30e-5p	Down	KLHDC7B	1	hsa-mir-335-5p
Up	FSTL1	25	hsa-mir-1208	Down	RHOQ	25	hsa-mir-3941
Up	TMEM79	14	hsa-mir-302c-5p	Down	NOS3	3	hsa-mir-155-5p
Up	SYNPO	16	hsa-mir-10b-5p	Down	PPARGC1A	23	hsa-mir-1277-5p
Up	HIST2H3A	40	hsa-mir-299-3p	Down	DDIT3	8	hsa-mir-4645-3p
Up	ZWINT	98	hsa-mir-548b-5p	Down	PRSS8	1	hsa-mir-335-5p
Up	PCNA	27	hsa-mir-7161-5p	Down	IDH1	4	hsa-mir-30c-5p
Up	HIST1H2BF	28	hsa-mir-6513-5p	Down	HOXB9	2	hsa-mir-215-5p
Up	CDK2	59	hsa-mir-376a-3p	Down	KLHDC7B	1	hsa-mir-335-5p
Up	SLC12A4	5	hsa-mir-92a-3p	Down	MAGIX	1	hsa-mir-26b-5p
Up	UHRF1	14	hsa-mir-10a-5p	Down	LPIN1	8	hsa-mir-21-5p
Up	ATAD2	36	hsa-mir-548n	Down	MAF	19	hsa-mir-5100
Up	KIF23	60	hsa-mir-331-3p	Down	IFI30	3	hsa-mir-149-5p
Up	HAUS8	35	hsa-mir-410-3p	Down	MSMO1	63	hsa-mir-6864-3p
Up	LHFP	13	hsa-mir-6818-5p	Down	SYPL2	1	hsa-mir-615-3p
Up	TYMS	11	hsa-mir-615-3p	Down	CLCN5	21	hsa-mir-6783-5p
Up	MCM10	21	hsa-mir-3664-5p	Down	RORC	4	hsa-mir-148b-3p
Up	ANO2	1	hsa-mir-26b-5p	Down	CASP5	8	hsa-mir-4480
Up	CYR61	33	hsa-mir-630	Down	CLCN7	31	hsa-mir-6791-5p
Up	HIST1H2AI	28	hsa-mir-3122	Down	RCBTB1	21	hsa-mir-4768-3p
Up	HIST1H4D	6	hsa-mir-328-3p	Down	OAS1	1	hsa-mir-335-5p
Up	CDC25A	50	hsa-mir-497-5p	Down	ICAM1	35	hsa-mir-2467-5p
Up	WDHD1	2	hsa-mir-218-5p	Down	SDCBP	19	hsa-mir-5197-3p
Up	MKI67	85	hsa-mir-559	Down	GGCT	4	hsa-mir-30a-5p
Up	KRT8	42	hsa-mir-3689b-3p	Down	HMGCR	16	hsa-mir-362-3p
Up	ESCO2	41	hsa-mir-3646	Down	GDF15	1	hsa-mir-335-5p
Up	RAD51	113	hsa-mir-107	Down	HSPA6	57	hsa-mir-548as-3p

Up	RFC2	67	hsa-mir-202-3p	Down	COL16A1	1	hsa-mir-181a-5p
Up	RMI2	6	hsa-mir-4480	Down	MAFG	15	hsa-mir-5006-3p
Up	MYBL2	10	hsa-mir-505-3p	Down	HMGCS1	43	hsa-mir-4670-3p
Up	GINS2	31	hsa-mir-4279	Down	GM2A	37	hsa-mir-6134
Up	TGFB2	26	hsa-mir-378i	Down	SEPHS2	6	hsa-mir-19b-3p
Up	COL12A1	29	hsa-mir-4533	Down	PRR15	10	hsa-mir-4778-3p
Up	VTCN1	1	hsa-mir-335-5p	Down	GPNMB	1	hsa-mir-26b-5p
Up	GPR20	23	hsa-mir-1207-5p	Down	LGALS9C	1	hsa-mir-335-5p
Up	FRMD3	13	hsa-mir-1279	Down	SERPINA3	22	hsa-mir-6817-3p
Up	AMOTL2	78	hsa-mir-3125	Down	GULP1	20	hsa-mir-32-5p
Up	CCNE2	41	hsa-mir-503-5p	Down	PLD6	36	hsa-mir-6087
Up	MYCBP	42	hsa-mir-4430	Down	AHNAK2	17	hsa-mir-6165
Up	NDC80	1	hsa-mir-193b-3p	Down	HSD17B7	2	hsa-mir-335-5p
Up	MSH6	62	hsa-mir-324-3p	Down	HSD17B7	2	hsa-mir-155-5p
Up	C16orf59	2	hsa-mir-615-3p	Down	NUCB2	30	hsa-mir-4649-3p
Up	SPC25	63	hsa-mir-374a-5p	Down	EBP	4	hsa-mir-324-3p
Up	PGR	2	hsa-mir-126-3p	Down	LSS	5	hsa-mir-10b-5p
Up	HELLS	11	hsa-mir-877-3p	Down	CLCN5	21	hsa-mir-302f
Up	ALDH1A3	31	hsa-mir-3928-3p	Down	SERPINA3	22	hsa-mir-6500-3p
Up	SATB1	41	hsa-mir-581	Down	FRY	37	hsa-mir-4509
Up	MCM3	17	hsa-mir-147b	Down	CFB	2	hsa-mir-98-5p
Up	C8orf46	18	hsa-mir-6790-3p	Down	LSS	5	hsa-mir-10a-5p
Up	DSN1	111	hsa-mir-1293	Down	WDR81	36	hsa-mir-6764-5p
Up	GRIK3	53	hsa-mir-3692-5p	Down	RAB17	19	hsa-mir-3183
Up	FANCI	7	hsa-mir-1-3p	Down	TMEM86A	28	hsa-mir-6782-5p
Up	KIF23	60	hsa-mir-148b-5p	Down	CXCL1	4	hsa-mir-27b-5p
Up	BRCA1	22	hsa-mir-1297	Down	SQSTM1	27	hsa-mir-877-5p
Up	RRM2	102	hsa-mir-548ah-5p	Down	ST6GAL1	13	hsa-mir-5009-3p
Up	MSRB3	15	hsa-mir-5096	Down	CLGN	1	hsa-mir-155-5p
Up	THBS1	95	hsa-let-7i-5p	Down	GGCT	4	hsa-mir-30a-5p
Up	FGFR2	5	hsa-mir-125b-5p	Down	CASP4	1	hsa-mir-26b-5p
Up	FANCA	60	hsa-mir-520c-3p	Down	RAB26	2	hsa-mir-421
Up	LIG1	3	hsa-mir-615-3p	Down	PSMG2	14	hsa-mir-2052
Up	PGR	2	hsa-mir-181a-5p	Down	KLHL24	24	hsa-mir-4501
Up	RUNX1T1	51	hsa-mir-202-5p	Down	UNC5B	57	hsa-mir-8089
Up	CST6	1	hsa-mir-335-5p	Down	STARD4	10	hsa-mir-6836-3p
Up	PSMC3IP	4	hsa-mir-193b-3p	Down	ARHGAP12	72	hsa-mir-8063
Up	C21orf58	14	hsa-mir-1227-3p	Down	RHCG	41	hsa-mir-6843-3p
Up	TMEM79	14	hsa-mir-5701	Down	SERPINA3	22	hsa-mir-3909
Up	MARS	14	hsa-mir-505-5p	Down	SLC38A7	105	hsa-mir-1273f
Up	KCNC1	4	hsa-mir-582-5p	Down	TSPYL2	3	hsa-mir-320a
Up	CCNE1	30	hsa-mir-524-5p	Down	QPCT	7	hsa-mir-6880-5p
Up	TEAD1	50	hsa-mir-4748	Down	HS1BP3	1	hsa-mir-124-3p
Up	POLE2	1	hsa-mir-193b-3p	Down	OASL	4	hsa-mir-1-3p
Up	KIFC1	22	hsa-mir-6871-3p	Down	RAB17	19	hsa-mir-6868-3p
Up	E2F2	78	hsa-mir-23b-5p	Down	RAB3IL1	1	hsa-mir-124-3p
Up	MDGA2	10	hsa-mir-664b-3p	Down	ST3GAL1	28	hsa-mir-504-3p
Up	DONSON	19	hsa-mir-5195-5p	Down	IFITM1	19	hsa-let-7c-3p
Up	NCAPG	20	hsa-mir-5696	Down	KMO	8	hsa-mir-4803
Up	HAUS8	35	hsa-mir-4465	Down	GULP1	20	hsa-mir-363-3p
Up	MCM5	8	hsa-mir-93-5p	Down	SHC2	2	hsa-mir-186-5p
Up	IGFBP4	55	hsa-mir-4510	Down	IRS2	26	hsa-mir-3607-5p
Up	GPR37L1	15	hsa-mir-3183	Down	VAT1	31	hsa-mir-4259

Up	AJUBA	3	hsa-mir-196b-5p	Down	CRB3	1	hsa-mir-335-5p
Up	TRIP13	14	hsa-mir-4635	Down	MAPK8IP2	22	hsa-mir-7160-5p
Up	PREP	1	hsa-mir-1301-3p	Down	ISG15	3	hsa-mir-1-1
Up	PTPN14	83	hsa-mir-665	Down	NEURL2	1	hsa-mir-335-5p
Up	GPM6B	16	hsa-mir-3180-5p	Down	IL32	3	hsa-mir-205-5p
Up	UNG	27	hsa-mir-6076	Down	IRF9	1	hsa-mir-106b-5p
Up	MB21D2	32	hsa-mir-4473	Down	CYP51A1	37	hsa-mir-3605-5p
Up	CADM1	42	hsa-mir-4327	Down	TNFSF10	3	hsa-mir-221-3p
Up	DTL	6	hsa-mir-92a-3p	Down	ALDOC	19	hsa-mir-6813-5p
Up	KNTC1	21	hsa-mir-6832-3p	Down	IFIT2	7	hsa-mir-645
Up	RFC3	3	hsa-mir-20a-5p	Down	LDLR	123	hsa-mir-6867-5p
Up	FAM111B	1	hsa-mir-192-5p	Down	ADM2	35	hsa-mir-6890-3p
Up	TCF19	3	hsa-mir-107	Down	FLCN	27	hsa-mir-6847-5p
Up	SAMD4A	40	hsa-mir-1305	Down	BHLHE41	2	hsa-mir-186-5p
Up	FBXO5	5	hsa-mir-151a-3p	Down	SLC36A1	34	hsa-mir-4717-5p
Up	ARHGEF17	11	hsa-mir-4320	Down	RNF152	59	hsa-mir-5186
Up	GINS3	1	hsa-mir-193b-3p	Down	GAB2	12	hsa-mir-218-5p
Up	ABAT	13	hsa-mir-516b-3p	Down	FABP6	1	hsa-mir-335-5p
Up	CENPM	55	hsa-mir-3929	Down	IL32	3	hsa-mir-205-5p
Up	SPAG5	4	hsa-mir-769-5p	Down	SLC7A11	100	hsa-mir-5589-3p
Up	RUNX1	60	hsa-mir-551b-5p	Down	SPIRE1	23	hsa-mir-3976
Up	STARD13	23	hsa-mir-4295	Down	KLHL21	103	hsa-mir-6788-5p
Up	NEDD9	26	hsa-mir-6835-5p	Down	ISG20	3	hsa-mir-1-3p
Up	PMEPA1	44	hsa-mir-374b-5p	Down	IGF2R	54	hsa-mir-7977
Up	POLD1	7	hsa-mir-34a-5p	Down	RRAGC	58	hsa-mir-7114-5p
Up	CDC7	11	hsa-mir-30e-5p	Down	IL15	10	hsa-mir-6780a-3p
Up	MCM4	42	hsa-mir-4284	Down	CCL20	2	hsa-mir-21-5p
Up	GINS4	46	hsa-mir-892a	Down	MAFG	15	hsa-mir-204-5p
Up	ZWINT	98	hsa-mir-577	Down	DHCR7	15	hsa-mir-425-5p
Up	MCM5	8	hsa-mir-193b-3p	Down	OAS2	19	hsa-mir-5192
Up	NETO2	49	hsa-mir-599	Down	QPCT	7	hsa-mir-3689d
Up	FLNB	10	hsa-mir-9-5p	Down	CYBA	2	hsa-mir-320a
Up	ASF1B	9	hsa-mir-3133	Down	ELOVL6	41	hsa-mir-4434
Up	RFC3	3	hsa-mir-124-3p	Down	BBC3	64	hsa-mir-3150b-3p
Up	KDM4B	4	hsa-mir-106b-3p	Down	CXCL3	6	hsa-mir-100-3p
Up	HJURP	24	hsa-mir-6884-5p	Down	CMTM8	1	hsa-mir-877-5p
Up	CUX2	4	hsa-mir-4455	Down	MT1X	2	hsa-mir-1225-3p
Up	DNM1L	8	hsa-mir-519d-3p	Down	SLCO4A1	3	hsa-mir-335-5p
Up	GRIK4	20	hsa-mir-5582-3p	Down	HMOX1	47	hsa-mir-6873-5p
Up	CDCA5	4	hsa-mir-18b-5p	Down	EMP3	3	hsa-mir-26b-5p
Up	PKMYT1	9	hsa-mir-339-5p	Down	MAP1LC3B	41	hsa-mir-4512
Up	KCNC1	4	hsa-mir-582-5p	Down	MAP1LC3B	41	hsa-mir-6730-5p
Up	KIF15	4	hsa-mir-218-5p	Down	ALDH3B2	3	hsa-mir-7-5p
Up	POLA1	5	hsa-mir-6882-3p	Down	CXCL2	5	hsa-mir-223-3p
Up	NUSAP1	1	hsa-let-7b-5p	Down	IFIT1	19	hsa-mir-526b-5p
Up	SIPA1	26	hsa-mir-663a	Down	IFITM1	19	hsa-mir-548g-3p
Up	CHST14	6	hsa-mir-2355-5p	Down	KRTAP5-7	1	hsa-mir-26b-5p
Up	E2F1	41	hsa-mir-6837-5p	Down	TMEM45A	4	hsa-mir-181a-5p
Up	ARHGEF19	5	hsa-mir-6727-3p	Down	TPP1	30	hsa-mir-6841-3p
Up	S100A8	3	hsa-mir-98-5p	Down	S100P	2	hsa-mir-26b-5p
Up	WNT7B	79	hsa-mir-3934-5p	Down	NEURL3	1	hsa-mir-335-5p
Up	ESPL1	34	hsa-let-7f-5p	Down	BEX2	2	hsa-mir-204-5p
Up	SAMD4A	40	hsa-mir-377-3p	Down	CXCL2	5	hsa-mir-223-3p

Up	GRIK4	20	hsa-mir-4652-3p	Down	BIRC3	3	hsa-mir-375
Up	ARHGAP15	24	hsa-mir-548at-3p	Down	HERPUD1	35	hsa-mir-6499-3p
Up	LHX2	2	hsa-mir-196b-5p	Down	IFI44	4	hsa-mir-26b-5p
Up	ORC6	38	hsa-mir-4772-3p	Down	SPIRE1	23	hsa-mir-181b-5p
Up	MCM6	7	hsa-mir-1180-3p	Down	DDX60	3	hsa-mir-1-3p
Up	MAP6D1	2	hsa-mir-215-5p	Down	AHNAK2	17	hsa-mir-6828-5p
Up	ODAM	1	hsa-mir-26b-5p	Down	CCL5	48	hsa-mir-5089-5p
Up	CD24	2	hsa-mir-373-3p	Down	TLR2	7	hsa-mir-154-5p
Up	RAD54L	1	hsa-mir-26b-5p	Down	KCNE4	32	hsa-mir-6847-3p
Up	DHFR	7	hsa-mir-503-5p	Down	DRAM1	6	hsa-mir-181c-5p
Up	S100A9	7	hsa-mir-588	Down	SLC25A36	69	hsa-mir-4668-5p
Up	GRHL3	1	hsa-mir-335-5p	Down	RHOQ	25	hsa-mir-6801-5p
Up	PBK	4	hsa-mir-4699-3p	Down	MT1E	11	hsa-mir-3613-5p
Up	BARD1	10	hsa-mir-6501-3p	Down	SESN2	105	hsa-mir-4748
Up	TBXAS1	2	hsa-mir-34b-3p	Down	LMO2	2	hsa-mir-223-3p
Up	SMTN	6	hsa-mir-548aa	Down	STX3	2	hsa-mir-92a-3p
Up	HIST1H2AL	2	hsa-mir-34a-5p	Down	AKR1C3	2	hsa-mir-155-5p
Up	MCM7	52	hsa-mir-302d-3p	Down	RDH16	1	hsa-mir-7-5p
Up	PDGFB	27	hsa-mir-519a-3p	Down	TNFSF9	55	hsa-mir-3619-5p
Up	ZNF788	37	hsa-mir-302f	Down	HSPBAP1	2	hsa-mir-192-5p
Up	WTIP	22	hsa-mir-4643	Down	MT1X	2	hsa-mir-1225-3p
Up	BLM	3	hsa-mir-192-5p	Down	NEK8	62	hsa-mir-4716-5p
Up	ESCO2	41	hsa-mir-3613-3p	Down	DNAJB9	87	hsa-mir-3064-5p
Up	UHRF1	14	hsa-mir-191-5p	Down	FAM129A	45	hsa-mir-519b-3p
Up	RECQL4	2	hsa-mir-615-3p	Down	SDR16C5	14	hsa-mir-5695
Up	LY6E	26	hsa-mir-6752-5p	Down	NFE2L1	53	hsa-mir-4430
Up	SMOC2	23	hsa-mir-6124	Down	NPHS1	13	hsa-mir-6790-3p
Up	BANK1	4	hsa-mir-590-3p	Down	APOL2	18	hsa-mir-5094
Up	TK1	26	hsa-mir-4752	Down	NAMPT	15	hsa-mir-4452
Up	CCNA2	36	hsa-mir-301b-3p	Down	SLC25A36	69	hsa-mir-548az-5p
Up	MCM2	12	hsa-mir-500a-5p	Down	LHFPL2	65	hsa-mir-6856-5p
Up	TRIM6	1	hsa-mir-26b-5p	Down	BTN3A1	8	hsa-mir-17-5p
Up	KIF11	15	hsa-mir-4495	Down	MT1F	3	hsa-mir-219a-5p
Up	CYP2J2	1	hsa-let-7b-5p	Down	SLC33A1	49	hsa-mir-7109-3p
Up	RUSC1	3	hsa-mir-425-5p	Down	HSD17B14	1	hsa-mir-26b-5p
Up	ACOT7	4	hsa-mir-18a-3p	Down	GADD45G	1	hsa-mir-181a-5p
Up	FERMT1	4	hsa-mir-9-5p	Down	SPRR1A	1	hsa-mir-26b-5p
Up	DSCC1	3	hsa-mir-3163	Down	IL6R	55	hsa-mir-4753-3p
Up	NFIB	89	hsa-mir-520e	Down	INHBE	2	hsa-mir-98-5p
Up	MMS22L	35	hsa-mir-4789-5p	Down	CSAG1	3	hsa-mir-222-3p
Up	DCLRE1B	5	hsa-mir-320b	Down	SCGB1D1	2	hsa-mir-335-5p
Up	INHBA	40	hsa-mir-548au-5p	Down	PRNP	57	hsa-mir-301b-3p
Up	AURKB	10	hsa-mir-16-5p	Down	CIITA	24	hsa-mir-6753-5p
Up	CENPM	55	hsa-mir-6500-3p	Down	GBP2	6	hsa-mir-433-3p
Up	CHAF1B	80	hsa-mir-3924	Down	RNF43	1	hsa-mir-335-5p
Up	GINS1	6	hsa-mir-18a-3p	Down	TUBB2B	7	hsa-mir-877-5p
Up	KIF20A	5	hsa-mir-215-5p	Down	HYOU1	29	hsa-mir-6739-5p
Up	FLNA	24	hsa-mir-200c-3p	Down	HSPA5	41	hsa-mir-548x-3p
Up	PTGER3	11	hsa-mir-519e-5p	Down	TMEM97	21	hsa-mir-6833-5p
Up	SEMA3G	9	hsa-mir-4287	Down	IFIH1	4	hsa-mir-15b-5p
Up	HIST1H1D	4	hsa-mir-193b-3p	Down	GADD45A	12	hsa-mir-4678
Up	LCAT	6	hsa-mir-6836-3p	Down	APOL6	106	hsa-mir-665
Up	RHBDL2	24	hsa-mir-1281	Down	NR1D1	1	hsa-mir-335-5p

Up	NRGN	24	hsa-mir-3141	Down	JMY	61	hsa-mir-4473
Up	MKI67	85	hsa-mir-520g-3p	Down	WARS	39	hsa-mir-4728-5p
Up	KCNC3	32	hsa-mir-6736-3p	Down	VEGFA	108	hsa-mir-4735-5p
Up	TRAM2	41	hsa-mir-4428	Down	VEGFA	108	hsa-mir-3924
Up	ALDH7A1	13	hsa-mir-5585-3p	Down	GADD45G	1	hsa-mir-181a-5p
Up	DOK7	2	hsa-mir-375	Down	SAT1	10	hsa-mir-4477a
Up	RFC5	20	hsa-mir-6729-3p	Down	PEG10	139	hsa-mir-8072
Up	RASSF7	1	hsa-mir-196a-5p	Down	UPP1	2	hsa-mir-192-5p
Up	FLT4	2	hsa-mir-1236-3p	Down	UVRAG	9	hsa-mir-130b-3p
Up	SPIN4	54	hsa-mir-3680-3p	Down	S100A14	2	hsa-mir-193b-3p
Up	PLCE1	68	hsa-mir-6807-5p	Down	SQLE	3	hsa-mir-205-5p
Up	CTGF	24	hsa-mir-5580-3p	Down	FAM83G	61	hsa-mir-7854-3p
Up	CIT	25	hsa-mir-6854-3p	Down	FICD	62	hsa-mir-5089-3p
Up	GTSE1	2	hsa-mir-4524b-3p	Down	ICAM4	1	hsa-mir-93-5p
Up	RASL11B	7	hsa-mir-4743-3p	Down	SEC24D	28	hsa-mir-6131
Up	EGLN3	37	hsa-mir-4762-5p	Down	CBR3	1	hsa-mir-124-3p
Up	MYBL1	59	hsa-mir-1277-5p	Down	GULP1	20	hsa-mir-367-3p
Up	XRCC3	45	hsa-mir-4651	Down	KMO	8	hsa-mir-3143
Up	ZFP36L2	32	hsa-mir-3174	Down	PDE2A	2	hsa-mir-192-5p
Up	RAD51AP1	10	hsa-mir-561-3p	Down	RSAD2	2	hsa-mir-26b-5p
Up	CACNG4	1	hsa-mir-335-5p	Down	HRK	62	hsa-mir-6758-3p
Up	TRPM8	1	hsa-mir-335-5p	Down	SAT1	10	hsa-mir-652-3p
Up	MYL9	13	hsa-mir-6840-3p	Down	CERS1	42	hsa-mir-765
Up	LRP4	3	hsa-mir-148b-3p	Down	ZNF165	2	hsa-mir-215-5p
Up	XYLT1	1	hsa-mir-16-5p	Down	KRT75	2	hsa-mir-1-1
Up	PCP4L1	55	hsa-mir-4492	Down	MT1A	41	hsa-mir-939-3p
Up	ID3	23	hsa-mir-7151-5p	Down	FOXD1	3	hsa-mir-192-5p
Up	TONSL	11	hsa-mir-320a	Down	RHOQ	25	hsa-mir-7157-5p
Up	GREB1	34	hsa-mir-5189-3p	Down	HK2	7	hsa-mir-125b-5p
Up	FOLR1	70	hsa-mir-4272	Down	IRF1	33	hsa-mir-640
Up	CDT1	48	hsa-mir-1343-3p	Down	UPP1	2	hsa-mir-215-5p
Up	IGFBP5	143	hsa-mir-3176	Down	SRXN1	15	hsa-mir-499b-3p
Up	PTPN14	83	hsa-mir-450a-2-3p	Down	PLA2G4C	3	hsa-mir-132-3p
Up	SLC23A1	30	hsa-mir-562	Down	MT2A	11	hsa-mir-4668-3p
Up	CDKN2C	1	hsa-mir-34a-5p	Down	NAMPT	15	hsa-mir-4422
Up	CISH	3	hsa-mir-150-5p	Down	SOD2	257	hsa-mir-651-3p
Up	TPM1	22	hsa-mir-6828-3p	Down	SLCO4C1	26	hsa-mir-6752-3p
Up	ANLN	4	hsa-mir-424-5p	Down	MAPK6	75	hsa-mir-1252-3p
Up	PTGER3	11	hsa-mir-5003-3p	Down	LTB	2	hsa-mir-122-5p
Up	JPH2	52	hsa-mir-1205	Down	TNFRSF10B	91	hsa-mir-6878-5p
Up	PREX1	4	hsa-mir-27a-3p	Down	FADS3	12	hsa-mir-6505-3p
Up	ANKRD2	1	hsa-mir-27b-3p	Down	C10orf10	20	hsa-mir-7158-5p
Up	ETNK2	5	hsa-mir-132-3p	Down	SAMD9L	34	hsa-mir-3175
Up	TEAD4	2	hsa-mir-181a-5p	Down	BDKRB1	1	hsa-mir-124-3p
Up	POLA2	62	hsa-mir-1185-1-3p	Down	CDKN1C	5	hsa-mir-221-3p
Up	OLFML3	1	hsa-mir-335-5p	Down	GTPBP2	72	hsa-mir-548as-5p
Up	SERTAD4	19	hsa-mir-6873-3p	Down	CEBPB	25	hsa-mir-4677-5p
Up	TRIM58	20	hsa-mir-1912	Down	RELB	1	hsa-mir-26b-5p
Up	PHF19	97	hsa-mir-3126-3p	Down	LARP6	1	hsa-mir-744-5p
Up	ITPKA	1	hsa-mir-193b-3p	Down	SLC43A1	18	hsa-mir-7111-3p
Up	BFSP2	1	hsa-mir-335-5p	Down	BCL2L11	76	hsa-mir-8064
Up	SLC40A1	1	hsa-mir-485-3p	Down	C2CD4A	8	hsa-mir-4659b-3p
Up	MND1	1	hsa-mir-193b-3p	Down	PLAU	7	hsa-mir-193a-3p

Up	TMEM229B	10	hsa-mir-4257	Down	KLF4	20	hsa-mir-663a
Up	PTGER3	11	hsa-mir-519d-5p	Down	CYP1A2	15	hsa-mir-1292-3p
Up	E2F8	45	hsa-mir-190a-3p	Down	ITPRIP	25	hsa-mir-190a-3p
Up	CDCA3	23	hsa-mir-4308	Down	IFIT2	7	hsa-let-7c-5p
Up	KIAA1161	38	hsa-mir-5698	Down	TNFAIP3	65	hsa-mir-4659a-3p
Up	PTPN14	83	hsa-mir-3667-3p	Down	EIF2AK3	5	hsa-mir-21-5p
Up	BOP1	2	hsa-mir-296-3p	Down	SPAG9	32	hsa-mir-578
Up	KRT8	42	hsa-mir-4419b	Down	RORA	110	hsa-mir-548aj-5p
Up	SPC24	26	hsa-mir-6888-3p	Down	NDRG1	52	hsa-mir-6839-5p
Up	RASSF2	32	hsa-mir-3120-5p	Down	TRIM16L	25	hsa-mir-1273g-3p
Up	KIF5C	20	hsa-mir-4789-3p	Down	IFRD1	45	hsa-mir-6129
Up	CLIC3	2	hsa-mir-124-3p	Down	JMY	61	hsa-mir-6832-3p
Up	CKAP2L	38	hsa-mir-3612	Down	ALDH1L2	10	hsa-mir-6727-3p
Up	FRMD3	13	hsa-mir-7110-3p	Down	PABPC1L	1	hsa-mir-335-5p
Up	C2orf71	12	hsa-mir-4657	Down	RAB39B	21	hsa-mir-1185-2-3p
Up	DHFR	7	hsa-mir-15b-5p	Down	DNAJC10	195	hsa-mir-8053
Up	PTGER3	11	hsa-mir-514b-3p	Down	TMEM140	1	hsa-mir-335-5p
Up	PLCE1	68	hsa-mir-2467-5p	Down	GTPBP2	72	hsa-mir-548o-5p
Up	ACTBL2	12	hsa-mir-3161	Down	VLDLR	76	hsa-mir-4433b-5p
Up	ACOX1	98	hsa-mir-6788-3p	Down	SLC6A12	2	hsa-mir-342-3p
Up	WISP2	2	hsa-mir-449a	Down	SLC5A3	68	hsa-mir-548ba
Up	HOXC12	2	hsa-mir-9-5p	Down	IL21R	37	hsa-mir-6763-5p
Up	REEP5	40	hsa-mir-6735-5p	Down	MAFF	17	hsa-mir-4466
Up	SLITRK4	34	hsa-mir-223-5p	Down	ZNF365	3	hsa-mir-223-3p
Up	LYPD5	1	hsa-mir-375	Down	PARP14	1	hsa-mir-124-3p
Up	IVL	1	hsa-mir-335-5p	Down	BDKRB2	13	hsa-mir-6809-3p
Up	ERCC6L	26	hsa-mir-6722-3p	Down	ARID5A	1	hsa-mir-324-5p
Up	MYBL1	59	hsa-mir-592	Down	ATF3	22	hsa-mir-6512-3p
Up	GRPR	1	hsa-mir-17-3p	Down	SBSN	1	hsa-mir-106b-5p
Up	IGDCC3	16	hsa-mir-4326	Down	NAMPT	15	hsa-mir-32-3p
Up	SLC43A3	56	hsa-mir-5010-3p	Down	PPP1R15A	20	hsa-mir-6756-5p
Up	RBBP8	19	hsa-mir-658	Down	MT1M	1	hsa-mir-26b-5p
Up	LMNB1	78	hsa-mir-449c-3p	Down	MIA2	3	hsa-mir-1-3p
Up	ID1	8	hsa-mir-520h				
Up	NCAM2	21	hsa-mir-6507-5p				
Up	SERPINF1	1	hsa-mir-335-5p				
Up	S100A16	3	hsa-mir-1-3p				
Up	SKA3	2	hsa-mir-193b-3p				
Up	COL27A1	5	hsa-mir-135a-3p				
Up	BUB1B	4	hsa-mir-192-5p				
Up	FBXL18	63	hsa-mir-1470				
Up	CLPSL1	12	hsa-mir-765				
Up	PYROXD2	1	hsa-mir-335-5p				
Up	ELOVL2	3	hsa-mir-17-3p				
Up	CCL2	6	hsa-mir-24-3p				
Up	RABIF	26	hsa-mir-1260a				

Degree – No of miRNA interact with target gene. We taken any one miRNA in table.

Table S8: TF - target gene interaction table

Regulation	Genes	Degree	TF	Regulation	Genes	Degree	TF
Up	ID3	2	BCL11B	Down	VEGFA	1	BP1
Up	BRCA1	16	E2F7	Down	C10orf10	1	HOXA2
Up	LIG1	1	TCF21	Down	IL6R	2	GLI1
Up	GRHL3	1	ZNF652	Down	LDLR	1	BCL11B
Up	ITPKA	2	ZNF263	Down	F7	4	TCF21
Up	CALD1	4	IKZF1	Down	LDLR	1	ZNF652
Up	MCM10	6	ZNF322	Down	SQLE	3	ZNF263
Up	KIF15	2	PRDM16	Down	DNAJC10	2	IKZF1
Up	ZNF788	3	ZNF274	Down	NEURL2	6	ZNF274
Up	TRIM6	6	CHD7	Down	IRF1	2	CHD7
Up	ZWINT	1	SALL1	Down	IDH1	3	SALL1
Up	RUNX1T1	4	NR4A2	Down	AMDHD2	3	NR4A2
Up	SAMD4A	7	CDKN2AIP	Down	TSPYL2	1	CDKN2AIP
Up	LHFP	5	THRA	Down	HERPUD1	4	THRA
Up	PCNA	6	HOXD13	Down	UPP1	3	HOXD13
Up	AURKB	20	FOXM1	Down	RHOQ	4	FOXM1
Up	PGR	4	HTT	Down	BEX2	10	HTT
Up	CITED1	3	ETS2	Down	ATF3	3	ETS2
Up	XYLT1	6	GBX2	Down	WDSUB1	3	GBX2
Up	WNT7B	13	DROSHA	Down	FRY	7	DROSHA
Up	UNG	3	FOXO1	Down	ST3GAL1	10	FOXO1
Up	S100A8	6	RARG	Down	GDF15	15	RARG
Up	CDKN2C	8	RCOR2	Down	MAPK8IP2	6	RCOR2
Up	RUNX1	9	HIF1A	Down	DDIT4	14	HIF1A
Up	TGFB2	22	ESR2	Down	SQSTM1	16	ESR2
Up	ATAD2	7	STAT6	Down	TNFAIP3	14	STAT6
Up	MCM3	15	NR1H3	Down	MT1X	31	NR1H3
Up	BUB1B	3	NOTCH1	Down	PPP1R15A	9	NOTCH1
Up	STARD13	10	SREBF1	Down	MAPK6	16	SREBF1
Up	HIST1H1B	13	THAP11	Down	CLCN7	5	THAP11
Up	CDC45	5	HCFC1	Down	IFRD1	1	HCFC1
Up	ID1	6	CLOCK	Down	FLCN	19	CLOCK
Up	TMEM229B	17	IRF8	Down	TMEM97	15	NFIB
Up	MCM7	8	IRF1	Down	GADD45G	32	IRF8
Up	S100A16	6	CRX	Down	CHN2	11	CRX
Up	TCF4	14	DNAJC2	Down	IL32	20	DNAJC2
Up	COL12A1	18	PAX6	Down	KLF4	10	PAX6
Up	HIST1H1D	9	STAT1	Down	BTN3A1	6	STAT1
Up	HIST1H3F	32	RBPJ	Down	JMY	28	RBPJ
Up	C16orf59	14	ELF1	Down	SAT2	28	ELF1
Up	KIF20A	14	PDX1	Down	SLC25A36	16	PDX1
Up	HIST1H3B	25	PADI4	Down	ALDH3B2	13	PADI4
Up	RAD51AP1	13	KDM6A	Down	KRTAP5-7	6	KDM6A

Up	MCM5	14	DCP1A	Down	HOXB9	14	DCP1A
Up	MCM2	9	TFEB	Down	RRAGC	35	TFEB
Up	TRAM2	8	ASXL1	Down	NEAT1	12	ASXL1
Up	EFEMP1	16	TCF7L2	Down	LHFPL2	10	TCF7L2
Up	COL27A1	16	NR1I2	Down	RARRES1	13	NR1I2
Up	SLC12A4	18	GATA3	Down	OASL	19	GATA3
Up	TMEM139	20	PRDM5	Down	DNAJB9	23	PRDM5
Up	ELOVL2	18	SREBF2	Down	CCDC154	23	SREBF2
Up	CDC25A	32	CEBPB	Down	C10orf10	21	CEBPA
Up	SERPINF1	9	CEBPA	Down	GULP1	22	CDX2
Up	ZFP36L2	18	CDX2	Down	APOL6	22	TAF7L
Up	AMOTL2	11	TAF7L	Down	RAB24	15	BCL3
Up	HR	11	BCL3	Down	ICAM1	11	SMAD1
Up	HIST1H4D	22	SMAD1	Down	SLCO4C1	6	ZIC3
Up	FBXO5	5	ZIC3	Down	DHCR7	36	NR0B1
Up	CTGF	40	NR0B1	Down	TUBB2B	13	NACC1
Up	RFC5	22	NACC1	Down	STX3	21	SOX11
Up	POLA2	26	SOX11	Down	TSKU	31	STAT5A
Up	TEAD1	31	STAT5A	Down	NFE2L1	18	TBP
Up	HIST1H2BF	15	TBP	Down	WARS	19	HSF1
Up	ESCO2	22	HSF1	Down	FUT3	52	BACH1
Up	MCM4	48	BACH1	Down	NPHS1	26	EWSR1
Up	GINS4	27	EWSR1	Down	IRS2	16	ELF5
Up	S100A9	25	ELF5	Down	INHBE	71	RELA
Up	TPM1	19	RELA	Down	MAF	24	NUCKS1
Up	CCNE1	9	NUCKS1	Down	MIA2	19	RCOR1
Up	E2F8	29	RCOR1	Down	ZNF365	23	POU3F2
Up	CCNE2	23	POU3F2	Down	HMOX1	35	MEIS1
Up	RFC2	20	MEIS1	Down	RHCG	15	LYL1
Up	DIAPH3	17	LYL1	Down	EMP3	29	MYB
Up	HOXC12	31	LMO2	Down	NOS3	26	SIN3A
Up	MCM6	24	MYB	Down	RAB33A	26	PBX1
Up	CHAF1B	21	SIN3A	Down	CERS1	31	JUN
Up	FAM111B	33	PBX1	Down	PPARGC1A	18	TBX3
Up	CCL2	28	JUN	Down	TESK2	23	CTNNB1
Up	C19orf57	25	TBX3	Down	MT2A	31	TFAP2A
Up	BARD1	34	CTNNB1	Down	PLAU	30	ESRRB
Up	ACOX1	47	TFAP2A	Down	UCN	85	SUZ12
Up	ALDH1A3	35	ESRRB	Down	LARP6	54	MTF2
Up	DONSON	109	SUZ12	Down	CDKN1C	22	JARID2
Up	IGFBP4	72	MTF2	Down	PRR15	36	RNF2
Up	IGFBP5	40	JARID2	Down	FOXD1	40	EZH2
Up	NRGN	49	RNF2	Down	BCL2L11	15	PHC1
Up	FBXL18	47	EZH2	Down	CD70	12	EED
Up	CDCA3	20	PHC1	Down	SPIRE1	36	BMI1
Up	FLT4	23	EED	Down	RNF43	40	RAD21
Up	SEMA3G	29	BMI1	Down	HS1BP3	24	CTCF
Up	RFC3	43	RAD21	Down	FTL	24	TCF7

Up	CCDC85A	36	CTCF	Down	STARD4	29	FOXP2
Up	FLNB	33	TCF7	Down	PLEKHA4	34	TBX5
Up	CIT	34	FOXP2	Down	TPP1	24	SRF
Up	GINS3	34	TBX5	Down	ATP2C2	15	MEF2A
Up	HSPB11	20	SRF	Down	ARID5A	32	GATA4
Up	MSRB3	21	MEF2A	Down	NR1D1	39	STAT4
Up	MYBL2	43	GATA4	Down	NUCB2	51	MECOM
Up	GPM6B	27	STAT4	Down	NEK8	16	ZFP42
Up	BLM	35	MECOM	Down	MAP1LC3B	27	YY1
Up	DTL	29	ZFP42	Down	FDPS	17	CHD1
Up	BOP1	40	YY1	Down	HSPBAP1	31	MYBL2
Up	FANCA	20	CHD1	Down	KLHL24	27	HOXB4
Up	AJUBA	31	HOXB4	Down	ELOVL6	42	CCND1
Up	CYR61	43	CCND1	Down	UNC5B	42	SCLY
Up	DOK7	41	SCLY	Down	CMTM8	54	CUX1
Up	CDC7	62	CUX1	Down	IL15	40	RUNX2
Up	CDH18	61	RUNX2	Down	QPCT	34	NR3C1
Up	DCN	41	NR3C1	Down	AKR1C1	68	SMAD3
Up	ANLN	68	SMAD3	Down	SPAG9	54	SMAD2
Up	KIAA1161	48	SMAD2	Down	CBR3	38	TFCP2L1
Up	JPH2	45	ATF3	Down	ICAM4	36	KLF1
Up	DNM1L	47	TFCP2L1	Down	BDKRB2	32	PRDM14
Up	HELLS	51	KLF1	Down	MSMO1	24	TEAD4
Up	ANKRD2	33	PRDM14	Down	IGF2R	37	DMRT1
Up	ASF1B	41	DMRT1	Down	TNFRSF10B	45	ZNF281
Up	CACNG4	39	ZNF281	Down	SYPL2	43	WT1
Up	ABAT	39	WT1	Down	FTHL17	31	SOX17
Up	FOLR1	46	SOX17	Down	ARHGAP12	63	SALL4
Up	BFSP2	74	SALL4	Down	PLA2G3	39	YAP1
Up	INHBA	51	YAP1	Down	GM2A	59	SMARCA4
Up	CDH10	55	SMARCA4	Down	RORA	30	OLIG2
Up	EGLN3	42	OLIG2	Down	SERPINA3	52	ESR1
Up	EGFL6	69	ESR1	Down	BBC3	44	EP300
Up	H19	55	EP300	Down	MX1	41	FOXO3
Up	KIFC1	40	FOXO3	Down	DBP	57	SRY
Up	GPR20	69	SRY	Down	FAM129A	75	SOX9
Up	SULF2	69	SOX9	Down	VAT1	65	TRIM28
Up	KCNC3	88	TRIM28	Down	COL16A1	21	CNOT3
Up	MAP6D1	36	CNOT3	Down	CCR10	65	TAL1
Up	ORC6	59	TAL1	Down	RNF43	33	GFI1B
Up	LMNB1	24	GFI1B	Down	IRF9	65	ERG
Up	TNNI2	55	ERG	Down	CYP51A1	46	MYCN
Up	MYCBP	63	MYCN	Down	MAFF	58	TET1
Up	PREP	68	TET1	Down	DDIT3	73	PPARD
Up	PLXNA1	85	PPARD	Down	NEU1	62	FOXP1
Up	LRP4	65	FOXP1	Down	DUSP13	75	TFAP2C
Up	UHRF1	79	TFAP2C	Down	IL23A	50	EOMES
Up	WTIP	50	EOMES	Down	FEZ1	38	ASH2L

Up	NEDD9	62	ASH2L	Down	CEACAM1	74	SETDB1
Up	SYNPO	79	SETDB1	Down	MAFG	74	REST
Up	IGDCC3	81	REST	Down	ADM2	66	SIN3B
Up	TRH	73	SIN3B	Down	HSPA5	46	RCOR3
Up	RABIF	61	RCOR3	Down	HPS1	39	E2F4
Up	XRCC3	116	E2F4	Down	PEG10	47	ZFX
Up	REEP5	50	ZFX	Down	SQLE	49	KDM5B
Up	NRAP	81	KDM5B	Down	DAPP1	104	GATA2
Up	RHBDL2	85	GATA2	Down	CIITA	90	GATA1
Up	SPC24	82	GATA1	Down	SEPHS2	124	HNF4A
Up	SERPINA6	115	HNF4A	Down	S100P	61	FOXA2
Up	KRTAP3-1	64	FOXA2	Down	S100A3	82	PPARG
Up	KRT8	95	PPARG	Down	SBSN	93	TP53
Up	ANKRD2	122	TP53	Down	HSD17B14	132	MITF
Up	CRYM	99	MITF	Down	CFB	137	EGR1
Up	SMTN	146	EGR1	Down	HRK	65	TCF3
Up	MB21D2	79	TCF3	Down	MT1H	172	SOX2
Up	TBXAS1	177	SOX2	Down	SDR16C5	107	POU5F1
Up	CENPM	137	POU5F1	Down	LY96	121	NANOG
Up	MCEE	154	NANOG	Down	RETN	143	SPI1
Up	TONSL	109	KLF4	Down	KRTAP5-4	163	MYC
Up	ETNK2	144	SPI1	Down	SOD2	122	RUNX1
Up	DHFR	178	MYC	Down	TLR2	98	FLI1
Up	MARS	92	FLI1	Down	EBP	97	E2F1
Up	KIF5C	100	CREM	Down	GTPBP2	76	CREM
Up	NDC80	85	CREB1	Down	ACSS2	79	CREB1
Up	C2orf71	120	TP63	Down	MT1L	120	TP63
Up	IVL	92	SMAD4	Down	SDR16C5	65	SMAD4
Up	ARHGEF19	125	STAT3	Down	BEST1	46	TCF4
Up	VTCN1	124	AR	Down	VSTM1	109	STAT3
Up	DCLRE1B	33	PHF8	Down	C15orf48	108	AR
Up	CDCA5	32	DACH1	Down	LGALSL	23	PHF8
Up	OLFM1	21	ELK1	Down	C6ORF1	24	DACH1
Up	RECQL4	24	XRN2	Down	KMO	25	ELK1
Up	WISP2	27	TTF2	Down	EIF2AK3	25	XRN2
Up	PLCE1	24	FOXP3	Down	PLD6	23	TTF2
Up	RAD51	27	VDR	Down	RAB39B	20	FOXP3
Up	GTSE1	30	HOXC9	Down	LAMP3	47	VDR
Up	SEC14L2	21	KDM5A	Down	APOL2	22	HOXC9
Up	EXO1	18	ETS1	Down	RENBP	11	KDM5A
Up	PSMC3IP	11	AP1S2	Down	ZNF165	12	ETS1
Up	LRRC4C	32	NFE2L2	Down	PSMG2	3	AP1S2
Up	MSH6	15	CEBPD	Down	SLC7A11	43	NFE2L2
Up	NFIB	25	PAX3	Down	DNAJC10	11	CEBPD
Up	SULT2B1	46	ZNF217	Down	CTAGE1	21	PAX3
Up	RMI2	29	ARNT	Down	ATP2C2	30	ZNF217
Up	MS4A7	21	AHR	Down	CMTM8	26	ARNT
Up	RABIF	1	KLF5	Down	CYP1A2	22	AHR

Up	RABIF	1	KLF2	Down	KLF4	3	KLF5
				Down	HOXB9	3	KLF2

Degree – No of target gene interact with TF. We taken any one target gene in table. TF transcription factors
